# Petrosal morphology and cochlear function in Mesozoic stem therians

**DOI:** 10.1371/journal.pone.0209457

**Published:** 2019-08-14

**Authors:** Tony Harper, Guillermo W. Rougier

**Affiliations:** 1 Center for Functional Anatomy and Evolution, Johns Hopkins University, Baltimore, Maryland, United States of America; 2 Department of Anatomical Sciences and Neurobiology, University of Louisville, Louisville, Kentucky, United States of America; Monash University, AUSTRALIA

## Abstract

Here we describe the bony anatomy of the inner ear and surrounding structures seen in three plesiomorphic crown mammalian petrosal specimens. Our study sample includes the triconodont *Priacodon fruitaensis* from the Upper Jurassic of North America, and two isolated stem therian petrosal specimens colloquially known as the Höövör petrosals, recovered from Aptian-Albian sediments in Mongolia. The second Höövör petrosal is here described at length for the first time. All three of these petrosals and a comparative sample of extant mammalian taxa have been imaged using micro-CT, allowing for detailed anatomical descriptions of the osteological correlates of functionally significant neurovascular features, especially along the abneural wall of the cochlear canal. The high resolution imaging provided here clarifies several hypotheses regarding the mosaic evolution of features of the cochlear endocast in early mammals. In particular, these images demonstrate that the membranous cochlear duct adhered to the bony cochlear canal abneurally to a secondary bony lamina before the appearance of an opposing primary bony lamina or tractus foraminosus. Additionally, while corroborating the general trend of reduction of venous sinuses and plexuses within the pars cochlearis seen in crownward mammaliaforms generally, the Höövör petrosals show the localized enlargement of a portion of the intrapetrosal venous plexus. This new vascular feature is here interpreted as the bony accommodation for the vein of cochlear aqueduct, a structure that is solely, or predominantly, responsible for the venous drainage of the cochlear apparatus in extant therians. Given that our fossil stem therian inner ear specimens appear to have very limited high-frequency capabilities, the development of these modern vascular features of the cochlear endocast suggest that neither the initiation or enlargement of the stria vascularis (a unique mammalian organ) was originally associated with the capacity for high-frequency hearing or precise sound-source localization.

## Introduction

Therian mammals (the last common ancestor of marsupials and placentals and its descendants [1]) today display an impressive variety of auditory characteristics facilitating the adept detection of airborne sound, making this group arguably the most acoustically sophisticated and diverse clade of terrestrial vertebrates [2]. The widespread capacity for high-acuity hearing (in terms of sensitivity, specificity, and highest detectable frequency) among the majority of extant therian mammals has reinforced the assumption among neontologists that the Mesozoic members of the therian stem linage were stereotypically nocturnal forms that leveraged their auditory faculties to locate small prey and escape gigantic predators (i.e. [3]). However, this supposition has not been supported by the known fossil record of stem therians, with prior descriptions [4–9] demonstrating that these forms lacked the level of petrosal organization characterizing plesiomorphic marsupials [10], afrotheres [11], eulipotyphlans, and primatomorphs [12]. Additionally, many of these older reports lacked CT imaging, reliying heavily on the description of the external morphology of the ear region. Therefore, much of the anatomical detail informing the performance and/or physiological evolution of the inner ear remained understandably inaccessible.

Conversely, several biomechanical/experimental studies on auditory anatomy and physiology across extant tetrapods [2, 13–16] highlight the unique nature of the therian cochlear apparatus (with its well-ordered acoustic hair-cell populations arrayed along the organ of Corti, high endolymphatic potential, and absence of the lagenar macula), as well as the plesiomorphic nature of the monotreme cochlea with respect to many modern and fossil therians [17,18]. The complete loss of the lagenar macula in particular has been posited as a adaptive breakthrough that permitted later elongation and sophistication of the cochlear apparatus for non-linear amplification of high-frequency stimuli [19]. The plesiomorphic retention of a functional lagenar macula, along with its accompanying otoconial and neurovascular structures, in the monotreme and sauropsid lineages is also physiologically incompatible with several soft tissue adaptations seen in modern therians. These “therian” features include: 1) the exclusive reliance on the stria vascularis as the major endolymph producing organ, 2) the well-developed electromotility of prestins and other molecular components of the “cochlear amplifier”, and 3) the radical elongation and geometrical reorganization of the cochlear sensory epithelium. The accumulation of these characteristics among therian ancestors points to a fundamental transformation of the cochlear apparatus somewhere near the origin of Theria [19].

**Fig 1:**
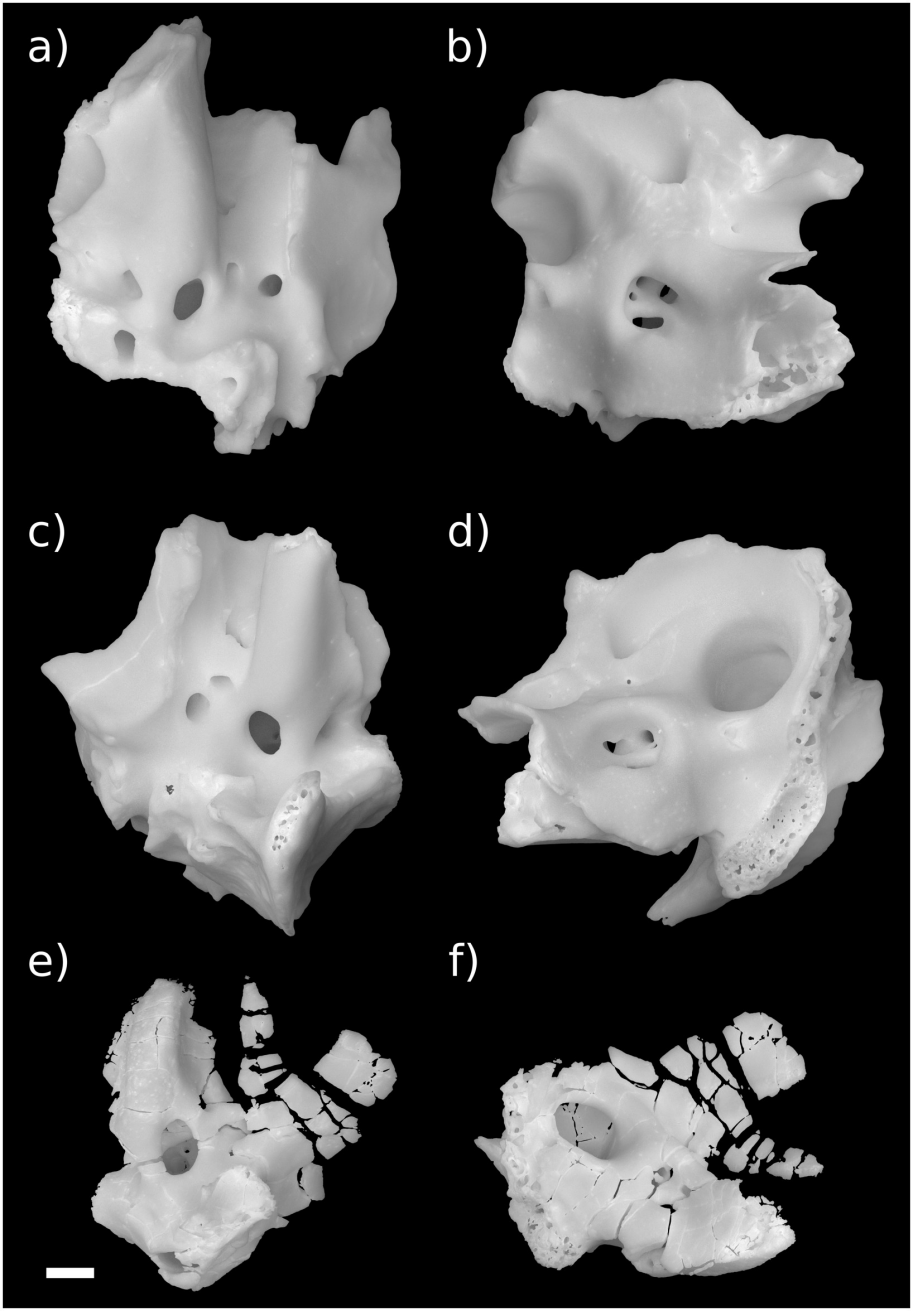
Stem therian petrosal specimens described in this report. a, b Höövör Petrosal 2 (H2; PSS-MAE-119); c, d, Höövör Petrosal 1 (H1; PSS-MAE-104); e,f left petrosal of *Priacodon fruitaensis* (LACM 120451). a, c, e in ventral view; b, d, f in dorsomedial view. Scale bar is 1 mm.

The lack of detailed cochlear reconstructions for Mesozoic fossils is understandable given the general lack of high-fidelity bony correlates for key soft tissue structures such as the cochlear duct, lagenar macula, and stria vascularis. This report uses high-resolution micro-CT information to update descriptions of petrosal anatomy provided in the representative sample of stem therians focused on by [4] and [5], two large-scale studies characterizing the fossil record of early mammalian petrosal evolution (Fig 1). These images and reconstructions are framed within a broader comparative and functional setting of modern mammalian auditory physiology, and morphology with the hope of bridging both fields which, due to the steep learning curves involved, have had only limited cross-referencing. The new high-resolution images presented here allow the first descriptions of the labyrinthine anatomy in some of the most morphologically plesiomorphic petrosal regions known in the crown mammalian fossil record. The three specimens focused on here include the relatively well-known triconodontid *Priacodon fruitaensis* and the two isolated petrosal specimens known as the Höövör petrosals. The second Höövör petrosal (Fig 1a and 1b) has not been fully described or figured, and has only been cursorily referred to in [4] and briefly discussed in [5]. We regard this second petrosal as taxonomically distinct from that of the previously described petrosal in [4], and therefore provide here a complete description and assessment of this specimen.

**Fig 2:**
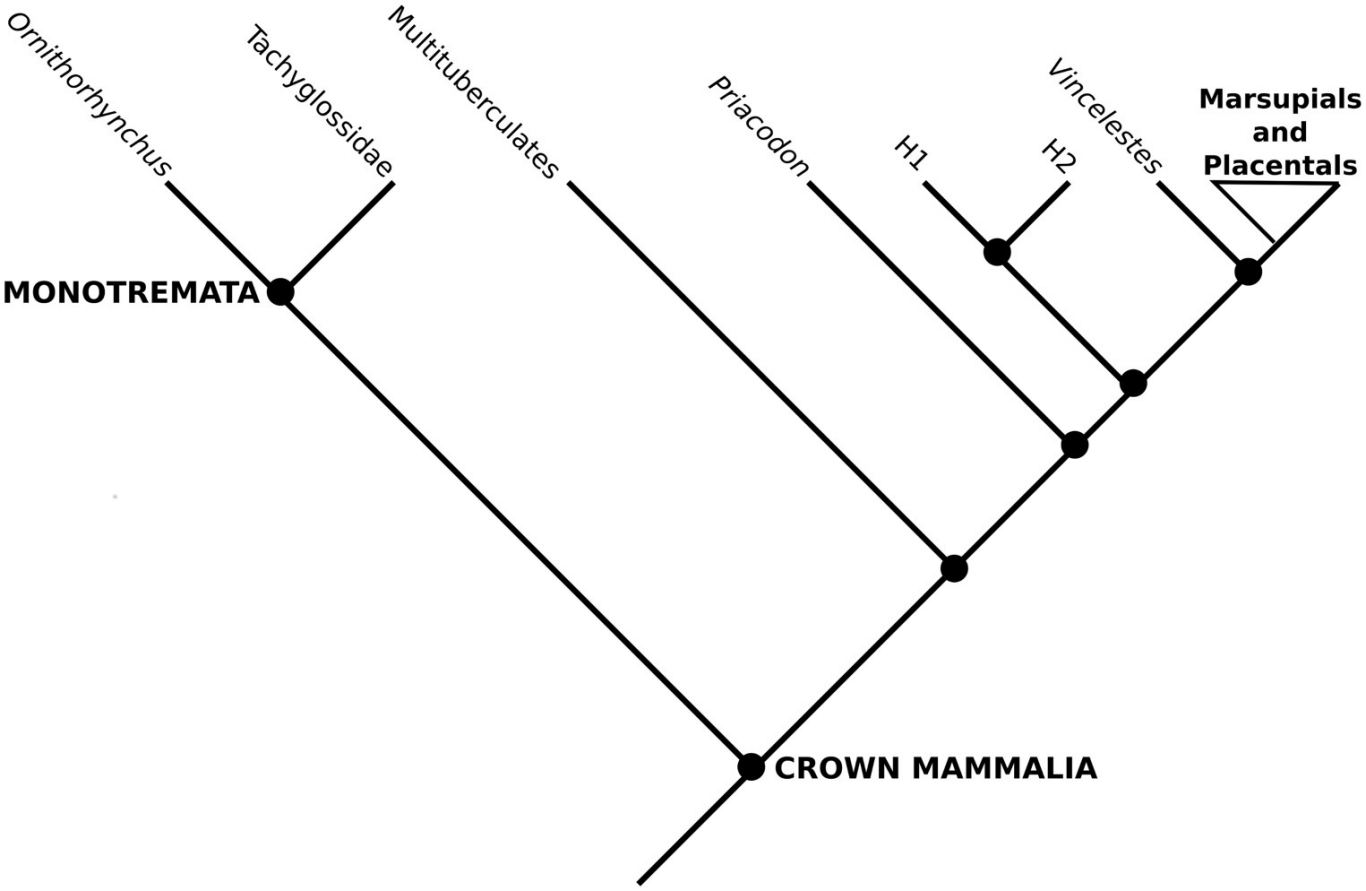
Simplified phylogeny of mammalia. Example phylogeny showing phylogenetic locations of the stem therian petrosals described here. Toplogy simplified from Rescaled Consistency Index PAUP analysis presented in [5].

The novel information presented here does little to overturn previous taxonomic arrangements of these stem therians because they only slightly change their scorings in previous matrices [5]; and because most details of inner ear anatomy is yet to be distilled into characters/character states to be used in a phylogenetic estimation (Fig 2). However, the new anatomical representation provided by the images used here demonstrate the presence of a combination of plesiomorphic and derived character states of the cochlear canal that support their shared ancestry with crown therian mammals. In particular, the presence of a secondary lamina, and in the Höövör petrosals, the earliest reported appearance of the vein of the cochlear aqueduct (the main venous drainage for the cochlear apparatus in therian mammals today). For reasons outlined in the discussion section, these osteological features are most likely associated with the elaboration of the macromechanical form of tuning, a unique cochlear functionality that provides the majority of modern therian mammals ultrasonic frequency sensitivity. However, because of the plesiomorphic dimensions of the cochlear canal seen in our specimens, the ability to detect mid-range to high frequencies was likely at most only incipiently developed in these stem therians. The osteological evidence provided here therefore suggests that many of the unique features of therian cochlear blood supply, histology and physiology evolved gradually after their split from the lineage leading to monotremes, and that these features originally functioned in service of highly sensitive and selective low-frequency hearing.

## Materials and methods

Anatomical images produced for this report were generated from manual segmentations of high resolution (~9 μm voxel size) CT stacks shot using a Nikon XTH 225 ST Scanner housed at Duke University’s Shared Materials Instrumentation Facility (SMIF). Labeled volumes of petrosal bones and their associated endocasts were converted into triangular mesh surfaces using the “Generate Surface” module in Amira. These surfaces were subsequently edited to contain 1–4 million topologically closed, manifold faces. Final images were rendered using these surfaces and the “cycles” shader in the free graphics program Blender.

In referring to *Priacodon fruitaensis*, the taxa represented by the Höövör petrosals, or other fossil taxa as stem therians, we are emphasizing their phylogenetic position as fossil taxa with more recent common ancestry to the extant clade Theria than any other living non-therian taxon (such as monotremes, and non-mammals). This matches the usage of the term “stem” seen in [5] and in phylogenetic discussions generally.

Osteological nomenclature used in the following descriptions of petrosal morphology are taken primarily from [20–22]. Terms specific to the description of labyrinthine endocasts are also taken from [23,24] for therian mammals; and [25] and [18] for non-mammals and monotremes, respectively. Nomenclature for cranial vasculature is taken from [12, 26–30].

The Aptian or Albian Höövör locality ([34] following spelling conventions of [35–36], but also transliterated Khoobur, Khobur, Khoboor, Khovboor, with varying diacritical marks) in Guchin-Us District, Mongolia has yielded an abundant sample of mammalian dental specimens (e.g. [37–42]) and three isolated petrosal specimens: an early stem therian termed “Höövör petrosal 1” (Fig 1c and 1d, PSS-MAE-104; [4]), a crown therian petrosal referred to the eutherian taxon *Prokennalestes trofimovi* (PSS-MAE-106; [37]), and an undescribed (but previously phylogenetically scored) specimen termed “Höövör petrosal 2” (Fig 1a and 1b, PSS-MAE-119) that is described below. Höövör petrosals 1 and 2 are referred to as Khoobur petrosals 1 and 2 in [4,5].

One of the most plesiomorphic mammals known from petrosal remains is *Priacodon fruitaensis* (Fig 1e and 1f; [5, 43]) from the Upper Jurassic (Kimmeridgian) microfossil locality at Fruita Paleontological Area, Mesa County, Colorado [44–45]. The specimen of *Priacodon fruitaensis* figured and described here (LACM 120451) is the left periotic region figured in [5], that was found in association with a fragmentary left exoccipital and other cranial elements. To facilitate the following discussion, the generic term *Priacodon* will refer specifically only to *Priacodon fruitaensis*. Also, the contractions H1 and H2 will be used to signify PSS-MAE-104 and PSS-MAE-119, respectively.

All permits for collecting and export of the Höövör specimens temporarily stored at the Collections of the American Museum of Natural History (AMNH), New York, were obtained as part of the on-going collaborative project between the AMNH and The Mongolian Academy of Sciences. The petrosals and associated remains of *Priacodon* are accessioned at the Los Angeles County Museum of Natural History.

As mentioned in [46], the description of isolated petrosal fossils from taxa insufficiently known from cranial remains often precludes the use of precise global anatomical directional terms. This report attempts to describe petrosal remains using a “local” directional system, whereby “anterior” or “rostral” is used to signify the authors’ best estimate of the direction corresponding to the anterior direction in a complete skull. In the petrosals described here, “anterior” therefore signifies the direction toward the lateral side of the tip of the petrosal promontorium furthest away from the fenestra ovalis, and toward the presumed location of the alisphenoid and entopterygoid bones. The term “posterior” or “caudal” is therefore used to refer to the opposite direction; whereas “medial” and “lateral” are used to refer to the directions perpendicular to the anteroposterior axis, toward the internal and external surface of the skull, respectively. Since the dorsoventral axis is much less ambiguous in these remains, no special definition is needed. These directional terms are chosen to reflect our presumption that the long axis of the promontorium is oriented approximately 45° towards the midsagittal plane, as seen in many Mesozoic mammalian petrosals that have been preserved in situ [27, 47].

Finally, novel abbreviations are used here for large-scale middle-ear character states seen in early mammals. These are “Detached Middle Ear” (DME), “Partially Detached Middle Ear” (PDME), and “Mandibular Middle Ear” (MME), referring to the adult condition of having a completely independent auditory apparatus and mandible, a primarily cartilaginous connection between the malleus and dentary, and a completely integrated jaw and ossicular chain, respectively. This terminology is meant to substitute for the less descriptive terms used by ([48–50], inter alios) that were defined with implicit taxonomic content. These terms: “Definitive Mammalian Middle Ear” (DMME), “Partial Mammalian Middle Ear” (PMME), Transitional Mammalian Middle Ear (TMME), and “Mandibular Middle ear of Cynodonts” (MMEC), are undesirable for the following discussion because of their assumed (and likely incorrect) application to members of the clades mentioned in their name. For instance, the term Definitive Mammalian Middle Ear suggests that this character state can be shown to be apomorphic for the crown mammalian common ancestor, and therefore unites the sister lineages leading to modern monotremes and therians; however, the DMME is neither definitive (under most hypotheses of the relationships of early mammals), nor is it diagnostic of Mammalia as a clade, making it not only incorrect but pernicious. The terms DME, PDME, and MME have under most current phylogenetic hypotheses of Mesozoic mammal relationships a congruent distribution (i.e they are extensively identical) as DMME, PMME, and MMEC (respectively), but are defined solely on observable anatomical features of the ear and jaw which will remain unaffected by changing tree topologies. This nomenclature allows the following text to more clearly differentiate the anatomical and phylogenetic composition of varying sets of synapsid taxa, especially in the discussion section and Supplementary Material 1 (S1 File).

## Results

### External anatomy of the second Höövör petrosal

**Fig 3:**
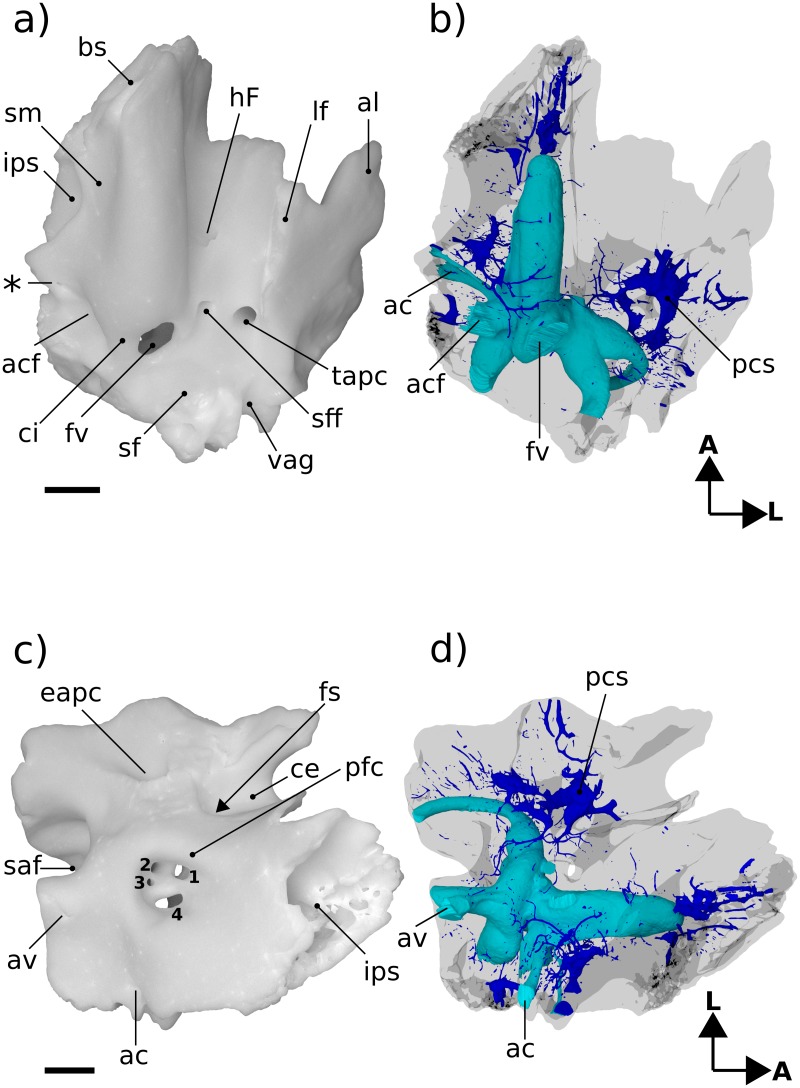
Renderings of Höövör 2 petrosal. a, b ventral view; c, d endocranial view. b, d showing partial labyrinthine endocast in teal and circumpromontorial venous plexus in blue. **A**, anterior; **L**, lateral. Numerical abbreviations: 1, primary facial foramen; 2, foramen for utriculoampullar branch of vestibular nerve; 3, foramen for sacculoampullar branch of vestibular nerve; 4, foramen for cochlear nerve. Scale bars are 1 mm. Asterisk marks location of small foramen which was interpreted by [4] as the tympanic aperture of the cochlear aqueduct. Refer to list of abbreviations in text for other abbreviations.

**Fig 4:**
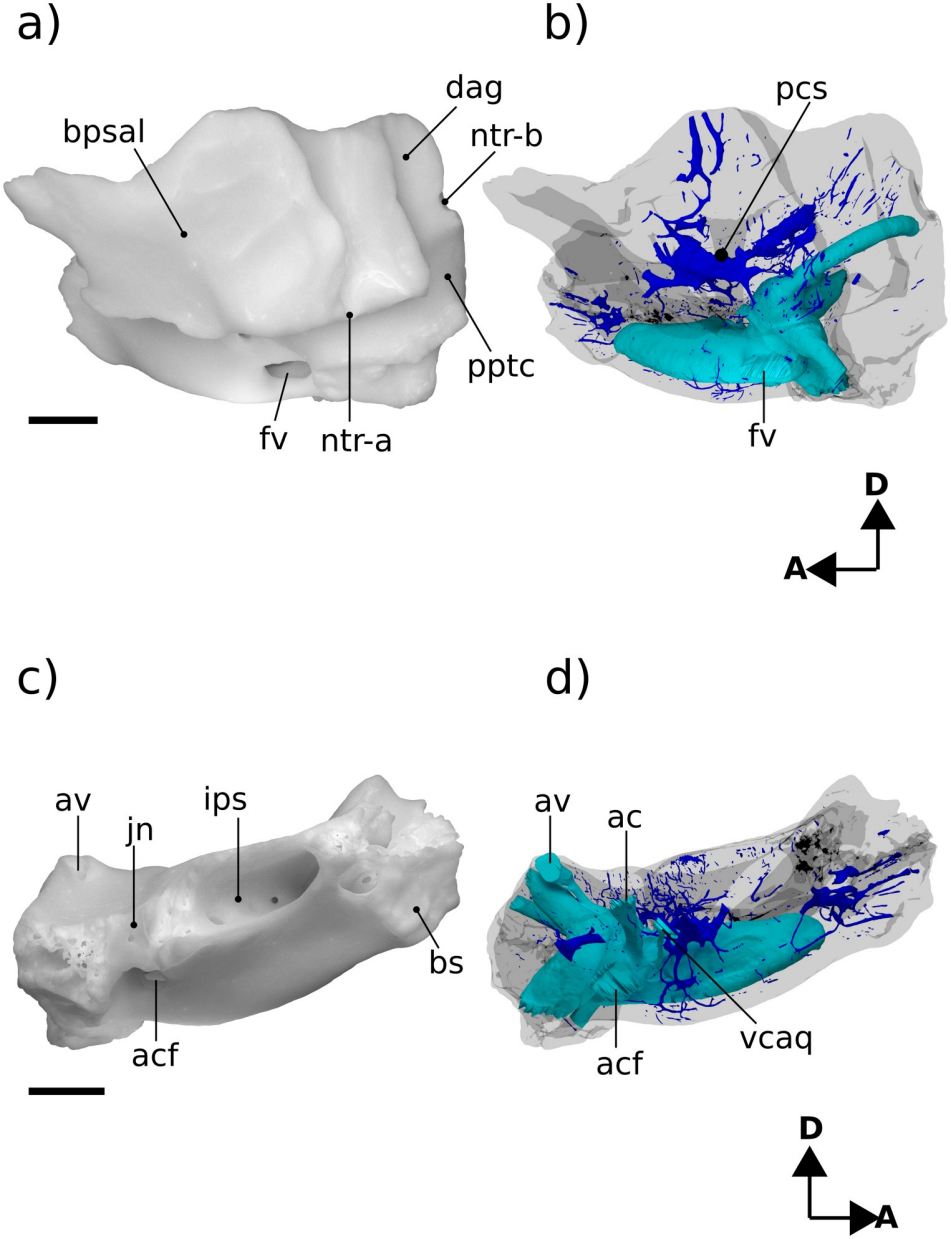
Renderings of Höövör 2 petrosal. a, b lateral view; c, d medial view. b, d showing partial labyrinthine endocast in teal and circumpromontorial venous plexus in blue. Scale bars are 1 mm. **A**, anterior; **D**, dorsal. Refer to list of abbreviations in text for other abbreviations.

The petrosal [21,27,51,52] can be envisaged as a generally tetrahedral structure with four major surfaces: tympanic (Fig 3a and 3b), cerebellar (Fig 3c and 3d), squamosal (Fig 4a and 4b) and mastoid. The petrosal itself is composed of two conceptual components, the pars canalicularis (containing the utricle and semicircular ducts) dorsoposteriorly, and the pars cochlearis (containing the saccule and cochlear duct) anteroventrally [12,53]. The tympanic surface is most apparent in ventral view and contains structures associated with the suspension of the ossicular chain, the fenestra cochleae, and foramina supporting the distribution of neurovascular structures. The topography of this region is defined mainly by the promontorium and lateral trough anteriorly, and an expanded post-promontorial region posteriorly. The cerebellar surface of the petrosal has several excavations, that accommodate central and peripheral nervous structures and vasculature. These include the internal acoustic meatus anteromedially, the subarcuate fossa (only the anterior half having been preserved in H2) posteromedially, a depression for the trigeminal ganglion (= semilunar/gasserian ganglion) anterolaterally, and the endocranial aperture of the prootic canal posteriolaterally running along the edge of the subarcuate fossa. The laterally facing squamosal surface, and the caudally facing mastoid surface (poorly preserved in H2) are both rugose because of their complex sutural interdigitations with surrounding bones (most probably the squamosal and exoccipital), and canals transmitting ramifications of the diploëtic and stapedial vessels. The anterior and medial margins of the petrosal are formed by exposed cancellous bone and the intramural inferior petrosal sinus which governs the topography here (Fig 4c and 4d).

Lost structures in H2 presumably include, the full extent of the tympanohyal, crista parotica, paroccipital process, and caudal tympanic process; the posterior half of the subarcuate fossa and impression of the sigmoid sinus; and most of the post-temporal canal accommodating the arteria diploëtica magna. However, several phylogenetically informative structures are better preserved in H2 than in H1; in particular, the anterior extent of the promontoruim and the anterior lamina-lateral flange.

#### Tympanic surface

The ventral expression of the pars cochlearis is the elongate and laterally steep promontorium (Fig 3a). Almost the full extent of this structure is preserved, and as such it can be determined that the anterior limit of the promontorium abuts into an anteromedially facing planar suture, most likely with the basisphenoid (“bs” in Fig 3a). The dorsal extent of this sutural plane is obscured by damage exposing underlying venous sinuses and cancellous bone. Posteriorly, the promontorium is terminated by the posterolaterally facing fenestra vestibuli. The 0.92 mm horizontal width of the fenestra vestibuli occupies almost the full extent of the posterior aspect of the promontorium, with the posteriorly directed crista interfenestralis projecting from the posteromedial corner of the promontorium as well. In both H1 and H2 the stapedial ratio [24] is approximately 1.2 (not above 1.5, as originally scored by [4,5]); and the anterolateral margin of the fenestra vestibuli is grooved to accommodate the footplate of the stapes.

The crista interfenestralis is straight and despite a minor posterior slant it follows the same general horizontal plane as the body of the promontorium. The posterodorsal root of the crista interfenestralis (along with all other structures within the posterior one third of the petrosal) is incompletely preserved; however, the surface curvature in this region suggests that the crista interfenestralis did not contact the posterior processes within the mastoid region, and that it blended subtly to lose distinction within the post-promontorial tympanic recess.

Between the anterior and posterior terminations of the promontorium there are no surface impressions of the promontorial artery, stapedial artery, or deep petrosal nerve (internal carotid nerve). The length of the promontorium is therefore shaped into a straight and smoothly rounded cylinder, with a sub triangular cross sectional profile. While the ventral edge of the promontorium is distinct (more so anteriorly) it does not show the level of salience seen in *Haldanodon* or *Megazostrodon* [5]. There is also no suggestion of a reduced rostral tympanic process (as in *Dasypus*; [22]) or impressions suggesting ventral contact of the promontorium with the lateral flange (as seen in multituberculates; [54]).

The lateral slope of the promontorium dips directly into the lateral trough, showing no distinct impression for the origin of the tensor tympani muscle anterior to the hiatus Fallopii (“hF” in Fig 3a), although the lateral trough is distinctly deeper in this region and it’s a likely position for the muscle. Along the anterior two thirds of the promontorium, its medial slope dips shallowly towards the concave medial margin of the petrosal bone. This area also contributes to the intramural enclosure of the inferior petrosal sinus dorsally. The medial aspect of the posterior one third of the promontorium curves directly into the ventral margin of the medially facing aperture of the cochlear fossula (“acf” in Fig 3a) [21,22,55]. The aperture of the cochlear fossula lies external to the fenestra cochleae and is fully separated from the perilymphatic canal (aqueductus cochleae) by a thin, horizontally oriented bony strut (the processus recessus; [56,57]); and is approximately 0.59 mm wide anteroposteriorly, and 0.75 mm high dorsoventrally. The space defined by the aperture of the cochlear fossula medially and the processus recessus dorsally leads anteromedially to a small anonymous venous canal (marked with an “*” in Fig 3a, and confluent with the inferior petrosal sinus and endocranial cavity) and to the jugular notch posteromedially (Fig 3a).

The bony perilymphatic canal in both H1 and H2 is approximately 1.3 mm in length, however H2 shows a greater degree of sophistication of the processus recessus structure because of the rounder, more ovoid, cross section of the perilymphatic canal it encloses. The perilymphatic canal in H1 in contrast is dorsoventrally flattened, and has much sharper anterior and posterior borders. Whether the morphology in H2 represents ontogenetic or phylogenetic development is uncertain, however, as variation in the compression of the cochlear aqueduct is commonly seen between individuals of *Dasypus novemcinctus* [22].

Because of its location posterior-dorsal-lateral to the pars cochlearis, the pars canalicularis and mastoid region is exposed ventrally as an “L”-shaped depression, dorsally offset from the promontorium. The perpendicular vertex of this “L” is posterolaterally offset approximately 1.5 mm from the center of the fenestra vestibuli. From this vertex, the mediolaterally oriented limb of the “L”-surface is damaged posteriorly, but the bases of four major topographic features are apparent. From lateral to medial these features are: 1) the ventral expression of the anterior lamina; 2) the open canal for the ventral ascending groove (“vag” in Fig 3a; for the proximal segment of the superior ramus of the stapedial artery); 3) the broken base of the paroccipital/mastoid region; and 4) the fossa for the stapedius muscle (“sf” Fig 3a) [32,58], placed directly posterior to the fenestra vestibuli. Major damage to the petrosal medial to the stapedius fossa precludes the recognition of other structures, such as the pocket medial to crista interfenestralis as seen in H1 [4].

The posteroventral extent of the anterior lamina is placed caudal to the line demarcating the periosteal surface of the anterior lamina anteriorly from its sutural surface with the squamosal posteriorly. The lateral portion of the horizontal limb of the “L” is therefore interpreted as marking the zone of synostosis of the embryonic lamina obturans with the endochondral bone forming the bulk of the petrosal. Compared to the shape of this region in H1, the vertical line demarcating the periosteal and sutural surfaces of the anterior lamina in H2 is much less laterally offset from the rest of the petrosal. As suggested in [4] the petrosal’s wide, solid, and laterally projecting sutural surface with the squamosal in H1 suggests that the glenoid fossa and other structures associated with the dentary-squamosal contact was relatively robust, a condition seen in many stem therians with large and transversely widened mandibular condyles.

Medial to the tympanic commencement of the ventral ascending groove (for the ramus superior of the stapedial artery) is the damaged base of the paroccipital/mastoid region. The anatomical landmarks commonly found in this region include a ventrally projecting paroccipital process, a rostrocaudally oriented crista parotica extending anterior to it, and possibly a caudal tympanic process of the petrosal running mediolaterally from the paroccipital process. All evidence of these features has been effaced from H2 due to the horizontal fracturing of their common base medial to the tympanic commencement of the ventral ascending groove. Therefore, while the presence and condition of the crista parotica, paroccipital process and most of caudal tympanic process of the petrosal cannot be commented on, the shape of this common base does demonstrate several significant contrasts with this region in H1. Most importantly, as mentioned in [4], the sutural surface of the squamosal bone’s contact with the petrosal in H1 extends onto the lateral margin of the crista parotica, and therefore would have formed the bulk of the fossa incudis (the depression accommodating the crus brevis of the incus). In contrast, the intact lateral margin of the broken base of the paroccipital/mastoid region in H2 is vertically steep and lacks a sutural surface with the squamosal. It is unclear what the phylogenetic polarity of these characteristics differentiating H1 and H2 would be; however, it is likely that these differences are a direct result of the more robust attachment of the squamosal to the lateral surface of the petrosal in H1, and the more ventral location of the ventral ascending groove (relative to the fenestra ovalis) in H2. The medial-most structure visible along the mediolateral limb of the “L” is the fossa for the stapedius muscle (“sf” in Fig 3a). The center of this depression in H2 is located further medially (closer to the crista interfenestralis) than in H1 (where it is located diametrically opposite the fenestra ovalis).

Anterior to the tympanic commencement of the ventral ascending groove (“vag” in Fig 3a), the anteroposteriorly oriented limb of the “L”- shaped exposure of the pars canalicularis is located between the lateral aspect of the promontorium medially, and the medial aspect of the lateral flange (ventral extension of the ossified lamina obturans; [27]) laterally. The tympanic surface of the anteroposteriorly oriented limb therefore forms an elongate and concave sulcus, termed the lateral trough [46]. The lateral trough is an anatomical crossroads for several important neurovascular structures (described below), and is perforated by three major foramina. Between the anterior margin of the fenestra vestibuli and the posterior base of the lateral flange, two of these foramina are aligned mediolaterally at the posterior end of the lateral trough. The lateral of the two is the tympanic aperture of the prootic canal (for the prootic vein = middle cerebral vein; [28]). The tympanic aperture of the prootic canal does not approximate or become confluent with the ventral commencement of the canal for the superior ramus of the stapedial artery, as is seen in *Ornithorhynchus* and many multituberculates [54,59]. There is also a small, anonymous, vascular foramen placed anterolateral to the tympanic aperture of the prootic canal, that communicates with the circumpromontorial venous plexus, and has a small sulcus leading into it (not visible in figures).

Medial to the tympanic aperture of the prootic canal is the more posteriorly directed secondary facial foramen for the entrance of the hyomandibular branch of the facial nerve into the cavum tympani. Compared to H1 and the spalacotheroid *Zhangheotherium* [47] the tympanic aperture of the prootic canal and the secondary facial foramen in H1 appear much closer. The location of these foramina are however ambiguous in the zhangheotheriid *Maotherium* [60]. In H1 the center of the secondary facial foramen is located anteromedial to the prootic aperture.

Anterior to the secondary facial foramen, the anteriorly oriented hiatus Fallopii perforates the lateral trough near the lateral margin of the promontorium. The hiatus Fallopii admits the palatine branch of the facial nerve (= greater petrosal nerve) in to the lateral trough. Therefore, the (approximately 1.28 mm long) lamina of bone extending between the secondary facial foramen and the hiatus Fallopii represents the bony floor of the cavum supracochleare (the space containing the geniculate ganglion of the facial nerve) and the inferior margin of the hiatus Fallopii. Anterior to the hiatus Falopii the lateral trough is more deeply excavated and roughened, similar to the condition seen in H1. This surface may represent a relatively indistinct area for the attachment of the tensor tympani muscle.

#### Lateral surface

**Fig 5:**
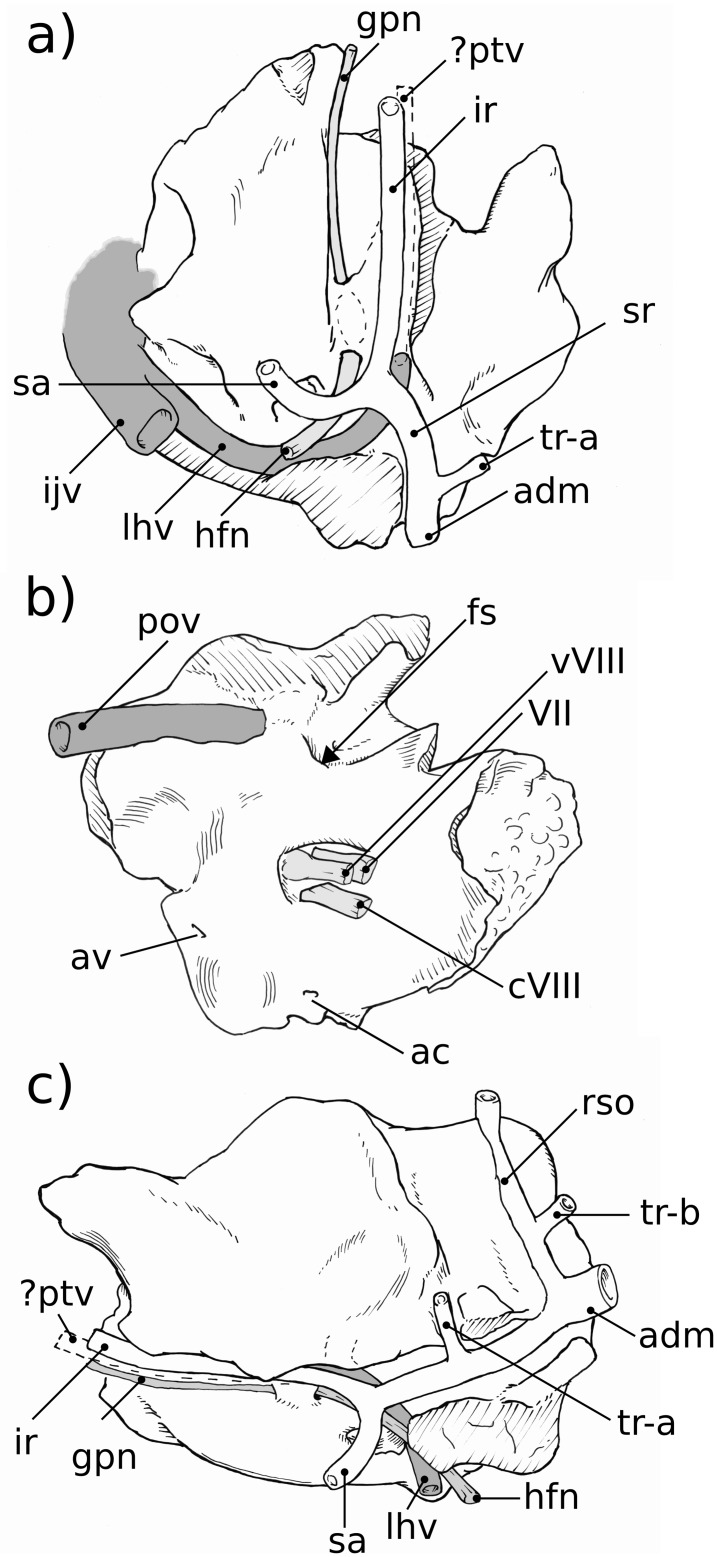
Neurovascular reconstructions of Höövör 2 petrosal. a, ventral view; b, medial view; c, lateral view. Venous structures shown in dark gray; nervous structures shown in light gray; the stapedial artery and its ramifications are unshaded. Refer to list of abbreviations in text for other abbreviations.

The preserved squamosal surface of H2 is composed mainly of the anterior lamina anteriorly, with some exposure of the lateral surface of the pars canalicularis posteriorly (Fig 4a). Four grooves are apparent on this surface, that represent the petrosal’s contribution to arterial canals enclosed laterally by the squamosal. Commencing near the posterior margin of the anterior lamina, posterior to the tympanic aperture of the prootic canal, the approximately 1.74 mm long groove for the superior ramus of the stapedial artery (ventral ascending groove) curves posterodorsally in a smooth arc. Almost halfway along its length, this canal gives off a distributary branch to a groove for a minor temporal ramus (of the superior ramus) of the stapedial artery. The posterior termination of the groove for the superior ramus of the stapedial artery is its point of confluence with the groove for the arteria diploëtica magna and groove for the ramus supraorbitalis of the stapedial artery (dorsal ascending groove; [27]). The large size (0.70 mm diameter) of the canal for the arteria diploëtica magna suggests that this vessel was the major supplier of arterial blood to the cranial connective tissues in this region ([5]; see Figs 3a, 5a and 5c). However, neither this groove nor the groove for the supraorbital ramus of the stapedial artery are preserved along their full extent or diameter, preventing the description of their precise distributions and possible ramifications. The visible extent of the groove of the supraorbital canal runs vertically along the lateral surface of the petrosal, directly lateral to the endocranial expression of the subarcuate fossa. A subsidiary groove for a possible second temporal ramus of the stapedial artery may be seen branching from the posterior wall of the dorsal ascending groove (for the supraorbital ramus of the stapedial artery). However, this feature may be the result of postmortem fragmentation and rounding.

Ascending laterally from the lateral flange there is an extensive anterior lamina. The ventral projection of the lateral flange is damaged; however, it is probable that not much of its original surface has been lost, and judging from the shape of the breakage it is improbable that it could have supported a sutural contact with a large quadrate ramus of the alisphenoid.

The lateral aspect of the anterior lamina does not show the mediolaterally projecting and horizontally flattened area (termed the anterolateral recess) that is seen in H1 [4]. Instead the lateral surface of the anterior lamina in H2 shows a steeper vertical slope, and terminates anteroventrally at the lateral opening of the cavum epiptericum (described below). The posterior extent of the anterior lamina contains a small vertical crest of bone lateral to the tympanic aperture of the prootic canal separating the periosteal and sutural surfaces of the periotic (“bpsal” in Fig 4a).

#### Cerebellar surface

The preserved cerebellar surface of H1 (Figs 1b and 3c) can be visualized as being composed of three endocranially exposed neural invaginations, anteriorly (the cavum epiptericum), medially (the internal acoustic meatus) and posteriorly (the subarcuate fossa) and a venous canal (the prootic canal) laterally; thus forming a “+” shaped pattern of topologically negative spaces in cerebellar view (Fig 3c). The intervening elevated surface between these four structures therefore forms a matching “x” shaped pattern, the center of which being composed of a massive elevation of bone. Lateral to this “x” is a large fragment of the vertically oriented anterior lamina, and most of the area medial to the “x” is formed by the pars cochlearis enclosing the cochlear canal. The posteromedial border of the “x” however is formed by a thin sheet of bone near the jugular notch (i.e. the processus recessus, with some contribution from the petrosal bone proper). The endocranial aperture of the aqueductus cochleae (“ac” in Fig 3c; also termed the perilymphatic canal) can be seen at the anterior border of this sheet of bone.

The internal acoustic meatus in H1 is an approximately 1 mm deep invagination, terminated by four foramina distally (numbered “1–4” in Fig 3c). Because of its oblique angle of descent into the substance of the petrosal bone, the endocranial rim of the internal acoustic meatus is shaped differently in each of its four quadrants, providing a convenient pattern for the description of its contents.

The prefacial commissure (“pfc” in Fig 3c; also suprafacial commissure; [20,21]) is the name given to the portion of the anterior ossified wall of the internal acoustic meatus partially bounding the proximal bony conduit of the facial nerve (“1” in Fig 3c), and which is not first preformed developmentally in the chondrocranium [21]. In both H1 and H2 the prefacial commissure extends posterolaterally from the shallower curvature of the posterior cranial fossa, and terminates laterally at the lateral-most point of the proximal aperture of the internal acoustic meatus. The entire 90° arc of the anterolateral quadrant of the internal acoustic meatus in H2 can therefore be thought of as consisting of the prefacial comissure, which extends distally (beyond the internal acoustic meatus) to form the anterior wall of the approximately 0.5 mm diameter primary facial foramen (“1” in Fig 3c).

Directly lateral to, and contiguous with, the prefacial commissure the endocranial surface (outside the internal acoustic meatus) shows a blunt, anteroposteriorly oriented crista petrosal separating the bulk of the cerebellar surface of the petrosal from the petrosal’s contribution to the bony floor of the cavum epiptericum (accommodating the trigeminal ganglion = semilunar ganglion = Gasserian ganglion; [21,61]). Being the anterior attachment of the tentorium cerebelli in extant mammals, the location of the crista petrosa near the medial margin of the cavum epiptericum likely marks the point of dorsal enclosure of the trigeminal ganglion into the dural folds separating the middle and posterior cranial fossae in H2. In more plesiomorphic forms such as *Morganucodon* [32] and *Priacodon* [5], and in modern *Ornithorhynchus* [62], the prefacial comissure itself and the pila antotica form the medial margin of the cavum epiptericum. Specimens H1 and H2 show a much wider prefacial commissure. This morphology is likely an osteological byproduct of the mediolateral dilation of the endocranial space within the posterior cranial fossa.

The anteromedial quadrant of the internal acoustic meatus is a continuation of the wider curvature of the surrounding pars cochlearis, and so does not form a distinct lip for the meatus. However, in H1 and H2 the ventral surface of the internal acoustic meatus in this region contains a low ridge of bone running distally into its depths, the crista transversa [22]. This low ridge loses distinction before reaching the distal terminus of the meatus (and therefore should not be called a “falciform crest”), but forms a separation between the foramen for the cochlear nerve (“4” in Fig 3c; in the posteromedial quadrant) and the primary facial foramen (“1” in Fig 3c; in the anterolateral quadrant). The crista transversa is distinctly higher in H1 and longer in H2, but in neither does it reach the height or salience of the falciform crest seen in *Homo* [51] and other modern mammals. The foramen for the cochlear nerve is ovoid, approximately 0.74 mm long along the axis of the cochlear canal and 0.3 mm wide mediolaterally, and shows smooth margins with no development of a tractus foraminosus (or cribriform plate). Dorsal to the foramen for the cochlear nerve, the distal surface of the internal acoustic meatus contains the two circular foramina for the branches of the vestibular nerve (“2–3” in Fig 3c). Based on the orientation and location of these two foramina for the vestibular nerve it is likely that they are homologous to the foramina for the utriculoampullar (“2” in Fig 3c) and sacculoampullar (“3” in Fig 3c) branches [18] seen in both extant therians and monotremes.

In both H1 and H2 the posterolateral and posteromedial quadrants of the internal acoustic meatus are formed by the raised wall of bone partitioning the internal acoustic meatus from the subarcuate fossa. The ventral floor of the internal acoustic meatus in this region contains the anteroposteriorly elongate foramen for the cochlear nerve medially, and the two smaller vestibular nerve foramina laterally or posterolaterally. These inferred homologies are supported by the trajectories of these foramina when viewed on the virtual endocast of the bony labyrinth; however, because these vestibular foramina do not form cylindrical canals leading directly to their peripheral targets, the precise targets of innervation of the nerves traversing the vestibular foramina cannot be determined conclusively.

Unlike the stem therians discussed here, fossil and extant cladotheres unanimously show the apomorphic distribution of peripheral axons within the vestibulocochlear nerve by the formation of osseous cribriform areas within preexisting foramina (the tractus foraminosus forming within the foramen transmitting the cochlear nerve being the most prominent example); although most of the contents of the foramen acusticum superius (area of the internal acoustic meatus dorsal to the crista transversa) remain free of trabeculated bony outgrowths in crown therians and all fossil cladotheres so far studied [7,8,63]. The foramen acousticum inferius on the other hand comprises the common depression for the foramen of the cochlear nerve and the foramina for nerves targeting the saccular macula (penetrating through the macula cribrosa media; [64]) and ampulla of the posterior semicircular canal (through the foramen singulare). The contents of the foramen acusticum inferius commonly recruit cribriform bony structures to secondarily infill these ancestral foramina in cladotheres. Additionally, because of its incorporation of a branch of the vestibular nerve (the sacculoampullar branch, or inferior vestibular nerve in *Homo*) along with the cochlear nerve, the foramen acusticum inferius is typically at least twice as large in areal extent than the foramen acusticum superius in Cladotheria. This trait has been mentioned by [63] as showing the apomorphic condition of their cladotherian specimen relative to *Priacodon*. In contrast, the area likely homologous to the foramen acusticum inferius in *Priacodon*, H1 and H2 is subequal or smaller than the foramen acusticum superius, and completely lacking cribriform bony infilling.

Directly posterior to the internal acoustic meatus is a wall of bone that forms the medial margin of the subarcuate fossa, enclosing the primary common crus of the bony labyrinth, and supporting the endocranial aperture of the aquaductus vestibuli (“av” in Fig 3c; containing the membranous endolymphatic duct). The distal half of this medial wall has been lost in H2 due to postmortem fracturing; and features such as the impression of the sigmoid sinus and contact with the exoccipital bone cannot be confirmed. However, the preserved morphology of this wall provides a reliable estimate of the maximal diameter of the proximal entrance to the subarcuate fossa. The proximal aperture of the subarcuate fossa is the endocranial expression of the anterior semicircular canal, and its 1.86 mm diameter in H2 closely matches the same length measured in H1. However, in both specimens it is apparent that the distal extent of the subarcuate fossa has a significantly larger diameter because of its medial excavation of its medial wall, distal to the constriction formed by the anterior semicircular canal. The medial diversion of the subarcuate fossa projects into the loop of the posterior semicircular canal, similar to the way the proximal aperture of the subarcuate fossa itself projects into the the loop of the anterior semicircular canal. The state of preservation in H2 does not allow the maximal diameter of the distal subarcuate fossa to be confidently estimated.

**Fig 6:**
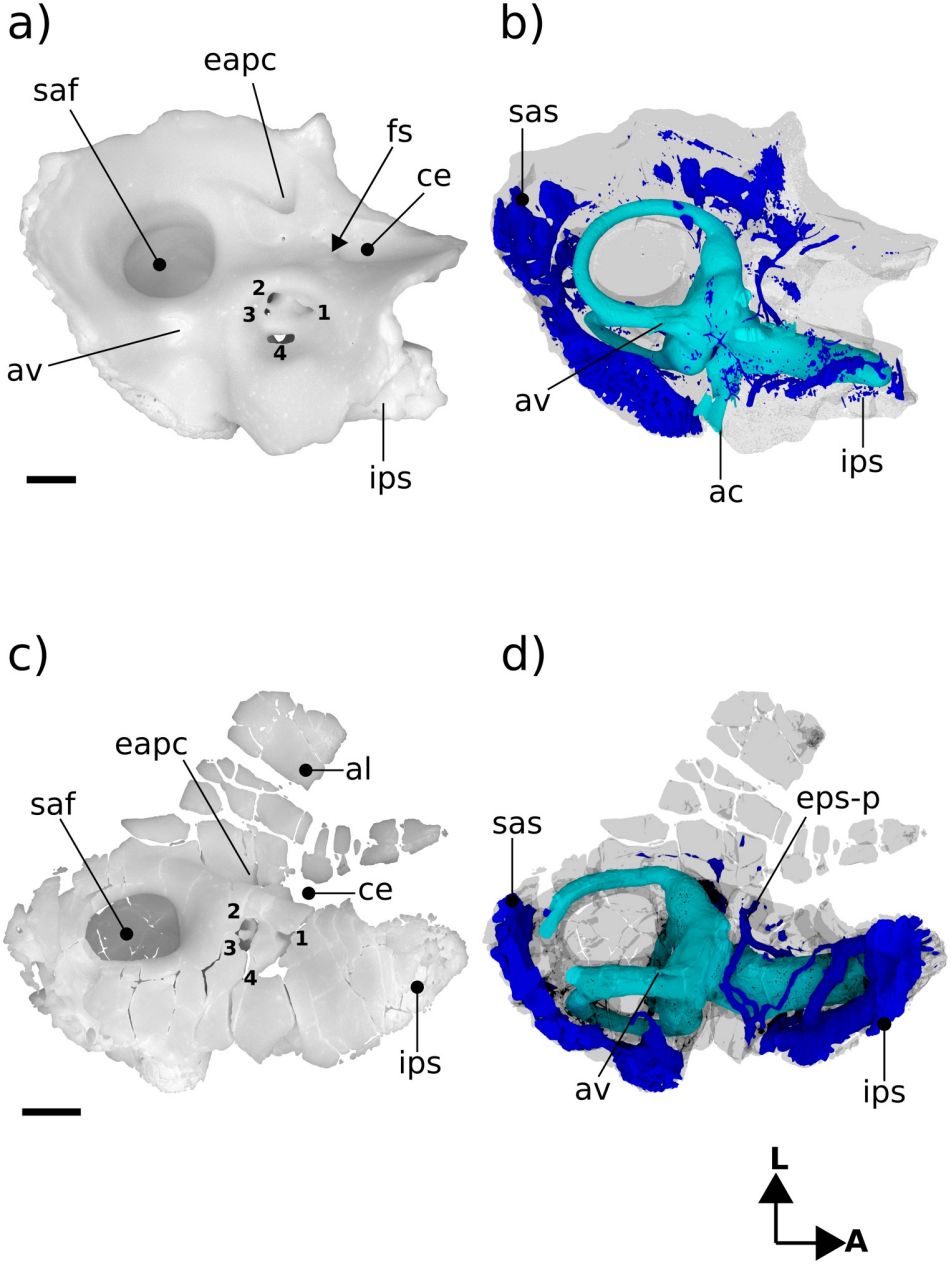
Renderings of stem therians in endocranial view. a, b Höövör petrosal 1 (right-to-left reflected); c, d *Priacodon*. Showing labyrinthine endocast in teal and circumpromontorial venous plexus in blue. Scale bars are 1 mm. **A**, anterior; **L**, lateral. Numerical abbreviations: 1, primary facial foramen; 2, foramen for utriculoampular branch of vestibular nerve; 3, foramen for sacculoampular branch of vestibular nerve; 4, foramen for cochlear nerve. Refer to list of abbreviations in text for other abbreviations.

Anterolateral to the subarcuate fossa, and medial to the posterior region of the anterior lamina, is the endocranial aperture of the prootic canal (“eapc” in Fig 3c). A groove leading into this aperture can be seen following the curvature of the anterolateral margin of the subarcuate fossa for a short distance before leaving the preserved edge of the petrosal. This structure is termed the groove for the middle cerebral vein in [32], and represents the approximate branching point of the transverse sinus from the middle cerebral vein. The enclosed prootic canal in H2 is substantially shorter (1.72 mm, versus 2.55 mm in H1) and less sigmoidal than the bony canal seen in H1. The diameter of the canal in both specimens is approximately 0.6mm along its entire length (Figs 3c and 6a).

The petrosal bone’s enclosure of the cavum epiptericum (“ce” in Fig 3c) is better preserved in H2 than in H1, allowing for a more precise characterization of this phyogenetically significant structure. As such, in H2 it can be determined that the impression for the cavum epiptericum on the petrosal is rectangular in general dimensions, with its posterior margin formed by an anteriorly facing border of bone placed anteromedial to the endocranial aperture of the prootic canal (this wall may represent the original anterolateral margin of the ossified otic capsule before its subsequent fusion with the ossified lamina obturans). Anterior to this posterior margin the bony floor of the cavum epiptericum extends approximately 1.64 mm anteriorly before terminating at the anterior margin of the petrosal. As mentioned above, the medial and lateral margins of the cavum epiptericum are formed by the crista petrosa and anterior lamina, respectively. These structures define the 1.19 mm width and 0.86 mm depth of the cavum epiptericum and contribute to two foramina communicating with this space. The posteriormedial corner of the cavum epiptericum contains the fenestra semilunaris (“fs” in Fig 3c) [27], that extends 0.5 mm medially within the crista petrosa to communicate with the cavum supracochleare. The anterolateral margin of the petrosal’s contribution to the cavum epiptericum is formed by an emargination of the broken rostral margin of the anterior lamina. This emargination would have comprised the majority, or entirety, of the margin of the foramen for the mandibular branch of the trigeminal nerve, and hence would be termed the foramen ovale (or the foramen pseudoovale [65]). The preserved posterior margin of the foramen ovale is an approximately 1.19 mm diameter semicircular indentation. However, preservation prevents the determination of whether this foramen is entirely contained within the anterior lamina, and what its total anteroposterior length would have been.

#### Neurovascular reconstruction

Fig 5 illustrates reconstructions of the major vascular ramifications on the external surface of the H2 petrosal. The vessels for which osteological correlates can be observed include tributary veins of the internal jugular circulation and distributary arteries of the stapedial/occipital circulation [66,67]. While not leaving a distinct impression on the fenestra vestibuli, the stapedial artery (“sa” in Fig 5a) likely ran laterally to a bifurcation point slightly posterior to the tympanic aperture of the prootic vein. Running posterior from this bifurcation the superior ramus of the stapedial artery occupies the ventral ascending groove, then forms an anastomosis with the wider arteria diplöetica magna, and also gives off a small temporal ramus near its anterior extent. Because of damage to the dorsolateral extent of the petrosal forming the dorsal ascending groove, the course of the ramus supraorbitalis (“rso” in Fig 5c) extending from the confluence of the arteria diplöetica magna and superior ramus of the stapedial artery cannot be reliably reconstructed. Likewise, the occipital artery, which likely contributed the majority of arterial blood to the cranial connective tissues through its confluence with the arteria diplöetica magna (“adm” in Fig 5c) and elsewhere, has no osteological correlate within the preserved morphology of H2 except for the grooves that represent its distributary branches.

The two main tributaries of the internal jugular vein are the lateral head vein (“lhv” in Fig 5a) and inferior petrosal sinus [28,59]. As will be discussed below both the prootic vein (“pov” in Fig 5a; also termed prootic sinus or middle cerebral vein in extant therians), before its confluence with the lateral head vein, and the inferior petrosal sinus receive minor venous tributaries along their course through the body of the petrosal bone. Because there is no anterior emargination of the tympanic aperture of the prootic canal (“tapc” in Fig 3a), H2 shows no osteological correlate of the post-trigeminal vein, leaving its existence in this taxon uncertain (it is therefore shown only with a dashed outline marked “?ptv” in Fig 5a and 5c). If, as in extant therians, H2 lacked a post-trigeminal vein the boundary between the prootic vein and lateral head vein would become completely arbitrary, and in this report it is taken to be at the point of emergence of the prootic vein onto the tympanic surface of the petrosal. A small venous foramen anterolateral to the tympanic aperture of the prootic sinus likely conducted a small tributary to the lateral head vein as well (not reconstructed in Fig 5).

In most respects the external vascular reconstruction of H2 is broadly similar to that provided by [4] for H1. The most salient contrasts being the relatively more ventral position of the ventral ascending groove (and therefore the superior ramus of the stapedial artery) relative to the foramen for the arteria diplöetica magna in H2 (Fig 5c). Given the uncertainty and variability in the presence and branching pattern seen in temporal rami (“tr-a” and “tr-b” in Fig 5c), the different reconstructed connectivity of the posterior temporal ramus in H1 (at the point of confluence of the arteria diplöetica magna and superior ramus of the stapedial artery) and H2 (from the ventral extent of the supraorbital ramus) should not be considered systematically significant.

### Comparison of labyrinthine endocast morphology between *Priacodon*, the Höövör petrosals, and extant mammals

#### Endocast preserved in pars cochlearis

**Fig 7:**
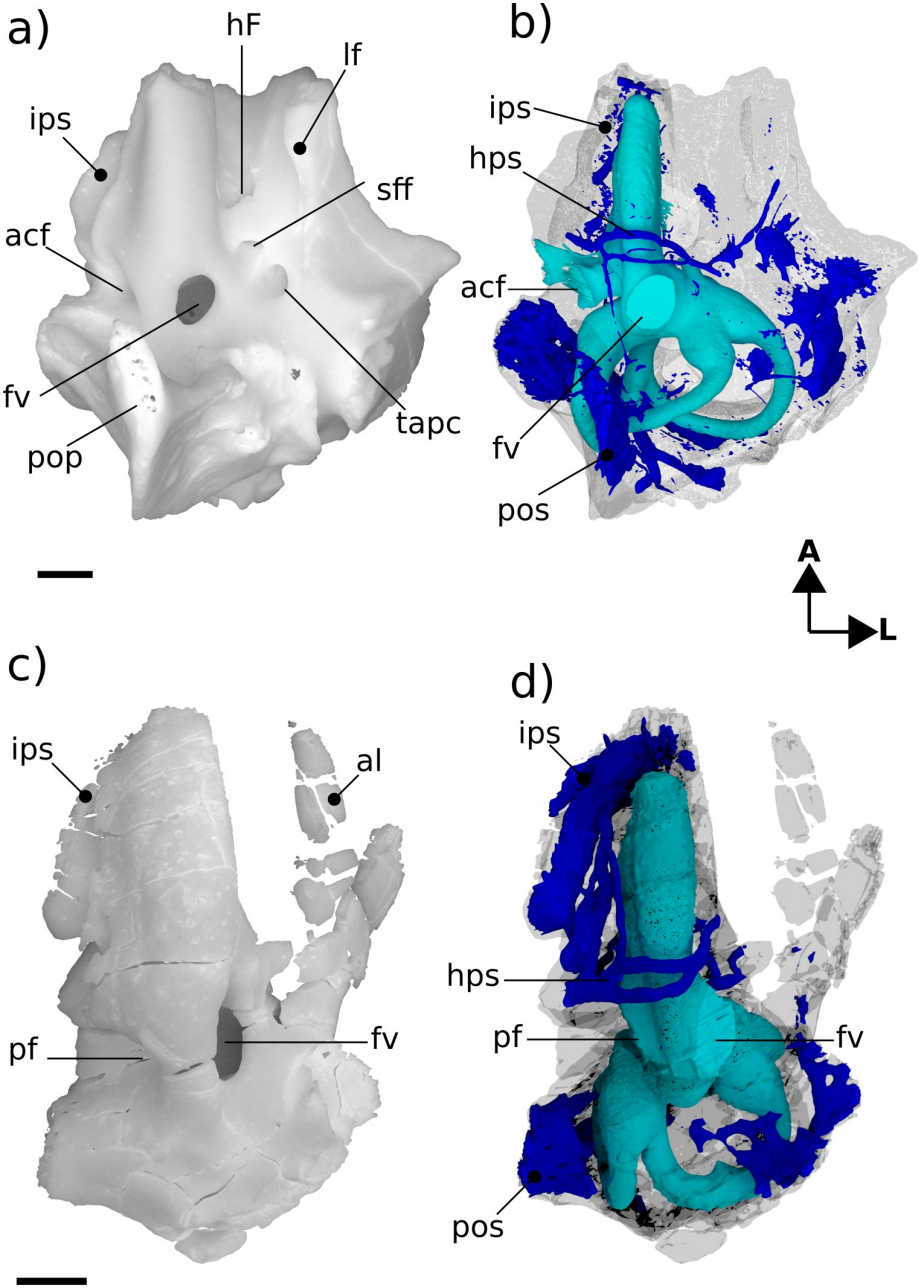
Renderings of stem therians in ventral view. a, b Höövör petrosal 1(right-to-left reflected); c, d *Priacodon*. Showing labyrinthine endocasts in teal and circumpromontorial venous plexuses in blue. Scale bars are 1 mm. **A**, anterior; **L**, lateral. Refer to list of abbreviations in text for other abbreviations.

**Fig 8:**
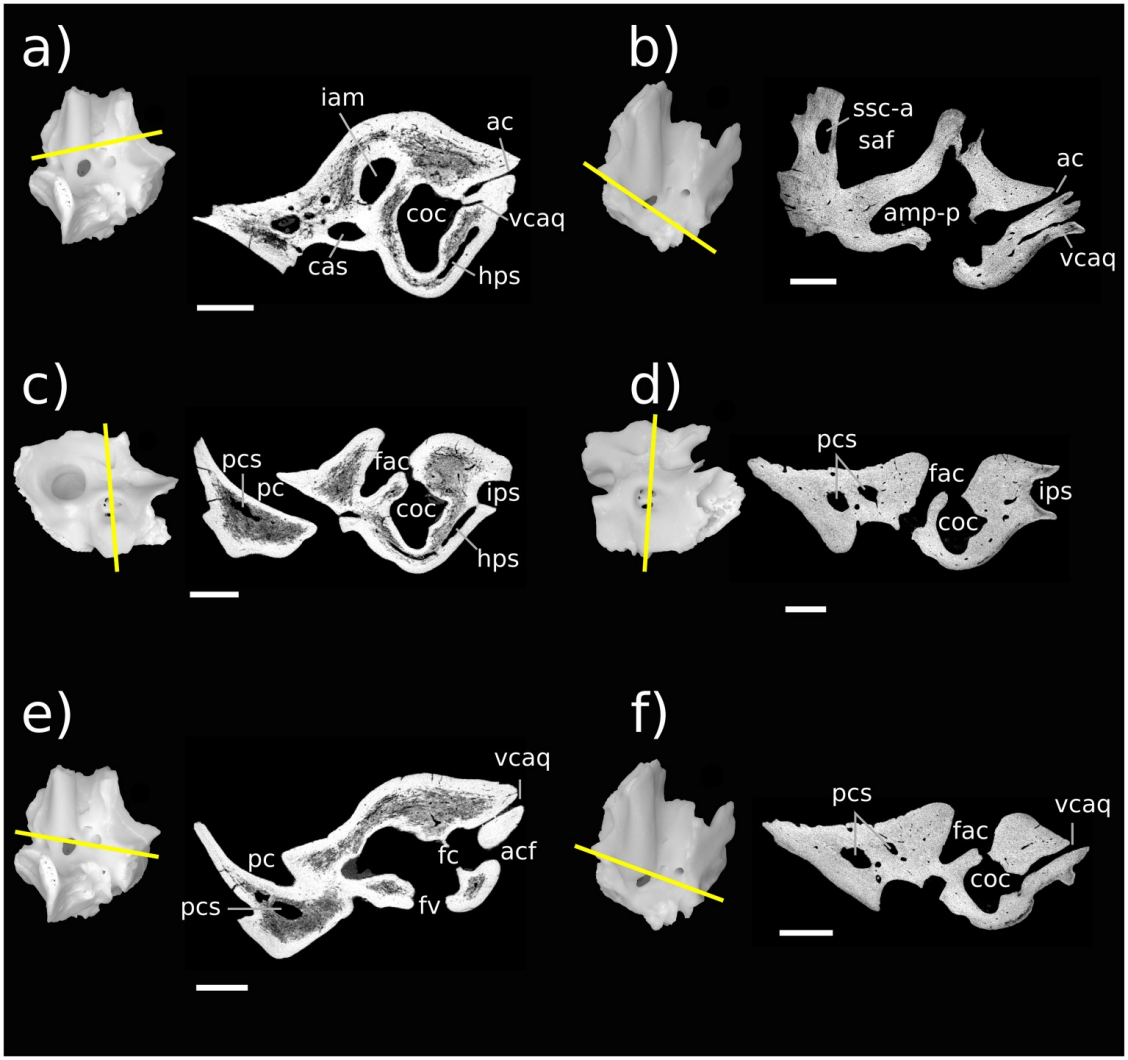
Resliced CT images of Höövör petrosals. a, c, e Höövör petrosal 1 (volume renderings are right-to-left reflected); b, d, f Höövör petrosal 2. a, b are oblique planes through both the cochlear aqueduct and canal for the vein of the cochlear aqueduct; c, d, e, f are coronal planes through the promontorium. Parts c, d are taken from a more posterior plane than e and f to show the hypocochlear sinus in H1. In all images left is lateral and dorsal is approximately toward the top of the page; all scale bars are 1 mm. Refer to list of abbreviations in text for other abbreviations.

**Fig 9:**
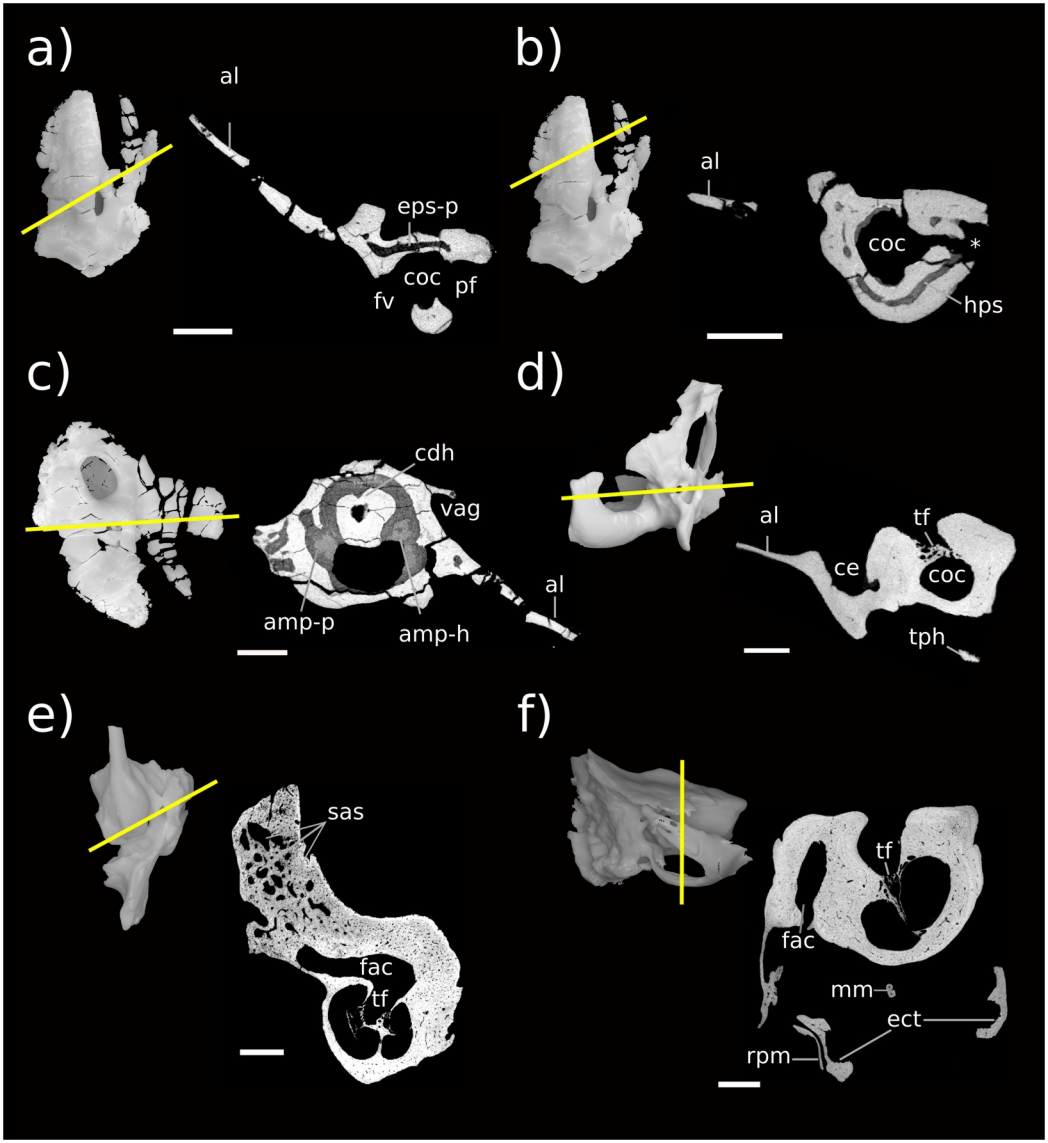
Resliced CT images showing *Priacodon* and several extant mammals. a, b, c images of *Priacodon*; d, coronal section through promontorium of *Ornithorhynchus*; e, coronal section through promontorium of *Didelphis*; f, coronal section through promontorium of *Dasypus*. a, b, are coronal sections through the promontorium of *Priacodon*, a is taken posterior to b to show the posterior epicochlear sinus; b, is taken rostral to the fenestra vestibuli and perilymphatic foramen to show the hypocochlear sinus. c, shows horizontal plane through the horizontal semicircular canal and its centripetal diverticulum. All scale bars are 1 mm; for 2D slices in a, b, d, e, and f lateral is toward the left of the page and dorsal is toward the top of the page; in c lateral is toward the right of the page and posterior is toward the top of the page. Asterisk shows location of damage to anterior wall of perilymphatic foramen in *Priacodon*. Refer to list of abbreviations in text for other abbreviations.

**Fig 10:**
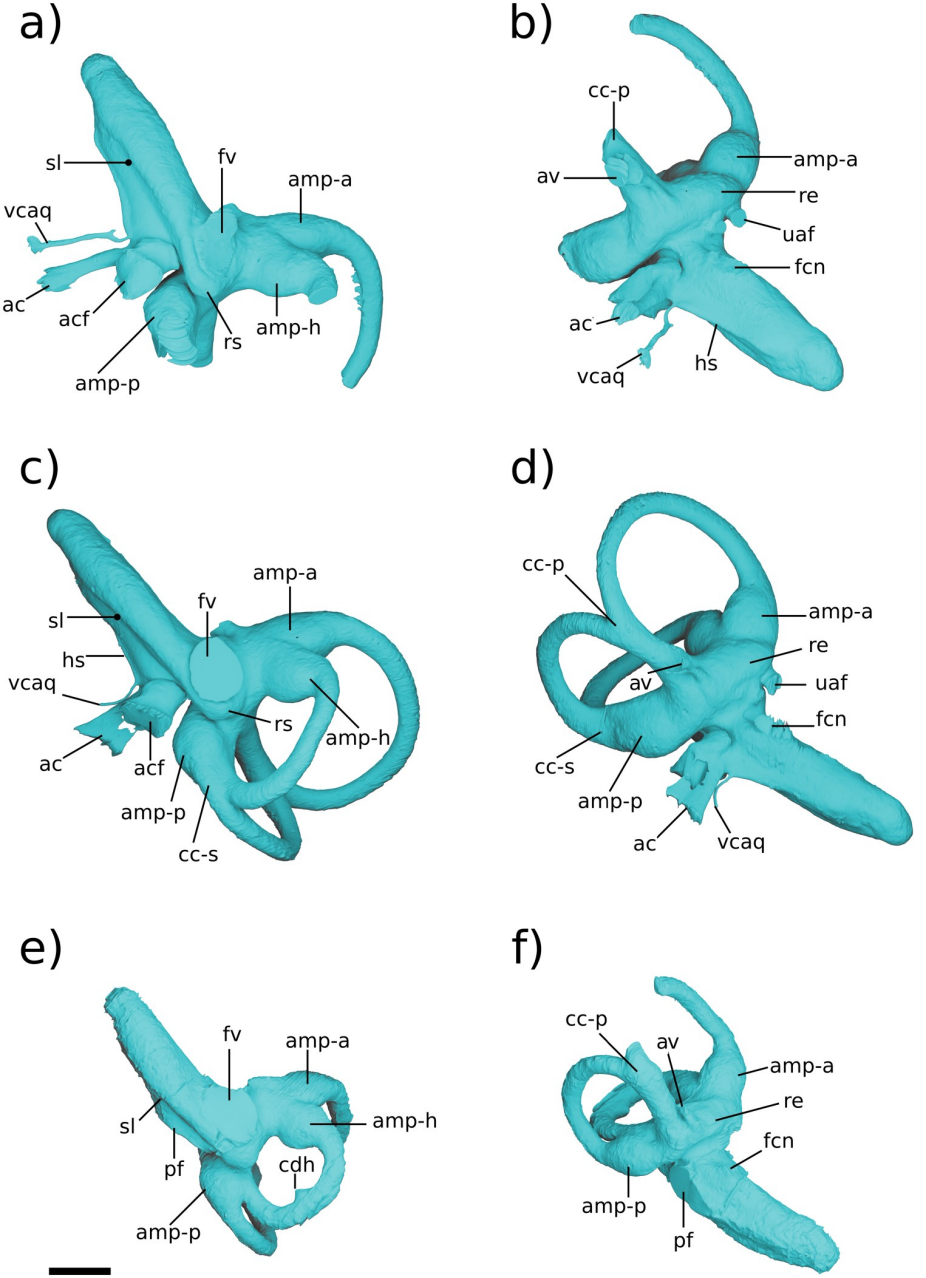
Renderings of labyrinthine endocasts in stem therians. a, b, Höövör petrosal 2; c, d Höövör petrosal 1 (right-to-left reflected); e, f *Priacodon*. All specimens shown as left-sided all scale bars are 1 mm. Refer to list of abbreviations in text for other abbreviations.

The straight distance along the bony cochlear canal, measured from the anterior surface of the recessus sphericus (the caudal apex of the saccular expansion; [24]) to the distal terminus (anterior apex) of the cochlear canal, is approximately 0.8 mm shorter in *Priacodon* than in either of the Höövör petrosals. However, the differences in length of the cochlear canal in all three of these specimens closely match the intra-specific variation seen in cochlear canal length reported in *Ornithorhynchus* (CL-cp; [18]), although *Ornithorhynchus* is much larger bodied than any of these stem therians. Also, despite being shorter, the shape of the cochlear canal in *Priacodon* can be still be interpreted as more derived than the cochleae seen in the Höövör petrosals because of its slightly stronger lateral curvature (see Figs 3–10).

While being a particularly homoplastic character, lateral curvature of the cochlear canal is seen only in mammaliaforms (especially the most plesiomorphic forms), with other amniotes developing medial curvature (i.e. convex towards the insertion of the cochlear nerve) to accommodate cochlear elongation. However, the incipient cochlear curvature in *Priacodon* only shows lateral deflection near its base, and no dorsoventral coiling (Fig 10e and 10f). Additionally, the fact that similar, or stronger, degrees of cochlear curvature are reported for mammaliaforms outside the mammalian crown group [68] presents the possibility (dependent on the precise phylogenetic interrelationships hypothesized for the taxa involved) that loss of lateral cochlear curvature may actually be an apomorphic feature of the taxa represented by the Höövör petrosals and later stem therians.

All three of the stem therian endocasts (Fig 10) show that the cochlear canal tapered somewhat towards its distal terminus (much more so in the Höövör petrosals than in *Priacodon*). In particular, none of these endocasts show prominent inflations or emarginations of the cochlear canal capable of accommodating a lagenar macula larger in diameter than the more proximal portions of the cochlear endocast. While the loss of its osteological correlate does not logically implicate the absence of a functional lagenar macula in these taxa [15], the morphology of the cochlear canal in these cases at least presents the possibility that these taxa had attained a terminal helicotrema (as in modern therians). This contrasts with the large, terminally positioned lagena and related nervous structures that are apparent in the osseus morphology of monotremes, several multituberculates, and mammaliaforms outside of the mammalian crown group [68,69]. Significantly, none of these stem therian endocasts show any of the “lagena related osteological characters”, as detailed by [70] for *Haldanodon*; such as a sulcus or canal for the lagenar branch of the cochlear nerve, a fossa for the lagenar macula, and/or canaliculi perforating a terminal portion of the cochlear canal for dendrites innervating the lagenar sensory epithelium. The relative restriction of the apex of the cochlear canal suggests the progressive reduction of the lagena within progressively more crownward members of the stem therian lineage. Conversely, the bony accommodation of lagenar function would therefore be a retained symplesiomorphy within allotherians and modern monotremes [2, 14–17].

All three stem therian endocasts also lack the “sunken” position of the fenestra ovalis (fenestra vestibuli) relative to the basal commencement of the cochlear canal, described by [63]. The sunken appearance of the fenestra ovalis was mentioned by these authors as being possibly apomorphic for the clade Cladotheria. However, given that this feature is also lacking in the South American cladothere *Vincelestes* [27], without a wider-scale phylogenetic analysis it seems at least equally plausible that an inset fenestra ovalis may be an apomorphic feature derived within dryolestoid cladotheres or some more exclusive group(s). A phylogenetic analysis informed by a large sample of cladotherian cochlear endocasts would be required to resolve the ancestral reconstruction of this feature.

One of the most marked features seen in the Höövör petrosals (Fig 8e and 8f) and not *Priacodon* (Fig 9a and 8b) and *Ornithorhynchus* (Fig 9d), is the complete segregation of the bony canal supporting the perilymphatic duct from the ossified aperture suspending the secondary tympanic membrane (the fenestra cochleae; “fc” in Fig 8e) and the aperture of the cochlear fossula (“acf” in Figs 3a, 7a and 8e). The secondary tympanic membrane is a thin epithelial bilayer found in many, if not most, amniotes [62], and segregates the fluid filled perilymphatic space from an air-filled intracranial space (such as a cavum tympani). The bony segregation of the secondary tympanic membrane from its confluent perilymphatic duct is, however, a characteristic seen only in advanced stem therians and variously in mature adult tachyglossids. The bony process of perichondral bone that completes the enclosure of the fenestra cochleae and aqueductus cochleae in the cohclear endocast of therians is termed the processus recessus [12,22,55–57].

The performance implications of this partitioning of the proximal scala tympani are uncertain ([17]: chapter 10). For instance, the processus recessus may prevent the unconstrained flux of perilymph from the scala tympani into the subarachnoid space. Whatever its functional advantage, it is very likely that the homoplastic distribution of the processus recessus in stem therians and in older adult tachyglossids is due to convergence [18,54]. Additionally, the incipient expression of an incomplete “processus recessus” has also been recognized in *Priacodon* [5], where it forms a linear ridge associated with the reconstructed course of the perilymphatic duct. Similar ridges described in multituberculates [54,59] also form a variable recessed or enclosed groove for the perilymphatic duct.

Otherwise, the presence of a well-developed processus recessus, fenestra cochleae, and aquaductus cochleae is known among members of the clade Trechnotheria (including the spalacotheres, dryolestoids, and therians; [36]), and in the derived “triconodont” clade Gobiconodontidae (GWR Pers Obs). These structures are retained in almost all known trechnotherian mammals (Sirenia, Elephantomorpha, and Eschrichtiidae being the main exceptions, due to atavistic reversal [69]). The tympanic aperture of the scala tympani, (whether this is the perilymphatic foramen as in *Priacodon*—“pf” in Figs 7c, 7d and 9a; or aperture of the cochlear fossula as in the Höövör petrosals) is of similar length, width, and perimeter in all three stem therians described here.

The impression of the scala tympani on the cochlear endocast (Fig 10) is confluent with the fenestra cochleae and aquaductus cochleae. It is visible in the stem therian endocasts as a medial inflation of the cochlear canal, delimited ventrally by the base of the bony secondary lamina (“sl” in Fig 10). The posterior margin of this space is further inflated as it meets the anterior margin of the cochlear fossula in the Höövör petrosals [56,57,62]. However, because of the lack of a tractus foraminosus, Rosenthal’s canal (spiral ganglion canal), or primary bony lamina in these taxa, the scala tympani does not leave recognizable anterior and dorsal boundaries on the cochlear endocast. The choice of interpretation as to the presence or absence of a vestigial lagenar macula also greatly influences the inferred placement of the scala tympani in the apical areas of the cochlear endocast.

In these relatively short straight cochlear endocasts, the impression of the scala tympani has the profile of a right triangle when viewed ventrally, with the anterior rim of the fenestra cochleae/perilymphatic foramen and the secondary bony lamina forming the triangle’s two perpendicular limbs. The hypotenuse of this triangle is formed by the medial contour of the cochlear endocast, which in the Höövör petrosals is also the location of a half-pipe shaped sulcus (i.e. a cylindrical prominence on the endocast; “hs” in Fig 10b and 10c), that originates immediately anterodorsal to the cochlear fossula. In *Priacodon*, damage to the rim of the perilymphatic foramen hinders the reconstruction of the endocast here (“*” in Fig 9b). However, it can be confirmed that there is no vascular sulcus along the medial margin of its cochlear canal (Fig 10e and 10f). Consequently, for *Priacodon* the medial hypotenuse of the triangle slopes anterolaterally more steeply than in the Höövör petrosals, causing the impression of the scala tympani to be limited to the proximal half of the cochlear canal (Fig 10e and 10f). In the Höövör petrosals the hypotenuse of the triangle has a much more gradual slope, causing the impression of the scala tympani to terminate more distally (approximately three quarters of the length along the cochlear canal). Distally, the impression of the scala tympani in the Höövör petrosals also shows a round secondary inflation, that interrupts the otherwise smooth lateral slant of the hypotenuse representing the medial contour of the cochlear canal. The half-pipe shaped medial sulcus is more distally extensive in H2 than H1, and can be followed along the complete length of the impression of the scala tympani. In H1 the medial sulcus loses distinction approximately half way along the length of the impression of the scala tympani, proximal to its slight terminal inflation.

**Fig 11:**
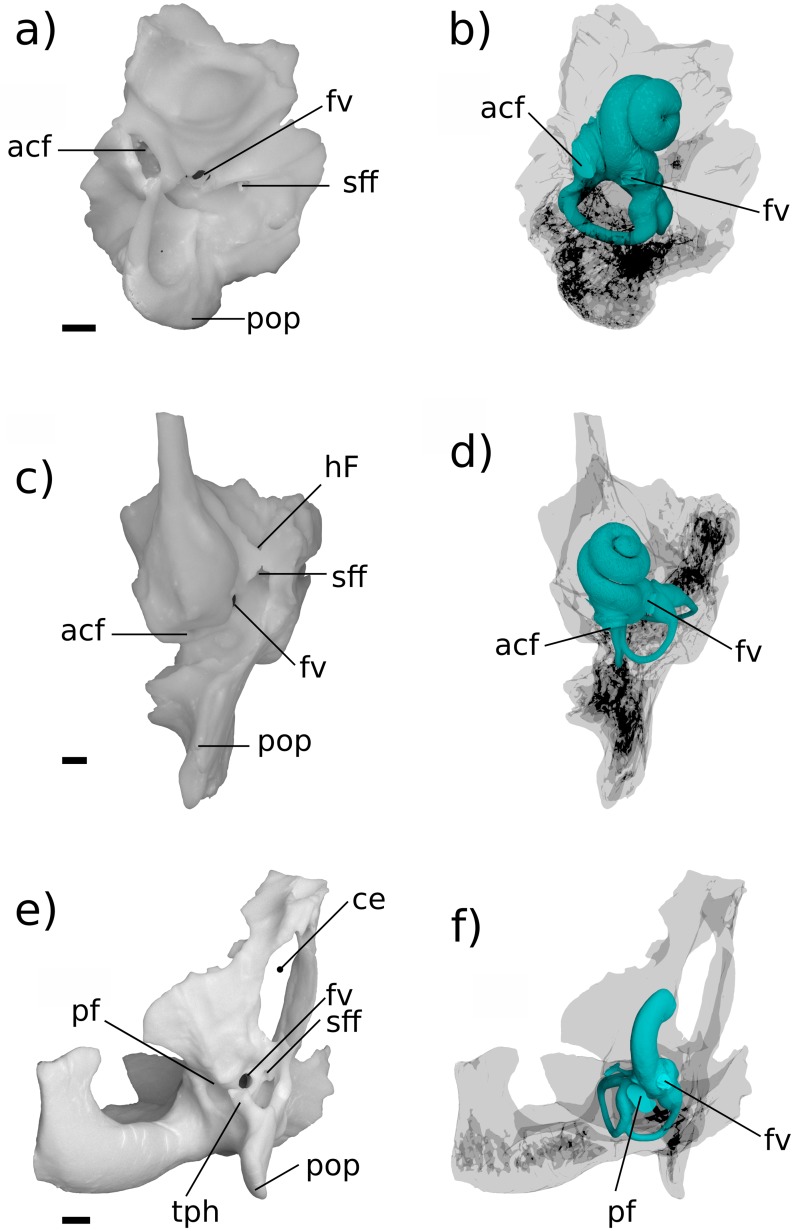
Ventral view of comparative mammalian specimens. a, b *Erinaceus*; c, d *Didelphis;* e, f *Ornithorhynchus*. All specimens are left-sided, venous sinuses are not shown. In e, f a fragment of the exoccipital bone is synostosed to petrosal. Medial is toward the left, rostral is toward the top of the page. All scale bars are 1 mm. Refer to list of abbreviations in text for other abbreviations.

**Fig 12:**
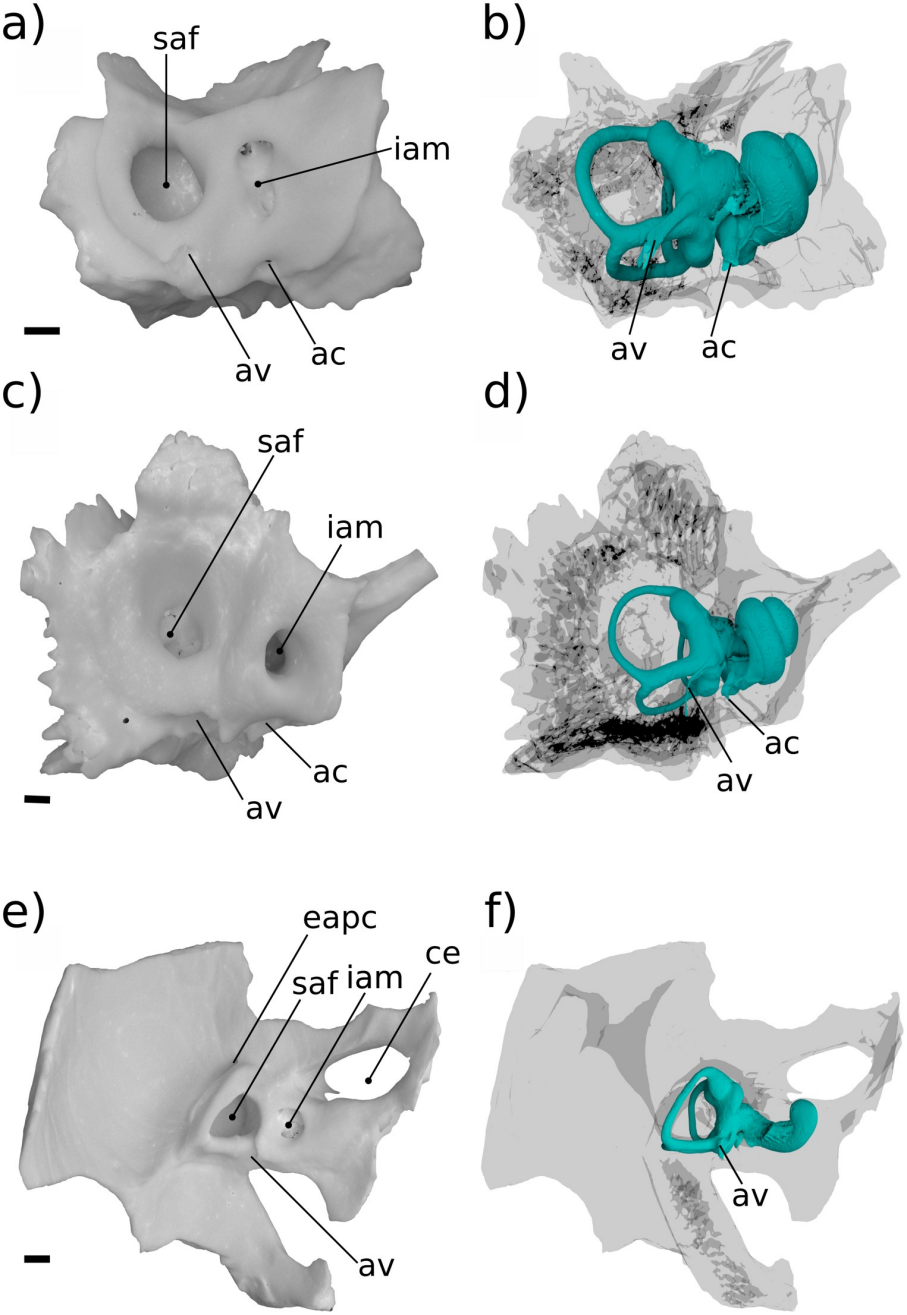
Medial view of comparative mammalian specimens. a, b *Erinaceus*; c, d *Didelphis*; e, f *Ornithorhynchus*. All specimens are left-sided, venous sinuses are not shown. In e, f a fragment of the exoccipital bone is synostosed to petrosal. Rostral is toward the right, dorsal is toward the top of the page. All scale bars are 1 mm. Refer to list of abbreviations in text for other abbreviations.

**Fig 13:**
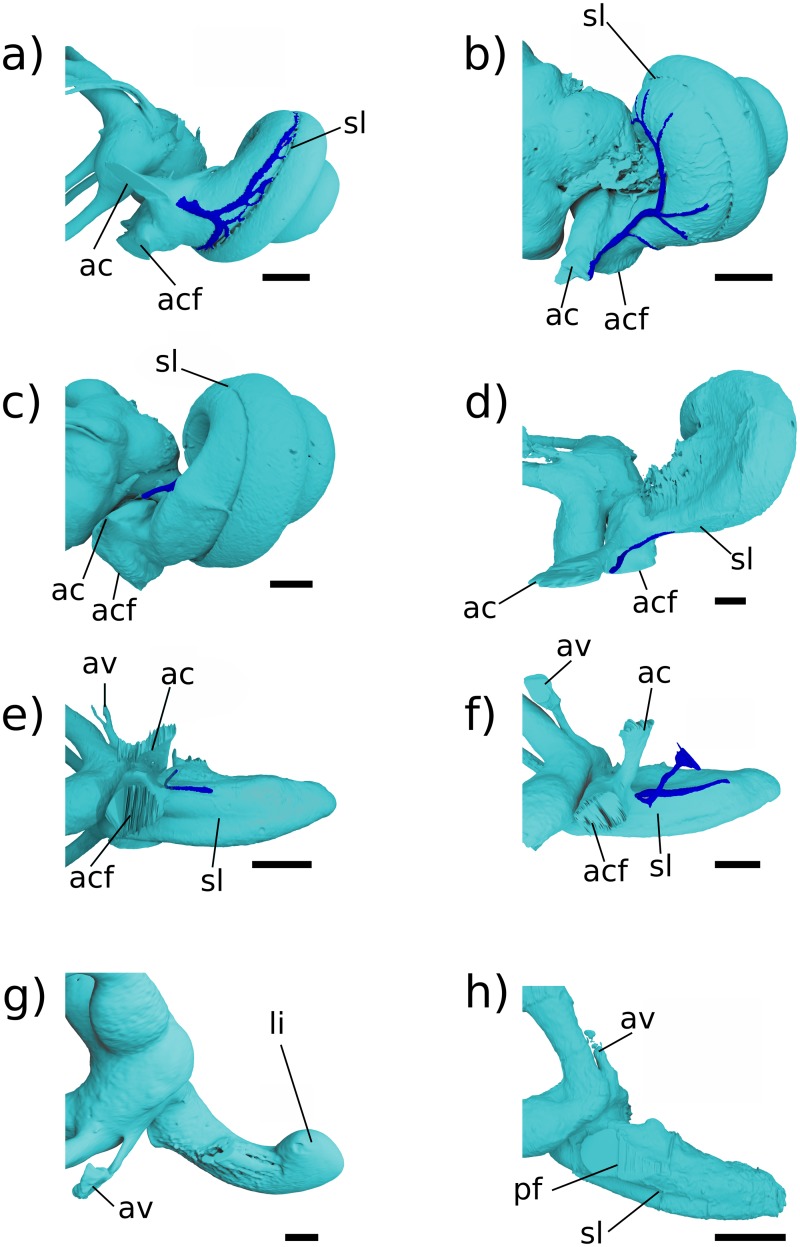
Medial view of cochlear endocast in crown mammals. All specimens are left-sided, vein of the cochlear aqueduct is shown in blue. a, *Dasypus*; b, *Erinaceus*; c, *Didelphis*; d, *Vincelestes*; e, Höövör petrosal 1; f, Höövör petrosal 2; g, *Ornithorhynchus*, h, *Priacodon*. All scale bars are 1 mm. Refer to list of abbreviations in text for other abbreviations.

In both Höövör petrosals (Fig 10a–10d) the proximal commencement of the half-pipe shaped medial sulcus within the impression of the scala tympani is confluent with the emergence of two tubular structures from the cochlear endocast. The anterior tubular structure is a small venous canal that, for reasons outlined below, is inferred to have contained the “Vein on the Cochlear Aqueduct” (“vcaq” in Figs 8a, 8b, 8e, 8f and 10a–10d; [71] also see [55]), and is therefore homologized with the bony “Canal of Cotugno” [72]. In some therians this canal follows a tortuous mediolateral trajectory to contact the inferior petrosal sinus (Figs 11–13; [71]) which is likely to represent the plesiomorphic condition for therians. In the Höövör petrosals, given the observed medial connectivity of this canal with a lateral diverticulum of the intramural inferior petrosal sinus, and its lateral continuity with the half-pipe shaped sulcus on the impression of the scala tympani (Fig 10a–10d), it is very likely that this medial sulcus transmitted venous components as well. In modern therians structures in this region represent the sole (or major; see [73]) outlet of venous blood from the pars cochlearis, and provide a subsidiary role in draining the pars canalicularis (Fig 13a, 13b and 13c). In the Höövör petrosals the half-pipe shaped medial sulcus on the impression of the scala tympani would then contain the homolog of what is the common cochlear vein (or one of its ramifications) in extant therian mammals.

Assuming this inference of homology is correct, the fact that the impressions of these venous structures are so prominently expressed on the endocasts of the Höövör petrosals documents a localized increase in venous drainage along the abneural border of the cochlea. This localized increase of the intracochlear venous systems runs opposite to the general mammaliaform trend of reduction of the intrapetrosal venous system [54,74]. A sizable cochlear vein may be a response to the increasing metabolic demands of the cochlear apparatus itself. In particular, this neomorphic drainage may be an evolutionary reaction to the increasing energetic and electrolyte requirements of the enlarging stria vascularis. As summarized in the discussion section, the stria vascularis is responsible for generating and maintaining the highly positive endolymphatic potential in modern therians [2,75–77]. In the extant mammals in which it has been studied it is also an organ with a complex development, recruiting epithelial and connective tissue contributions from cranial ectomesenchyme and other neural crest cell populations [78].

**Fig 14:**
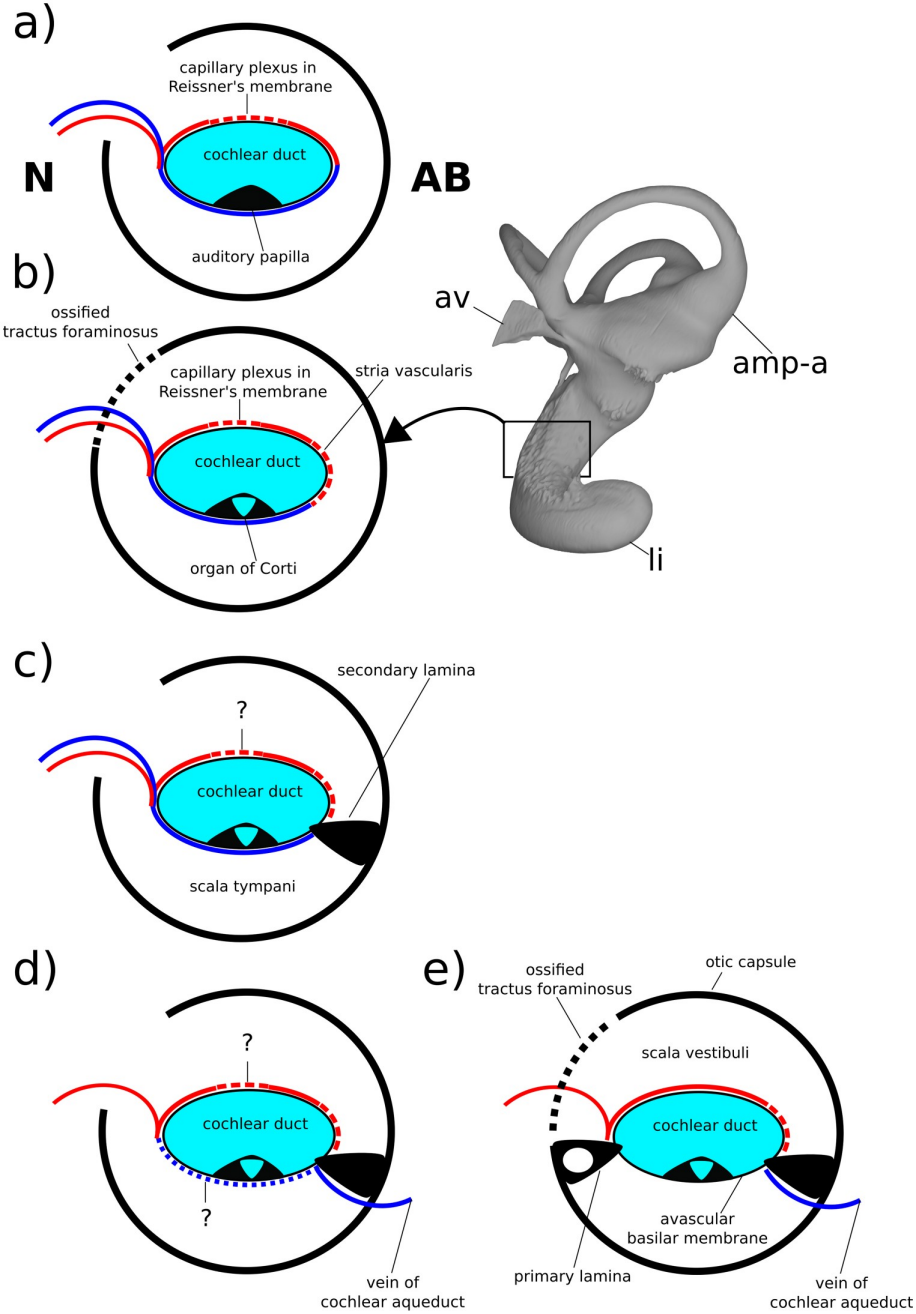
Schematic diagram showing hypothesized character states present in a cross section of the cochlear canal in early crown mammals and their fossil relatives. a, condition similar to that seen in sauropsid amniotes and hypothesized in eucynodonts; b, condition seen in modern monotremes; c, condition seen in *Priacodo*n and hypothesized in early stem therian mammals; d, more derived stem therian condition seen in the Höövör petrosals; e, condition seen in modern crown therians, e.g. *Homo*. At upper right is a medial view of the labyrinthine endocast of *Tachyglossus*, showing location of section diagrammed in b. **N**, neural; **AB**, abneural. Refer to list of abbreviations in text for other abbreviations.

Because the stria vascularis is a noted feature seen in all crown mammals including monotremes, the first appearance of bony correlates of venous structures servicing this organ in therian ancestors is likely a consequence of the closer impingement of the bony cochlear canal on to the stria vascularis and membranous cochlear duct generally (Fig 14). However, given the observations originally made by [79] of a probable endolymph-producing capillary plexus within the thickened Reissner’s membrane of *Ornithorhynchus* (the plesiomorphic condition for amniotes), the impressions of neomorphic veins draining the pars cochlearis in the Höövör petrosals may represent a wider evolutionary reorganization of the vascular pattern of the cochlear apparatus. The “modern therian” form of cochlear endolymph production, is accomplished solely through secretion by a highly active stria vascularis (Fig 14b–14e), with the arterial supply and venous return of blood servicing this organ incorporated into the walls of the scala vestibuli and scala tympani of the cochlear canal, respectively [29,30]. Conversely, the vascular branching within Reissner’s membrane seen in *Ornithorhynchus*, closely matches the position and morphology of a similar plexus in modern sauropsid amniotes [25]. In both monotremes and sauropsids the arteries and veins supplying the cochlear duct, Reissner’s membrane, and in monotremes the stria vascularis, are embedded within the membrane of the cochlear duct [25,79]. These intrinsic vessels of the cochlear duct enter and leave the bony cochlear canal through the foramen for the cochlear nerve. This contrasts with the separate bony foramina in the therian cochlear duct for the entrance of the labyrinthine artery and exit of the vein of the cochlear aqueduct. Where studied, non-mammalian amniotes also lack a strong endolymphatic potential and the stria vascularis; it is possible that this is similar to the condition in monotremes, but the endolymphatic potential in these mammals is still unknown [80–84].

The appearance of a clear intersection of the circumpromontorial venous plexus with the endocast of the cochlear canal in the stem therian lineage (Fig 10a–10d), and the localized enlargement of venous structures solely along the abneural side of the cochlear canal (the location of the stria vascularis in modern therians; Figs 13 and 14) in fossils otherwise showing a reduced proliferation of circumpromontorial venous sinuses, suggests that the Höövör petrosals supported a cochlear apparatus that functioned more like those found in modern therians than modern monotremes or any other vertebrate taxon.

Many modern therian groups contain a prominent sulcus for the inferior petrosal sinus near the petrosal-basioccipital suture (also see [26,85] regarding the interpretation of petrosal sulci in general). In most crown therians the sulcus for the inferior petrosal sinus (sulcus sini petrosi inferior; [52]) is smoothly concave and exposed endocranially, and is floored ventrally by a medially sheet-like flange of bone (crista promontorii medioventralis) which usually meets the basioccipital at a sutural contact. In taxa with an intracranial inferior petrosal sinus (such as *Canis* or *Homo*; [29,86]), its exposure to the posterior cranial fossa is facilitated by the fact that the bony crest flooring the sulcus for the inferior petrosal sinus is much larger than the weaker ridge of bone dorsolateral to it. However, in the Mesozoic stem therians described here, and all known stem mammaliaforms, the two flanges of bone ventrally and dorsally enclosing the inferior petrosal sinus are subequally developed, and both likely contributed to the sutural contact with the basioccipital medially [54]. Additionally, in these Mesozoic forms the space accommodating the inferior petrosal sinus itself is not a smoothly and consistently surfaced sulcus, but is an elongate confluence of a highly ramified network of venous sinuses that form a substantial proportion of the total volume of the pars cochlearis.

The extent of venous excavation within the pars cochlearis has been remarked on in many prior descriptions of the petrosal morphology in Mesozoic mammalian and advanced cynodont fossils ([27,70,87,88] inter alios); and this anastomotic network together with the intramural inferior petrosal sinus has been conceptualized broadly as the circum-promontorial sinus plexus by [32]. However, only with the recent availability of high resolution micro-CT imaging (particularly [31]) has the morphology and connectivity of this venous network been sufficiently characterized so as to allow for the comparison of its discrete and homologous structures. The present report corroborates the existence of the discrete tubular “trans-cochlear sinuses” originally described by [31] (“eps-p” and “hps” in Figs 6d, 7b, 7d, 8a, 8c, 9a and 9b), and demonstrates their phylogenetic distribution outside of the clade Docodonta. The general reduction of the venous versus sensorineural contributions to the overall volume of the promontorium in successively more nested clades within Mammaliaformes has also been remarked on by [88] among other sources. However, despite the relatively prolific extent of venous excavation of the pars cochlearis in stem mammaliaforms, these forms show few if any clear intersections of the circumpromontorial venous plexus with the endocast of the cochlear canal (e.g. Fig 7d). Because of the imperfect preservation of the stem therian petrosal sample used here, the precise ratio of venous to labyrinthine space within these specimens cannot be quantified; however, it is still apparent that the anatomical extent of venous proliferation in *Priacodon* is greater than that seen in either of the Höövör petrosals, matching phylogenetic expectations.

Despite having the greatest proliferation of venous structures in the pars cochlearis, the *Priacodon* specimen shows no intersection of the circumpromontorial venous plexus with the cochlear endocast (Figs 7d and 14c). This absence may ostensibly be an artifact of preservation because of the highly fractured nature of the specimen, especially along the medial surface of the petrosal where a structure such as the canal of Cotugno would be suspected (“*” in Fig 9b). However, since *Priacodon* shows consistently large-diameter distributary branches of the inferior petrosal sinus running tangentially to the cochlear canal near the perilymphatic foramen (Fig 9a and 9b), and lacks branches oriented radially towards the cochlear canal or a half-pipe shaped groove for venous structures on the cochlear endocast itself, it is reasonable to suspect that the venous reservoir within the petrosal of *Priacodon* had no direct confluence with the vessels servicing the interior of the cochlear canal. This pattern of connectivity is probably consistent with the lack of an additional drainage of venous blood seen in monotremes, suggesting that the veins draining the cochlea parallel the course of the labyrinthine artery and most likely empty endocranially into the basilar venous plexus (similar to sauropsids) [25,89].

**Fig 15:**
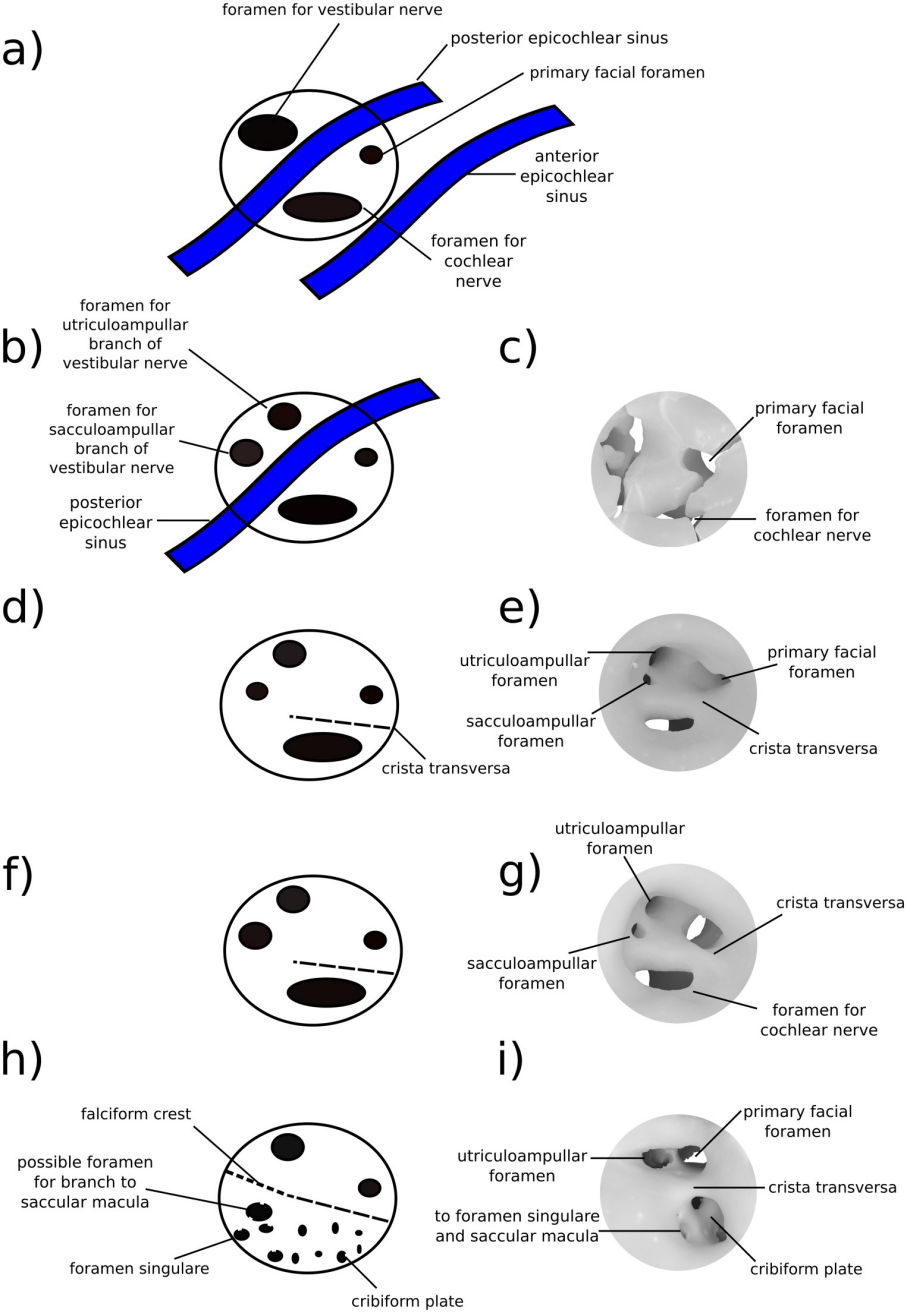
Schematic showing morphology of internal acoustic meatus in stem therians. Illustrations and renderings of left-sided internal acoustic meatus from endocranial view. Anterior is toward the right dorsal is toward top of page. a, condition in the docodont *Borealestes* and hypothetically all early mammaliaforms; b, c condition in *Priacodon* and hypothetically many early stem therians; d, e condition in Höövör petrosal 1; f, g condition in Höövör petrosal 2; h, i condition in extant therian mammals (e.g. *Erinaceus*). Character states in d-i are more morphologically derived than in *Priacodon*.

In *Priacodon*, as in the docodont *Borealestes* described by [31], a large proportion of the circumpromontorial sinus plexus is formed by tubular “trans-cochlear” sinuses (Fig 15; we suggest a different terminology for these structures below). In this report, we choose to refer to these venous structures running dorsal to the cochlear canal as epicochlear sinuses, to emphasize their dorsal position. These sinuses show no anatomical association with the contents of the cochlear canal and likely form venous anastomoses between several of the larger veins that leave the ventral braincase. In *Borealestes* (Fig 15a) the two epicochlear sinuses both run mediolaterally within the pars cochlearis dorsal to the cochlear canal, and are termed the anterior epicochlear sinus (“trans-cochlear sinus a” in [31]) and posterior epicochlear sinus (“trans-cochlear sinus p” in [31]). The anterior epicochlear sinus in *Borealestes* connects the inferior perosal sinus medially to a large venous foramen within the cavum supracochleare laterally. In [31] it is hypothesized that because of the enlarged secondary facial foramen in *Borealestes* a neomorphic continuation of the anterior epicochlear sinus may have left the ventral cranium with the hyomandibular branch of the facial nerve. In *Priacodon* (Fig 15b and 15c) there are several large tubular venous structures running laterally from their confluence with the inferior petrosal sinus. However because none of these structures could have provided a conduit for a vein connecting the inferior petrosal sinus to the cavum supracochleare (in addition to the generally small space available for the geniculate ganglion), it is likely that a venous structure homologous to the anterior epicochlear sinus (as seen in [31]) did not exist (Fig 6d). However, the posterior epicochlear sinus, which in *Borealestes* forms a confluence between the inferior petrosal sinus and the prootic sinus, does have a clear anatomical homologue in *Priacodon* based both on its orientation and connectivity. The posterior epicochlear sinus in both of these taxa traverses the pars cochlearis dorsal to the cochlear canal, within the bone flooring the incipient internal acoustic meatus (Fig 15a and 15b). Specifically, the posterior epicochlear sinus runs within the bar of bone separating the foramen (*Borealestes*) or foramina (*Priacodon*) transmitting the branches of the vestibular nerve (“2–3” in Fig 6c) from the other contents of the internal acoustic meatus (the primary facial foramen and foramen for the cochlear nerve; “1” and “4” in Fig 6c, respectively).

In *Priacodon* there is an additional “trans-cochlear sinus” running ventral to the cochlear canal, which is not seen in *Borealestes* (“hps” in Figs 7d and 9b). This sinus interconnects the inferior petrosal sinus with the same small aperture near the prootic canal as the posterior epicochlear sinus. While not present in *Borealestes*, this sinus is likely a plesiomorphic feature in many mammaliaforms given its presence in *Morganucodon* (as noted in an abstract by [90]) and in the first Höövör petrosal (Figs 7b, 8a and 8c), i.e. taxa both closer and more phylogenetically distant to crown therians than *Priacodon*. It is here termed the hypocochlear sinus (Figs 7b, 8a and 8c).

The hypocochlear sinus is the only “trans-cochlear” sinus shown by H1 (Figs 7b, 8a and 8c), and there is noticeably less venous proliferation within this specimen compared with *Priacodon*. However, despite the overall reduction of venous sinuses in H1, as mentioned above, the localized hypertrophy of a neomorphic vessel (within the bony canal of Cotugno) connecting the inferior petrosal sinus with the abneural cochlear wall forms an intersection of the circumpromontorial plexus with the cochlear endocast. This kind of intersection is not seen in *Priacodon* (Fig 7c and 7d) or any known stem mammaliaform taxa. Additionally, because of the longer extent of the prootic canal in H1 compared with *Priacodon*, there are a greater number of discrete venous branches draining into (or connecting with) the prootic canal than seen in *Priacodon*, some of which form minor contacts with the cavum supracochleare and the hypocochlear sinus. Neither H1 or H2 show epicochlear sinuses dorsal to the cochlear canal (Figs 3d and 6b); the close approximation of the primary facial foramen and foramina for the utriculoampullar and sacculoampullar branches of the vestibular nerve leaves no space available for these venous structures (Fig 15d–15g). Finally, H1 shows a possible smooth fracture connecting the medial border of the cavum supracochleare and laterobasal extent of the cochlear canal. Whether this fracture was facilitated by a pre-existing region of highly vascularized bone is uncertain, but seems likely.

In H2 circumpromontorial venous proliferation is much less extensive than in that seen in H1 (Figs 3 and 4 versus Figs 6b and 7b), and there is no consistently wide tubular connection between the inferior petrosal sinus and prootic sinus within the pars cochlearis (Figs 3b, 4b and 4d). This causes a greater degree of separation to exist between the venous sinuses on the medial and lateral borders of the petrosal; even though, with higher resolution micro-CT imaging, a large number of very small anastomotic connections between the prootic canal and inferior petrosal sinus do exist. As in H1, there is a discrete sinus surrounding the prootic canal, that sends out a number of small (presumably venous) interconnections to the cavum supracochleare and area around the fenestra semilunaris (Fig 3b and 3d). There is also a separate dorsoventrally oriented foramen located near the cochlear fossula and jugular notch, that has apertures on both the endocranial and tympanic sides of the petrosal (“*” in Fig 3a and visible in 4c). This small canal is also confluent with the inferior petrosal sinus anteriorly, and most likely transmitted a small anonymous vein or small nervous branch; however this foramen was incorrectly assumed to be the tympanic aperture of the perilymphatic canal in [5]. The posteromedial part of the cochlear fossula also shows several small (too small to be rendered in using the available micro-CT data) venous intersections with terminal tributary branches ventral to the cochlear canal. However, there are no “trans-cochlear” sinuses ([31]) anywhere within the pars cochlearis of H2, similar to the condition in modern therians and adult monotremes (e.g. Fig 9d–9f; [54]).

#### Endocast preserved in pars canalicularis

The portions of the labyrinthine endocast infilling the pars canalicularis in the stem therians reported here are broadly similar in morphology and most linear dimensions and angles (see Table 1; [91]). Of the morphological features differentiating these endocasts, especially those existing between the two Höövör petrosals, the majority are minor differences that do not rise above the level of what is commonly intraspecific variation. The remaining distinguishing features, generally support the more derived vestibular condition of the Höövör petrosals (Fig 10; [24]).

**Table 1:** Vestibule measurements of stem therian petrosals. All distance measurements are in millimeters, angle measurements are in degrees.

	Höövör 1	Höövör 2	*Priacodon*
Straight length of cochlear canal: from posterior saccule inflection behind fenestra vestibuli to tip of cochlea (mm)	4.357	4.38	3.66
Length of secondary bony lamina from saccular bulge behind fenestra vestibuli (mm)	2.57	2.69	NA
Anterior Semicircular Canal Height (ASCh SZ95)	2.48	NA	NA
Anterior Semicircular Canal Width (ASCw SZ95)	2.56	NA	NA
Length of primary common crus (mm)	1.95	NA	1.84
Posterior Semicircular Canal Height (PSCh SZ95)	1.73	NA	1.27
Posterior Semicircular Canal Width (PSCW SZ95)	1.62	NA	1.34
Horizontal Semicircular Canal Height (LSCh SZ95)	1.70	NA	1.12
Horizontal Semicircular Canal Length (LSCw SZ95)	1.79	NA	1.19
Length of impression of scala tympani: from front edge of the fenestra cochleae (or perilymphatic foramen) (mm)	2.15	1.96	NA
Angle Between Ant-Post SSC (degrees)	89.29	NA	100.47
Angle Between Ant-Hoz SSC (degrees)	73.30	NA	79.04
Angle Between Post-Hoz SSC (degrees)	95.26	NA	95.38

SZ95 –Measurement from [91]

This is apparent from the thinner diameter and more exaggerated loop of the semicircular canals, especially the anterior semicircular canal, which are both longer and wider in the first Höövör petrosal than in *Priacodon* (Fig 10c–10f). The anterior semicircular canal in the Höövör petrosals seem to be extended relative to the other canals by the endocranial invagination of the subarcuate fossa into the endocranial space circumscribed by the anterior semicircular canal (“saf” in Figs 3c, 6a and 6c). In H2, it is also apparent that the subarcuate fossa forms a medial diversion that also projects within the circumference of the posterior semicircular canal as well. Because of damage to the posterodorsal portion of the pars canalicularis in the second Höövör petrosal (Figs 3c, 10a and 10b), it is not certain if the configuration of the semicircular canals is even more elongate than in H1, however it is still apparent that H2 is much more similar to H1 than *Priacodon* (Fig 10e and 10f). Both Höövör specimens also show a relatively more inflated bony recessus ellipticus [92] for the utricular macula (“re” in Fig 10b, 10d and 10f). Conversely *Priacodon* shows greater similarity to H1 compared to H2, extant monotremes, and *Erinaceus*, by its greater projection of the posterior apex of the recessus sphericus (for the saccular macula) caudal to the posterior wall of vestibule (“rs” in Fig 10).

The canalicular endocast of H1 (and likely H2 as well) is unlike previously described Mesozoic dryolestoids [7,8,63] and more similar to *Priacodon* in that the arc of the horizontal semicircular canal is regularly circular, with their height and width being approximately equal. Strangely, the lateral (horizontal) semicircular canal in *Priacodon* is arguably apomophic compared to the condition seen in the Höövör specimens because of an anteriorly projecting (centripetally pointing) conical diverticulum in the horizontal semicircular canal placed within the inner contour of the canal (“cdh” in Figs 9c and 10e). Additionally, the lack of a secondary common crus (“cc-s” in Fig 10c and 10d; a feature likely to be plesiomorphic for mammaliaforms generally; [63]) in *Priacodon* may also represent a derived trait in this taxon.

The height of the primary common crus (“cc-p” in Fig 10b, 10d and 10f; the bony confluence of the anterior and posterior semicircular canals) is also similar between *Priacodon* and H1. The aqueductus vestibuli (“av” in Fig 10b, 10d and 10f; the bony accommodation for the endolymphatic duct) is similar in size between H2 and *Priacodon* (~0.1 mm in diameter), even though it appears relatively larger compared to other tubular structures present in *Priacodon*. The paravestibular canaliculus (the bony accommodation for the vein of the vestibular aqueduct, the main veinous drainage for the pars canalicularis, contributing blood to the sigmoid sinus in humans and probably most other amniotes; [29,30]) joins the aqueductus vestibuli along its proximal half in H1 and H2, but remains separate along its entire length in *Priacodon* where it directly intersects the dorsal surface of the vestibular endocast (Fig 10). While this is a salient difference visible in the high resolution images of stem therian endocasts used in this study, the polarity and distribution of this character are difficult to evaluate given the lack of sufficiently high resolution information for most Mesozoic petrosal specimens and the variable connectivity of the paravestibular canaliculus among extant mammals; i.e., the canaliculus can be seen to join the vestibular aqueduct proximally in *Ornithorhynchus*, *Tachyglossus*, and *Erinaceus*, but remains separate to the base of the vestibular labyrinth in *Dasypus and Didelphis*.

The angle formed by the intersection between the planes of the anterior and posterior semicircular canals is however significantly more plesiomorphic in *Priacodon* than in H1 (the only Höövör specimen for which this can be measured reliably). As reported by [92,93], a range of angles between 103°-157° between the planes of the anterior and posterior semicircular canals is typical of non-mammalian therapsids, and the ~100° angle measured in *Priacodon* is much closer to this range than it is to the range of values (~90° and smaller) typifying multituberculates and extant small mammals [92,93]. The ~89° angle seen in H1 is however within the range seen in extant therian mammals [92].

Two areas of the pars canalicularis in all three stem therians specimens show localized venous excavations (Figs 3, 4, 6 and 7). One of these sinuses, located in the posteroventral portion of the pars canalicularis and extending into the mastoid process, has been termed the paroccipital sinus by [31] (“pos” on Fig 7b and 7d). The greater extent of this structure in H1 than H2 is likely related to the better preservation in this specimen and the apomorphically large paroccipital process in this taxon (Fig 7b). A separate venous sinus arcs dorsal to the anterior semicircular canal, and is termed here the subarcuate sinus (“sas” in Figs 6b, 6d and 9e). We consider these regions of abundant, smooth-walled, interconnected cavities to be venous sinuses because 1) we do not see any confluence with the middle ear airspace whereby these sinuses could be pneumatized; and 2) even though these sinuses are only localized enlargements of the wider cancellous bony fabric of the interior petrosal structure, venous blood (as opposed to oxygenated arterial blood) is the major constituent of the spaces between bony trabeculae generally. As with other venous structures in the pars cochlearis, these venous sinuses are least developed (and may be missing altogether) in H2.

## Discussion

Estimating the neurosensory capabilities of any group of non-model organisms is necessarily more complex and error prone than research using humans or traditional animal models. These problems are compounded when analyzing fossil material, due to the obvious lack of relevant soft tissues and inability to work in an experimental paradigm. These complications do not decrease the unique value of fossil taxa in the study of mammalian nervous systems, however [94–96].

**Fig 16:**
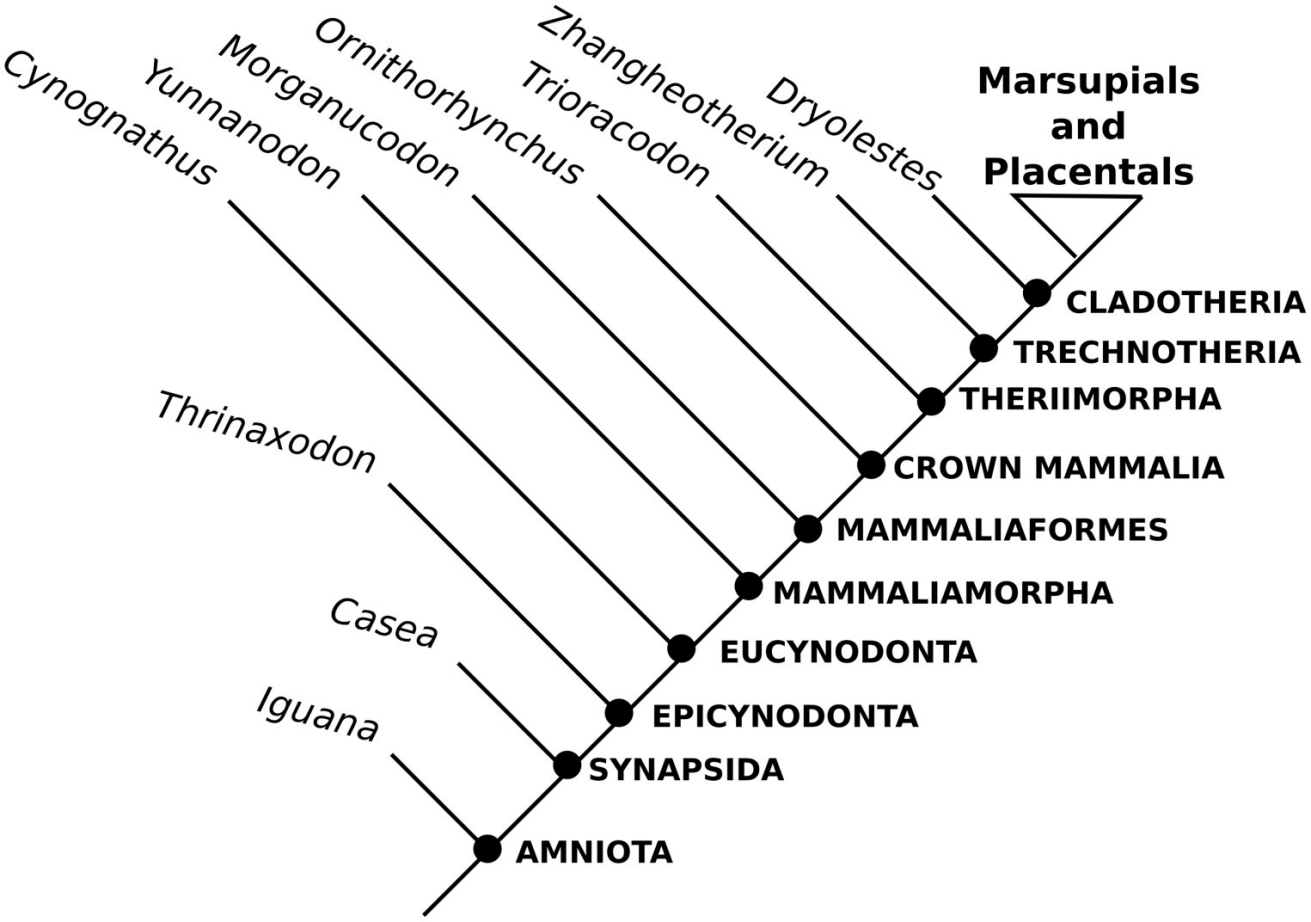
Cladogram showing consecutively nested clades referred to in discussion.

Because of the complexity and variety of evolutionary and neurobiological concepts involved, this discussion section begins with a summary of the auditory features of Mammalia and several consecutively nested subclades of therian relatives. Our hope is that this will provide a relevant paleontological, morphological, and neurobiological background to interpret the petrosals described above (see Fig 16). Supplementary Material 1 provides a longer review of prior research on the internal and external morphology of the periotic region in Mammalia and several more inclusive groups of terrestrial vertebrates, which may provide a useful introduction to the paleontological literature for those interested broader subjects in the evolution of mammalian hearing.

### Mammalia

The characterization of the crown mammalian ear solely from the morphology of extant taxa would provide a markedly distorted reconstruction of the mammalian common ancestor compared to what is known from modern paleontological evidence. This is because of the over 150 million years of independent transformation seen in both living mammalian lineages (monotremes and therians), making extant mammals an unrepresentative sample of mammalian diversity as a whole; and because of the homoplasy seen in the evolution of petrosal structures [36,68]. Based on the distribution of characteristics within fully adult extant mammals only, one could reasonably conclude that the condition of the crown mammalian common ancestor consisted of 1) a Detached Middle Ear (DME), with auditory ossicles fully independent from the lower jaw apparatus, and 2) an osseus armature for the distribution of the cochlear nerve axons termed the tractus foraminosus (“tf” in Fig 9d and 9f; creating the perforated cribriform plate within the internal acoustic meatus). However, recent fossil discoveries strongly suggest the convergent acquisition of these traits ([68,97], also see [98]).

The base of the secondary bony lamina is variably present in monotremes [18] and now seen in a more elongate state in *Priacodon* (Fig 10e), suggesting that some development of this structure may have been a derived feature of mammals in general (Figs 13g, 13h, 14b and 14c; although according to [18] in monotremes the base of the secondary lamina does not contact the basilar membrane). Of greater significance for the reconstruction of auditory capacities in early mammals are soft-tissue characteristics, many of which must be inferred based on their distribution in extant taxa. This heavy reliance on soft tissue for reconstructing past auditory ability highlights the importance of our models; i.e. it is difficult to predict in extinct forms functional or physiological traits that are not readily available in living representatives. These considerations suggest that the mammalian ancestor contained a secondary tympanic membrane (a membrane interface between the airspace of the cavum tympani and liquid-filled subarachnoid space, [62]), that was not at this stage completely suspended by a bony frame (such as the round window that formed in later mammals; “fc” in Fig 8e). As such the secondary tympanic membrane is a symplesiomorphy in crown mammals, and its suspension within a round window is a derived condition only within more nested members of this group. Several other soft-tissue symplesiomorphies in the mammalian ancestor are the retention of the lagenar macula and a membranous endolymph producing vascular plexus (both seen in Reissner’s membrane in monotremes today; [79]). These soft-tissue components of the cochlear duct were complemented by the presence of the stria vascularis, a specialized endolymph secreting organ (Fig 15b–15e), that developed within the synapsid lineage sometime before the divergence of the modern mammalian clades.

Most importantly, the mammalian common ancestor can be reliably inferred to have attained a true organ of Corti, the morphologically distinctive homolog of the amniote basilar papilla. The organ of Corti is diagnosed by the functional differentiation and separation of two subgroups of auditory hair cells along an axis perpendicular to the axis of tonotopy [17,99]. This division of labor between the less derived and more afferently innervated Inner Hair Cells (IHCs) on the neural side of the basilar membrane, and more specialized and efferently innervated Outer Hair Cells (OHCs) on the abneural side of the basilar membrane, is a fundamental component of macromechanical tuning [100,101], the characteristic tuning present in mammals. In therian mammals the functional specialization of OHCs to amplify weak (i.e. quiet) or dampened pressure waves propagating along the cochlear canal is implemented through the action of an apomorphic reverse transduction mechanism [2,101]. Possibly correlated with this adaptation is the lack of evidence for non-macromechanical forms of frequency resolution (the electrical or micromechanical forms of cochlear tuning; see Supplementary Material 1) in mammalian cochlear function ([14]; although for evidence of a possible minor role of a micromechanical active processes in some mammals see [102,103]).

The segregation of two sub-populations of auditory hair cells is delimited morphologically by a patent tunnel of Corti running longitudinally within the endorgan, and specialized populations of non-sensory supporting cells within the cochlear epithelium. Additionally, the mammalian common ancestor seems to have also defined the relative allometric size of the combined auditory hair cell population, with extant monotremes and similarly sized therian mammals (dogs, cats, etc.) having a similar total number of these cells [104]. However, the relatively much more elongate and organized organ of Corti seen in modern therians creates a thinner and longer distribution of the auditory hair cells, compared to monotremes.

It is unclear how innovative these soft tissue characteristics are to the mammalian clade itself, and the organ of Corti and stria vascularis in particular are likely to have appeared at an earlier point in synapsid phylogeny, within Mammaliaformes if not earlier. It is therefore also unclear if the petrosal of the earliest crown mammals would be diagnostically recognizable from the petrosals seen in other early mammaliaforms such as *Hadrocodium* and *Morganucodon* (see [105] for two examples of this frustrating ambiguity). Finally, it is also unknown if the uniquely mammalian external ear, with its characteristic pinna structure, would have appeared within the crown mammalian ancestor, within other advanced synapsid taxa, or at some later point solely within the stem therian lineage. The soft-tissue evidence for an involuted cartilaginous pinna in *Tachyglossus* reported by [106] is a possible homolog to the external structures seen in extant therians. Otherwise the phylogenetically deepest evidence for the presence of external pinnae is provided by *Spinolestes*, an excellently preserved gobiconodontid [107], and member of the diverse stem therian clade Theriimorpha.

### Theriimorphans

The monophyletic group containing the carnivorous eutriconodonts, the more derived “acute symmetrodonts,” and therian mammals–Theriimorpha–is the most inclusive well-supported group within the therian stem lineage [1,5,68,108]. Despite the relative abundance of excellently preserved fossil remains referable to early members of this group (such as *Spinolestes* mentioned above [107]), several authors have mentioned the relative lack of knowledge on the internal petrosal morphology in most members of this clade (e.g. [69,81]). However, the prior descriptions of external petrosal morphology in eutriconodonts [5,46], combined with the observations of the internal petrosal anatomy of *Priacodon* outlined above, suggest that basal theriimorphans can be characterized by a similar but slightly straighter morphology of the cochlear canal than the earliest mammaliaforms. This is reflected by the steeper lateral aspect of the promontorium in ventral view, and lack of an apparent apical inflation for the lagenar macula within the labyrinthine endocast (although in *Priacodon* the cochlear canal remains relatively untapered and thick to its apex).

The cochlear endocast of *Priacodon* (Fig 10e and 10f) also shows the first appearance of a elongate and projecting secondary lamina in early theriimorphs. In extant therians the secondary lamina is associated with tonotopic locations of the cochlea dedicated to hearing above 10 kHz [15,109]. However, at the time of its first definite appearance in the theriimorph taxa described here, its functional significance is much less clear. Given that the secondary lamina appears without an opposing primary bony lamina or even tractus foraminosus to provide an opposing attachment for the basilar membrane, it seems unlikely that significant tension would be able to be transmitted across the scala tympani side of the cochlear duct. Additionally, as the variably present “base of the secondary lamina” in extant monotremes has been observed to lack a direct connection (histological adherence) with the cochlear duct when present [18], it is conceivable that the (much longer and wider) secondary lamina seen in *Priacodon* and the Höövör petrosals similarly lacked an association with the cochlear duct. For reasons outlined in the next section, this seems like an improbable scenario, especially for the Höövör specimens. However, even in *Priacodon*, arguably the most plesiomorphic stem therian for which internal petrosal structure is known, it seems likely that the large secondary lamina is a true homolog of the secondary lamina seen in Mesozoic cladotherians and modern therians. For example, the secondary bony lamina in *Priacodon* runs longitudinally onto the crista interfenestralis, that separates the fenestra ovalis and perilymphatic foramen (Figs 7c, 10e and 10f). This is essentially the same positional relationship seen in later cladotherian petrosals [109], suggesting that some association of the abneural side wall of the cochlear canal and the cochlear duct had begun to form even at this early stage in the stem therian lineage. Thus, while its role in the generation and transmission of tension across the basilar membrane may not have been as functional as in modern therians, the appearance of the large secondary lamina may still have served as a mechanism to better match the stiffness between the middle and inner ear [15], or as a means of stabilizing the position of the cochlear duct with respect to the larger cochlear canal.

The status of the middle ear on the other hand can be relatively well characterized in the theriimorph common ancestor, and has been confidently reconstructed as having attained the PDME character state [68] in the earliest forms. Interestingly, many members of this clade show the further calcification of Meckel’s element which forms the structural attachment between the malleus and dentary; a peramorphosis possibly related to the continued reliance on the detection of seismic sound sources through direct conduction even after the mediolateral separation of the postdentary bones from the medial surface of the lower jaw [68]. The fortunate conversion of Meckel’s element into a fossilizable structure therefore provides some possible evidence for the use of non-tympanic low-frequency sound conduction even after the attainment of a true middle ear. However, [110] have advanced arguments for why this element may not be homologous with the embryonic Meckel’s cartilage: if so some of our consideration here would need to be nuanced, but the evidence for non-tympanic conduction is still defensible.

### Trechnotheres

The clade Trechnotheria includes crown therians, the “acute symmetrodonts” (spalacotheroids and amphidontids), various “pre-tribosphenic” taxa (such as *Vincelestes* seen in Fig 13d), and the dryolestoids [36]. While this group is known from a considerable diversity of dental and postcranial remains, the description of Höövör petrosal 1 provided by [4] represents the first tentative information on the external petrosal anatomy in either a basal trechnothere or a closely related stem theriimorphan. The descriptions of the second Höövör petrosal provided here demonstrate several more derived features of H2, making it slightly more likely (but still not certain) that this specimen is referable to Trechnotheria. However, because of the limited amount of information on mammals with this kind of petrosal organization, and possible close relatives of Trechnotheria (especially gobiconodontids) showing broadly similar features, the following characterization of the trechnotherian common ancestor can only be tentative at best.

Probably the most salient feature (if not synapomorphy) seen in the Höövör petrosals and other more confidently referred trechnotherians, is the formation of a true fenestra cochleae (round window) by the bony subdivision of the ancestral perilymphatic foramen (Figs 3a and 7a). This character has uncertain mechanical implications for the isolation and function of the cochlear apparatus. This process is a neomorphic bony strut that bifurcates the ancestral perilymphatic foramen, forming the elongate cochlear aqueduct (containing the membranous perilymphatic duct) dorsomedially and fenestra cochleae ventrolaterally (i.e. Fig 8e; [56,57]). The enclosure of the perilymphatic duct in a bony canal is also seen in some multituberculates and late adult tachyglossids [54], in addition to several groups of squamates and archosaurs [111]. As such the perilymphatic foramen is likely present in the mammalian common ancestor [62,111–113]. Therefore, only about one quarter of the bony “frame” of the round window (the edge of the processus recessus) can be considered an apomorphy; but its significance, if any, is at present unclear. The lack of exposure of a relatively long perilymphatic duct into the middle ear cavity may be considered as significant a transformation as that of the true round window because of its facilitation of a direct connection of the perilymphatic duct with the inner ear.

The most plesiomorphic trechnotherians [4,47,60] also show features related to the increased ventral extension of the pars cochlearis relative to the surrounding pars canalicularis and other cranial elements. These include the more vertical orientation of the crista interfenestralis which, as in the Höövör petrosals described above, terminates caudal to the promontorium without contacting the mastoid area or paroccipital process. The area immediately caudal to the promontorium within the tympanic aspect of the pars canalicularis also forms a post-promontorial tympanic sinus, that is broadly confluent laterally with the lateral trough [54]. In trechnotheres the post-promontorial tympanic sinus increases the volume of the cavum tympani, thereby reducing the effective rigidity of the (primary) tympanic membrane [21]. As also seen in monotremes, the earliest trechnotherians also reduce or lose a robust quadrate ramus of the alisphenoid (epipterygoid), while retaining a thin lateral flange of the petrosal (the ancestral attachment of the quadrate ramus); and similar to several eutriconodontans, one early spalacothere shows a calcified Meckel’s element [114].

### Cladotheres

Defined as the clade containing the common ancestor of therians and the diverse Mesozoic group Dryolestoidea [115,116], a significant characteristic of Cladotheria is the dorsoventral coiling of the cochlear canal, above and beyond the initial lateral (abneural) curvature of the cochlear canal developed in early mammaliaforms [49]. This reconfiguration of the cochlear apparatus is expressed on the ventral surface of the pars cochlearis as the projecting bulbous morphology of the tympanic surface of the promontorium. The cladotherian common ancestor likely also attained a transpromontorial course of the internal carotid artery, as is evidenced by the sulcus left by this vessel on the pars cochlearis.

Ventral bulging of the promontorium is likely not the result of volume restrictions within the pars cochlearis, due to the variably incomplete filling of the bony space available within the pars cochlearis by the cochlear canal [8,63]. However, limitations in the relative dorsoventral linear distance available to the cochlear canal within the pars cochlearis may be responsible for the evolutionary timing of the dorsoventral coiling, as the images described above (Figs 3, 6 and 7) demonstrate that the progressive reduction and loss of the epicochlear and hypocochlear sinuses is a trend taken to near completion in the stem therian circumpromontorial venous plexus before the initiation of dorsovental coiling in cladotheres. It is however clear that basal members of Mammaliaformes and non-therian mammals have substantially more petrosal bone surrounding the cochlear endocast; while therians, have a closer cochlea-pars cochlearis fit.

While all previously described stem cladotherian petrosals from northern continents (i.e., dryolestoids [7,8,49,63] and possibly [105]) and the pre-tribosphenic mammal *Vincelestes* (Fig 13d; [27]) show at most 270° of dorsoventral coiling, cladotherian petrosals from Argentina [6,117,118] demonstrate that complete (360° and beyond) cochlear coiling was attained by later Mesozoic and Cenozoic stem cladotherian lineages in parallel to crown therians.

In Northern Hemisphere dryolestoids, complete cochlear coiling is not known to occur; however, the cochlear endocasts of *Henkelotherium* [7] and *Dryloestes* [8] demonstrate that the suspension of the basilar membrane between a true bony primary lamina and secondary lamina may have developed in these forms, also possibly in parallel to crown therians. In dryolestoids and therians, this increased contact between the endolymphatic cochlear duct and perilymphatic cochlear canal is thought to provide better impedance matching between the middle and inner ears [81], that may have allowed for adaptive increases in maximal detectable frequency. However, theoretical concerns outlined in [84,119] suggest that the gain in selectivity and sensitivity by the cochlear apparatus from these osteological features would be mainly limited to frequencies less than 20 kHz in Mesozoic taxa, where a complete primary lamina is only present basally. The variable extent of cochlear coiling, and presence/absence of bony laminae, and their extent, make the restructuring of the cochlear canal difficult to interpret. However, all cladotherian taxa invariably show the presence of a neomorphic bony armature, termed the tractus foraminosus, for the individual distribution of cochlear nerve fibers and supporting tissues through the foramen acusticum inferius ([63]; although the presence of a tractus foraminosus is ambiguous in the specimen described by these authors). The presence of the foramen acusticum inferius itself, composed of the foramina for the cochlear and sacculoampullar branches of the vestibulocochlear nerve and their derivative and supporting structures, may also be a neomorphic feature of Cladotheria, or cladotheres and their closest trechnotherian relatives (Fig 15f–15i).

### Theria

With the advent of the crown clade Theria, mammals attained the most sophisticated form of airborne sound detection known among terrestrial vertebrates. Members of this clade are typically characterized by the capacity for the detection of ultrasonic frequencies [15]. These capacities are facilitated by the coordinated development of many osteological characters that have been widely commented on in the paleontological literature [24,68,69,120]. Prominent among these are the attainment of a DME, and the completion of at least one full whorl by the dorsoventrally coiled cochlear canal. The level of cochlear coiling in therians is also more derived than that seen in most other cladotheres because of the presence of a modiolar structure (bony armature for the spiraled cochlear nerve) within the concavity defined by the coiled cochlear canal. This morphology causes the peripheral processes of auditory afferent neurons to take on a radial, as opposed to parallel, distribution [120]. However, despite the diverse literature on these and other osteological apomorphies, displayed by all species or particular subsets of crown Theria, precise functional implications of the bony structures of the therian middle and inner ear remain ambiguous [15,23].

Because of their representation by the majority of extant mammals, soft-tissue characteristics within the earliest crown therians can also be reasonably estimated. For instance, unique vascular features that can be inferred to have been present in the petrosal of the therian common ancestor (but likely appearing in their much earlier ancestors) include the almost-completely intracranial course of the superior ramus of the stapedial artery [12], and the loss of the post-trigeminal vein [12,28]. The progressive reduction and loss of the bony canal for the prootic sinus, and reduction of the lateral head vein are also an apparent trend in plesiomorphic members of both major therian clades, Eutheria and Metatheria (Figs 11a–11d and 12a–12d; [12,28,69]). More pertinent for the reconstruction of auditory sensitivity in early therians is their exclusive reliance on the stria vascularis for the production of endolymph (Fig 14e), and their uniquely high-positive endocochlear potential [76,83]. A recent publication [19] hypothesizes that these and other distinguishing features of the therian cochlear apparatus are related to the evolutionary compensation required by the loss of the lagenar macula, yet another unique condition seen in the therian inner ear. The stem therian petrosals described here can be seen as broadly concordant with this hypothesis, and can ostensibly provide boundaries for the reconstruction of auditory performance in the first crown therians and their earlier ancestors.

Concurrent with the evolutionary development of the most sophisticated and high-frequency sensitive ear known among tetrapods, crown therians also sever the last potential vibrational interconnections mediolaterally linking both ears (i.e. the middle ear airspaces on each side of the head are acoustically isolated and independent) by the elongation and stenosis of the lateral parts of the ancestral interaural canal [62,98,121], forming a muscular and facultatively patent eustachian tube. The implications of this increased separation for the reconstruction of sound localization and central processing capacities in early therians are discussed below; however, fossilizable evidence of this trend in interaural isolation is shown by the widespread and homoplastic development of ossified bullae within many crown therian lineages [61], which are lacking in non-therian mammals (for evidence of functionally analogous “pseudobullae” present in specialized multituberculates see [122]).

### Significance of stem therian petrosals

#### Phylogenetic placement of the Höövör petrosals

As outlined in the above descriptions, the amount of morphological similarity between H1 and H2 is much greater than the similarity between either specimen and *Priacodon*, or any extant mammalian taxon. This similarity is in fact closer than originally appreciated by [5], because of the mistaken character scoring of the condition of the perilymphatic duct in H2 used in their phylogenetic analysis. As can be confirmed from the above descriptions, the membranous perilymphatic duct in both H1 and H2 had a direct confluence with the inner ear by way of its bony enclosure within the aqueductus cochleae (the bony perilymphatic canal; Fig 10a–10d). As such, the close similarity between H1 and H2 in terms of size, morphology, and provenance supports the hypothesized sister-relationship [5] between the two taxa represented by these specimens (Fig 2). However, despite the close morphological resemblance between H1 and H2, we treat these specimens as distinct at the species level, if not higher. This is because of the many instances of contrasting morphology between H1 and H2 outlined in the above descriptions (such as the presence of a hypocochlear sinus and relatively large fenestra semilunaris in H1, compared to H2), which we consider to be outside the plausible range of intraspecific variation. Additionally, we suggest that the above descriptions of the external anatomy of H2, and the internal features of both Höövör petrosals, also do not provide a decisive conclusion to the problem of the phylogenetic attribution of these specimens with respect to the mammalian taxa known from dental remains recovered from Höövör.

However, for the following reasons, the hypothesis that the Höövör petrosals represent both of the major stem therian lineages known from dental elements at this locality is still defensible as one of the two most probable hypotheses for the phylogenetic assignment of these specimens (the other, and equally probable, hypothesis is that they are both gobiconodontid taxa). First, irrespective of the relative relationship between the H1 and H2, the robust development of caudal and lateral structures on H1 (Fig 7a) strongly suggest its affinity as a gobiconodontid. For example an elongate and robust paroccipital process is also seen in *Repenomamus*, a close relative of the gobiconodontids [123]. While correlates of relative robustness have not previously been scored and used in phylogenetic analyses, these features are likely associated with the mediolaterally widened mandibular condyle seen in gobiconodontids and their nearest relatives (such as *Repenomamus*). Additionally, relative to H2, H1 shows a shorter rostrocaudal extent of the floor of the cavum supracochleare (measured as the length of the bony lamina flooring the space for the facial ganglion), and the tympanic aperture of the prootic canal positioned more posterolaterally to the secondary facial foramen (“tapc” in Figs 3a and 7a), both features also seen in the “triconodonts” described by [46]. While the clear presence of a cochlear aqueduct, processus recessus, and true fenestra cochleae are characteristics which have not been reported in prior descriptions of gobiconodontid cranial remains, they have been observed in gobiconodntids (GWR Pers Obs) and are possibly present in other members of the likely non-monophyletic group Eutriconodonta (e.g. the evidence for a cochlear aqueduct is inconclusive in the amphilestid taxon *Juchilestes* [124]). If the evidence supporting the gobiconodontid affinities of H1, and the possible trechnothere affinities of H2 is reliable, then based on the known roster of dental remains recovered from Höövör, the most likely taxonomic attribution for H1 would be to the fossil species *Gobiconodon borissiaki*.

While the similarities and suggested sister relationship between H1 and H2 have been remarked on here and by [5] (making it inconclusive as to which if either of these taxa are referable to *G*.*borissaiki*), many of the advanced features of H1 suggest its more proximate relationship with trechnotherian mammals. These features are all detailed in the above descriptions, but include the general reduction of venous proliferation within the pars cochlearis and loss of the hypocochlear sinus (Fig 3b). Additionally, the greater development of the vein of the cochlear aqueduct and its confluent sulcus on the abneural wall of the scala tympani (“hs” in Fig 13f), and closer approximation of the foramen for the sacculoampullar branch of the vestibular nerve to the foramen for the cochlear nerve near to the level of the crista transversa (Fig 15f and 15g) are plausible apomorphies in H2. Additionally, aside from not displaying the above mentioned “gobiconodontid” characteristics seen in H1, H2 also does not display the apparently apomorphic lack of the fossa incudis on the petrosal (as seen in H1 [4]). Even though the fossa incudis is not a preserved structure in H2, the separation of the fragmentary base of the mastoid region from its sutural surface for the squamosal demonstrates that these two regions would not have been in overlapping contact (as in H1 they are, causing the fossa incudis to be lost or relocated to the squamosal bone in this taxon) (Fig 7a). The advanced features seen in H2, combined with the gobiconodontid features seen in H1, support, among the taxa described from Höövör, a closer phylogenetic affiliation of H2 with “symmetrodontan” mammals within the clade Trechnotheria. Under this interpretation the most likely dentally sampled taxon to which the H2 petrosal could be referred to is the tinodontid genus *Gobiotheriodon* [125,126]; however the rarity of these specimens makes specific assignment unreliable, and we prefer to suspend the family-level taxonomic attribution of both Höövör petrosals until more material and character information is available. The plesiomorphic trechnotherian status of H2 is also supported by the less laterally deflected margin of the tympanic surface (Fig 3a), suggesting that the glenoid fossa would have been located far posterolaterally, possibly distal to a pedicle-like concavity (“post-glenoid depression”) on the posterior root of the zygomatic arch. This condition is seen in *Zhangheotherium* and *Maotherium* [47,64]. Unlike *Zhangheotherium*, however, the mediolateral extent of the medial margin of the pars cochlearis is considerably wider in both H1 and H2.

While the new information on the Höövör petrosals provided here does not definitively resolve the phylogenetic location of these specimens, their status as stem therians, somewhat more closely related to crown therians than *Priacodon*, is secure (as reported in [5]; see Fig 2). The following functional implications of the morphology seen in these specimens relies only on the status of these petrosals as stem therians.

#### Osteological correlates of the stria vascularis

The current narrative of mammalian auditory evolution has been assembled predominantly from insights gained from morphological and developmental sources (e.g. [127,128]). Less accessible inner ear structures have been difficult to observe. Because most unique features of the therian auditory percept rely on apomorphic histological and physiological characteristics, such as the large and tortuous stria vascularis and the highly positive endocochlear potential located inside the inner ear, the relative silence on these aspects represent a deficit in our understanding [129].

One under-emphasized question that is apparent in the inner ear of all extant therians is the conflicting set of demands placed on the auditory hair cells, which are required to alternately transmit and resist transduction currents at unparalleled rates while simultaneously being forced to function at a far remove from the vasculature providing for their nutrition, waste removal, and oxygenation [30,75]. While the use of potassium as opposed to sodium is an adaptation for metabolic efficiency seen in the hair cells of all vertebrates [80,83], the demands for high performance in the therian auditory endorgan seems to place this group’s auditory hair cells in a unique metabolic crisis. In response to this, the relatively large stria vascularis and uniquely formulated endolymphatic composition developed as a partial solution [2,19,130,131]. Because monotremes retain an ancestral endolymph-producing capillary plexus in Reissner’s membrane (see Fig 14b and 14f; [17,18]) in addition to the stria vascularis, and a greater number of radially oriented vessels crossing the cochlear duct [79], they seem to be less liable to metabolic distress than therians. However, the lamentable lack of physiological research on the cochlear apparatus of extant monotremes makes it impossible to judge whether the less specialized anatomy of the cochlear apparatus and supporting vasculature is attributable to weaker cochlear performance compared to therians, or because of some currently unrecognized requirement for increased cochlear vascularization developed in monotremes [75,84].

Because of the ease of misinterpreting bony anatomy in extinct animals at both anatomical and functional levels, caution must be used when attributing significance to any morphological novelty. However, when seen against the wider trend of reduction of the circumpromontorial venous plexus in mammaliaforms generally, the localized venous hypertrophy at the sole intersection of the circumpromontorial venous plexus and cochlear endocast strongly suggests an adaptive significance for the vein of the cochlear aqueduct (VCAQ) at its earliest known instantiation in the Höövör petrosals described here. Because the stria vascularis is the most prominent organ within the abneural cochlear duct in both mammalian lineages bracketing the phylogenetic location of the Höövör specimens, the most reasonable functional attribution for this neomorphic vein is that it alleviated congestion within the hypertrophied stria vascularis in stem therians as it became the predominant or sole endolymph producing organ (Figs 10a–10d and 14d). It is reasonable that this morphological innovation is itself a response to the increasing demands on the stria vascularis for the production of the high endocochlear potential and the potassium recycling biochemistry seen in extant therians [75,131]. In extant therians, these functions of the stria vascularis make it one of the most metabolically demanding organs in the body, with specific requirements for arteriovenous and capillary ramifications within its parenchyma. The physiologically expensive nature of the stria vascularis is also reflected in extant mammals by its intricate and developmentally complex structure, which requires contribution from both cranial mesoderm (ectomesenchyme) and other specialized neural crest cell populations (e.g. the melanocyte-like strial intermediate cells), and in adults contains one of the only vascularized epithelial tissues known in mammalian anatomy [78]. While other vertebrate groups do show contributions from several embryonic tissue sources in the membranous lining of the membranous labyrinth, nowhere outside of theria do these cell populations reach the level of organization seen in the therian stria vascularis [71,78]. It is therefore fortunate that this unique organ requires an enlarged and conspicuous vasculature detectable in the cochlear endocast of ancestral therians (Fig 13c–13f).

The neomorphic appearance of the VCAQ in the Höövör petrosals may also reflect a more general reorganization of the vascular supply to the cochlear duct, allowing for the withdrawal of those blood vessels that radially span the unadhered membrane exposed toward the scala tympani and scala vestibuli sides of the cochlear duct (Fig 14d and 14e). Because the formation of the sulcus for the VCAQ seen in the Höövör petrosals (“hs” in Fig 10b and 10c) represent the first extensive bony integration of the vasculature of the cochlear duct into the cochlear canal seen in Mammaliaformes, it is probable that the tissues of the membranous abneural wall of the cochlear duct relied to some extent on vasculature reaching it through its area of adhesion to the spiral ligament and bony cochlear canal (Fig 14a–14f). This is the situation seen in extant therians (e.g. [132]) where the VCAQ and other vessels do not cross the free basilar and vestibular surfaces of the cochlear duct (Fig 14e). This removal of blood vessels radially spanning the basilar and especially vestibular membranes is an apomorphic feature of the cochlear duct seen only in extant therians, and allows for the acoustic isolation of low-frequency interference generated by hemodynamic pulsations, away from the highly sensitive cochlear endorgan and supporting structures. As mentioned above, in extant monotremes and sauropsids the membranous tissues of the cochlear duct do not adhere to the bony side walls of the cochlear canal, leaving this structure mechanically unsupported and requiring that all blood vessels supplying the auditory epithelia travel in radial and longitudinal directions across the full length and circumference of the cochlear duct [18,25,79].

While the veins servicing the stria vascularis are the only soft-tissue component of the mammalian cochlear duct to leave a recognized osteological correlate, the high performance of the stria vascularis in modern therians is predicated on the presence and precise functioning of many unique molecular, cellular, and histological structures. The acquisition and localized hypertrophy of the vein of the cochlear aqueduct is therefore likely associated with the pre-existent or incipient presence of unfossilizable characteristics of the therian-style cochlear apparatus. Chief among these features is the reinforced compartmentalization of the endolymphatic and perilymphatic spaces, necessitated by the requirement to limit paracellular diffusion of potassium and calcium salts between these fluids (e.g. [133]). In extant therians, the stable attachment of the cochlear duct to the spiral ligament within the cochlear canal allows for the segregation and recycling of potassium and other ions through interconnected epithelial and connective tissue syncytia [130,133]. For therians, this fluid homeostasis is reliant on the efficient transfer of material through the slender and precariously located processes of root cells within the abneural cochlear duct [133], which could be liable to mechanical damage if left unsupported within the free membrane of the cochlear duct in monotremes and other non-therians. The single foramen and linear sulcus for the vein of the cochlear aqueduct within the cochlear canal endocasts of the Höövör petrosals, as opposed to a highly perforated and reticulating venous network present in non-therians, may also support the potassium recycling function of the stria vascularis and cochlear syncytia by allowing salts resorbed into the VCAQ an opportunity to diffuse back into the intrastrial space before being conducted into systemic venous circulation outside the otic capsule (e.g. see [130]).

Even though nothing has been reported on the composition or electrical potential of endoymph in extant monotremes, it has been observed that the effects of voltage changes in OHC membranes are not as dramatic as those seen in therian mammals, and that monotreme prestin has a peak non-linear capacitance voltage optimum which is far from the cellular resting potential of OHCs [134,135]. This makes the electromotile function of prestin inefficient in monototremes. The calcium concentration of monotreme endolymph is likely also much higher than the ~ 20 micromolar concentration seen in the cochlear duct of most therians, because of the continued existence of the lagenar otoconial mass in this group and the need to prevent uncontrolled dissolution of this mass [19].

#### Osteological correlates of macromechanical tuning

The problem of transducing airborne sound into an electrical signal and its further decomposition into spectral components, are a sophisticated functionality endowed to the cranium of many terrestrial vertebrates. As summarized in [16] the convergent innovations for airborne sound perception present in extant anurans, archosaurs, squamates, and mammals all operate with comparable levels of sensitivity and selectivity for frequencies under approximately 8 kHz. At the level of the auditory endorgan, the strategies for frequency selectivity (tuning) seen in these groups are effected through a mixture of molecular, bony, and histological adaptations. The field of comparative hearing [136] therefore can invaluably inform hypotheses of auditory capacities in extinct amniotes through the ancestral reconstruction of symplesiomorphic character states [2,84]. Inferences about which of several non-mutually exclusive tuning mechanisms are present in early mammals are necessarily based on the distribution of the several forms of tuning in extant mammals, or predicted for the last common ancestor of amniotes [101,137]. Because of this dependence on extant representatives, the almost complete lack of physiological studies on the living monotremes in particular imposes a major obstacle to our understanding of tuning mechanisms in the synapsid lineage [77,138].

What is currently known about the distribution of tuning mechanisms across tetrapods suggests a major dichotomy in strategy between ancestral electrical tuning (see Supplementary Material 1; [139]), and several forms of mechanical tuning [101]. The varieties of mechanical tuning can be conceptually decomposed into intrinsic (action at the level of the hair cell) versus extrinsic (action at the organ level or larger), and active (requiring cellular energy) versus passive (based on inert geometrical and material properties) mechanisms (creating a total of four discrete categories; [137]). All of these tuning mechanisms are characterized by some degree of tonotopy (the correlation of best frequency response with anatomical linear distance) within the auditory endorgan, and therefore a corresponding Space Constant (SC) expressed in units of millimeters per octave (an octave is a doubling of frequency). The extant amniotes that rely solely on the plesiomorphic mechanism of electrical tuning (*Sphenodon* and the chelonians), show some of the smallest SC values (0.3 mm per octave or smaller) because of the extremely short length of their auditory papilla. As such, these forms, and most likely all early amniotes, show only short and undifferentiated sacculocochlear recesses within their labyrinthine endocasts. Conversely, in all non-mammalian amniotes showing a differentiated middle ear, a form of mechanical tuning is emphasized that relies on tonotopic variation in the mass, stiffness and number of stereovilli of the hair cells within the auditory papilla (homolog of the mammalian organ of Corti). This mechanism is instantiated in active (molecularly driven motion of stereovilli) and passive forms [101]. This category of tuning is dependent on organelle-level features present within the auditory hair cells themselves and so is termed micromechanical tuning; and it is associated with an intermediate range of SC values (less than 1 mm per octave up to several mm per octave; [16]). Because of the elongate but absolutely short cochlear canals seen in the earliest mammaliaforms (such as *Morganucodon*; [13,87,140]), it has been hypothesized that micromechanical tuning provided the initial impetus for the development of the bony cochlear canal within the synapsid lineage as well [19]. However, in all known extant mammals (monotremes and therians) no evidence for significant electrical or micromechanical tuning has been physiologically recorded ([129]; although see [102] for possible evidence of active micromechanical tuning). Where characterized best in advanced therians, the sole form of tuning is based on an apomorphic extrinsic mechanism termed macromechanical tuning. Evaluating the performance of this form of tuning among synapsids depends on the cochlear length and the SC. Fossils are often amenable to cochlear measurement, but the SC can only be approximated in general terms. However, it is not until mammaliaforms achieve cochlear canals long enough to accommodate at least several octaves (somewhere near the emergence of Crown Mammalia; Fig 16) that macromechanical tuning is likely to enable auditory capabilities similar to living mammals. That being said, therians with extremely low body mass may show extremely short cochlear canals, such as *Sorex* with a cochlear canal length of 2.54 mm [24]. We are unaware of studies reporting SC values for *Sorex* or other such diminutive therians, but likely these forms achieve SC values well below 1 mm per octave because of their observed use of broadband echolocation clicks in the range of 20–95 kHz [141]. If this is correct, at least some modern therians are able to achieve ultrasonic audition in conjunction with macromechanical tuning of very short cochleae.

Even though little is known about tuning modalities in extant monotremes, there are strong reasons to suspect that somewhere along the backbone of synapsid evolution, between earliest mammaliaforms and crown therians, a shift toward extrinsic macromechanical tuning and away from more plesiomorphic forms of tuning should be recognizable. The presumed reliance on macromechanical tuning in mammals is likely also reflected by the universal presence of a true organ of Corti, with its functional differentiation of Inner and Outer Hair Cells (described above) and characteristic arrangement of membranes within the cochlear duct [109].

While the presence of macromechanical tuning appears to be consistently present within Mammalia, there is an obvious spectrum in terms of its performance (e.g. sensitivity, selectivity, and highest detectable frequency) and its morphological/molecular accommodation across mammalian species; with the monotremes defining the lower end and eutherians the higher end of the spectrum [2]. This spectrum is also recognizable in both the active and passive mechanisms of macromechanical tuning [101]. For instance, wide scale comparative studies on the structure of the prestin protein [134] estimate that monotreme prestins are much less capable of useful electromotility at physiological voltages. Additionally, monotremes show a lower proportion of Outer Hair Cells (expressing prestin on their basolateral surface) to Inner Hair Cells [109] when compared to therians. These observations support the generally comparable level of sensory traffic, but weaker instantiation of the macromechanical active process in the monotremes.

The macromechanical passive process is effected by the gradient in compliance of the basilar membrane, and its monotonic increase in width and decrease in depth (thickness) as it runs apically beneath the organ of Corti [101,142]. Therefore, the passive process is present even in an inert and lifeless basilar membrane; and its capacity to tonotopically propagate traveling waves is modulated by the geometry of the cochlear duct [143], and its lateral attachments to the cochlear canal, or lack thereof [144,145]. The concerted action of this mechanical arrangement filters the range of frequencies presented to each individual auditory hair cell, allowing each cell to specialize for the transduction of a filtered frequency bandwidth [101]. Morphological features suggesting a weaker commitment to the macromechanical passive process in monotremes include their large and relatively untapered basilar membrane (which is wider than most of the largest and lowest frequency basilar membranes in found in extant therians; [109]), and lack of any adherence of the basilar membrane (or any other part of the endolymphatic cochlear duct) to rigid supports within the bony cochlear canal [18].

The stem therian labyrinthine endocasts described here contain apparently synapomorphic osteological features which allow them to be placed along the spectrum of passive macromechanical adaptation present in extant monotremes and therians. In particular, *Priacodon* and both Höövör petrosals differ from early mammaliaforms and monotremes, and resemble the more derived cladotherians, in the presence of a well-developed secondary bony lamina (Fig 14c and 14d). When present in modern therians, the secondary bony lamina provides a rigid attachment for the abneural cochlear duct and contributes to the concentration and transmission of tensile forces across the basilar membrane (Fig 14e; [23]). The first appearance of a well-formed secondary bony lamina in these stem therian endocasts (Figs 10, 14c and 14d) is therefore strong evidence for the abneural adhesion of the membranous cochlear duct with the bony cochlear canal, and may also signify the presence of the spiral ligament and its specialized populations of fibroblasts (e.g. tension fibroblasts; [146]). These soft-tissue specializations are known to be associated with the secondary lamina in extant therians and are critical for normal therian hearing function. However, as mentioned above, lack of an opposing primary bony lamina in *Priacodon* and the Höövör petrosals, or even an ossified tractus foraminosus, makes it unlikely that tensile forces similar to those in therians could be transmitted across the basilar membrane through its attachment along the secondary bony lamina in these forms. The stem therians described here therefore show an intermediate level of passive macromechanical adaptation by showing some rigid mechanical support for the cochlear duct, but lack the diametrically opposing primary bony lamina required for the catenary suspension of the basilar membrane, which is associated with the tonotopic region of frequencies greater than 10 kHz in modern therians [15]. Macromechanical active and passive processes were likely also evolutionarily associated with each other [134]. As such, it is reasonable to hypothesize that taxa showing a bony secondary (or primary) lamina represent useful phylogenetic calibrations for the initiation of features supporting rapid electromotility in the prestin molecule [134], a capacity which is known to be present, but much more weakly instantiated in modern monotremes [135].

Finally, the formation of a true round window (fenestra cochleae) and perilymphatic canal (aqueductus cochleae), such as that seen in both Höövör specimens (Figs 3a and 7a), does not have a clear functional interpretation but has been hypothesized as improving the vibrational insulation of the inner ear, allowing for the synchronized, opposite-phase pulsations of the primary and secondary tympanic membranes [109]. Conversely, the first appearance of these features and the development of a process recessus may have been initiated as a structural byproduct of increasing braincase width and associated lateral displacement of the perilymphatic foramen relative to the jugular foramen.

This comparative and fossil evidence for macromechanical tuning in the earliest crown mammals, especially along the therian stem, strongly suggests that a cochlear space constant within the range of values associated with macromechanical tuning in extant mammals would be applicable to these fossil members of crown Mammalia as well [14,15]. The lower limit of mammalian SC values in extremely small mammals is currently unknown, and the anecdotally suggested average SC value of 2.5 mm per octave (based on an unidentified sample of eutherians including rodents, bats and other small-medium sized forms [14,15,99]) is likely very approximate and not readily applicable to non-therians. This makes inferences regarding the frequency limitations of early mammals uncertain. Because of the highly nested position of shrews and other minute eutherians in mammalian phylogeny, and their highly derived acoustic behavioral characteristics [147], we consider that extremely small macromechanical SC values in these forms are equally problematic for representing the primitive condition for theria in general and for the earliest mammals in particular. When measured in a generalized therian (*Monodelphis domestica*; [148]), SC values varied along the auditory epithelium ranging from ~1.5 mm per octave along the basal 60% of the cochlea to 1.8 mm per octave maximally and 0.8 mm per octave in a limited region near the apex of the cochlear duct. The ~40 mm skull length seen in *M*. *domestica* makes it comparable to the estimated size of the taxon represented by H1 [4]. The larger marsupial *Didelphis*, and the placental *Tupaia* [24,149,150] show typical SC values above 1 as well. Living monotremes also appear to also show a SCs above 1; the ~3 octave effective frequency range reported for *Tachyglossus* by [151] corresponds to an estimated SC value of 2.3 mm per octave using the cochlear measurements provided in [18]; while *Ornithorhynchus* has an estimated SC above 1 [18,152]. Additionally, it is likely that early mammals were receptive to very narrow frequency bandwidths; e.g. monotremes show 3–5 octave range and 4 octave range is reported for the lesser hedgehog tenrec *Echinops* [153], and generalized marsupials have a range at, or below, ~5 octaves [154].

Given the ~4 mm length of the cochlear duct in the stem therian endocasts described here, and their position in the mammalian “phylogenetic bracket”, a range of four or five octaves is a skeptically large estimate for the frequency bandwidth available to these forms. The relationship between the bandwidth of detectable frequencies and maximum detectable frequency is, however, also dependent on the lowest detectable frequency. Given a relatively high low-frequency limit for small amniotes of 500 Hz (many modern mammals, lizards and birds have even lower limits, ~ 50–200 Hz) and using a skeptical SC of 0.8 as seen in a small portion of the cochlea in *Monodelphis* [148], the upper frequency limit for these stem therians would be at ~ 8–16 kHz. If given a lower frequency limit of 100 Hz typical of plesiomorphic amniotes the corresponding upper limit would be much lower at ~ 3.2–6.4 kHz [25]. The generous 500 Hz estimate of a low-frequency limit is likely unrealistic, given that it requires the abandonment of the range of many important environmental sounds at low-frequencies in exchange for a relatively very low upper frequency limit (similar to the upper frequency limit seen in modern *Ornithorhynchus* [17,152]). The probable retention of a lagenar macula in *Priacodon* would also diminish the frequency band available to this taxon as well, by taking up ~ 1 mm of length of the cochlear canal (Fig 13h; [81]). The most realistic upper frequency limits for *Priacodon* and the Höövör specimens is therefore likely less than16 kHz, and therefore not within the ultrasonic range. The absence of ultrasonic capability in our fossil taxa is therefore supported even adopting highly skeptical values for the low frequency limit (500Hz, likely in the vicinity of 100Hz), SC value (0.8, likely above 1) and the cochlear length (utilizing the whole length of the endocast, and dismissing the possible presence or remnant of an apical lagena).

However, it is important to emphasize that even under the extreme assumption that macromechanical tuning was entirely absent in early mammals, and therefore space constants within the macromechanical range would not be applicable to the stem therians described here, no form of micromechanical or electrical tuning would feasibly have allowed the upper frequency limit to extend above approximately 16 kHz either [2,16]. Therefore, despite the existence of several autapomorphic high-frequency non-mammalian tetrapods (e.g. [155,156]), and several therian species with very short cochlear canals [24], no combination of the phylogenetically widespread forms of auditory tuning would have allowed the stem therians described here to detect ultrasonic frequencies (~20 kHz or higher; [16]).

Many of the earliest fossil therians are very small [37, 157,158] and are either known to have, or predicted to have, cochlear canals within the same range of sizes as the stem therians described here, and smaller [24]. These extremely small fossil therians, many of which have petrosals just a few of millimeters long are also known to have the osteological correlates of macromechanical tuning seen in living therians (coiled cochleae, Rossenthal’s canal, primary and secondary lamina, etc.) and in several instances cochlear lengths below 5mm [24,120]. Comparisons with modern minute therians with macromechanical tuning suggest that: either 1) these fossil therians were sensitive to very narrow frequency bandwidths because of their very short cochlear canals; and/or 2) the allometric relationship between body mass and SC values seen in extant minute therians, such as in some soricids, would have been present in these earliest fossil therians as well. Both of these hypotheses ultimately rest on the optimization of auditory features on a phylogenetic tree including modern taxa with known auditory bandwidth/SC and the relevant small fossil therians. At present we lack both, the pertinent information on the smallest extant therians, and the proper phylogenetic context integrating fossil and recent taxa. We, therefore choose to refrain from speculating about the auditory capacities of fossil therians within this smallest body-size range.

#### Osteological correlates of the lagenar endorgan (or lack thereof)

The hair cells comprising the lagenar macula are perhaps the most variable and least understood of the many epithelial cell types found around the heterogeneous endolymphatic lining of the pars inferior of the membranous labyrinth [138]. This is especially so in the case of extant mammals, where in the monotreme lineage this sensory epithelium is located between the scala medial and scala vestibuli within a specialized lagenar sac [18]; while extant therians are unanimous in their lack of any adult morphological expression of the lagena altogether. Still, several inducible genetic atavisms seen in rodent models suggest that the distal (low-frequency) extent of the therian organ of Corti persists as the syngenetic homolog of the lagenar macula [95,138].

Nonetheless, as a discrete organ, supporting an otolithic mass and recruiting the innervation of a dedicated lagenar nerve and ganglion, the absence of the lagena is one of the unique and important features of the therian inner ear. It has also been suggested that the loss of the lagenar macula acted as a proximal cause, or immediate correlate, of the development of several other synapomorphic features of therian cochlear physiology, such as the extremely low calcium concentration and high electrical potential of therian endolymph [19,75]. In sauropsids, and presumably also the earliest synapsid taxa, a high (several hundred micromolar up to 1 millimolar; [19]) calcium concentration of the cochlear fluids is required to support electrical tuning and protect the lagenar otolithic mass. A minimal ambient calcium concentration is also known to be required to prevent dissolution of vestibular gravistatic structures [159] which (where examined in the otoconia of modern rodents; [160]) show compositional turnover on a monthly timescale. The released obligation to generate high-calcium endolymph within the cochlear duct likely allowed stem therians the adaptational leeway to reformulate several aspects of their cochlear biochemistry, resulting in the low (~20 micromolar) calcium concentration in cochlear endolymph, and the correspondingly sharp calcium gradient along the membranous labyrinth generally [161,162]. There is also some evidence that the capillary plexus in Reissner’s membrane is specifically associated with the support of the lagenar otolithic mass because of the retention of localized Calcium ATPases at this membrane in extant rodent models [131]. The increasingly exclusive reliance on the mammalian stria vascularis for endolymph production occurring during the evolution of crown therian mammals may therefore be directly correlated with the lost capacity to support a functional lagenar endorgan [19].

The timing and functional significance of lagenar loss is complicated by the lack of pertinent comparative and paleontological evidence. In particular, the lack of experimental recordings of the normal functioning of the lagenar endorgan in sauropsids and monotremes, and conflicting hypotheses regarding the relative importance of its gravistatic versus auditory modalities [25,82,138], make the physiological implications of lagenar loss in stem therians difficult to interpret. The available fossil material is also ambiguous because of the loose osteological association seen between the lagena and the surrounding skeleton of the otic capsule. Bony structures such as an apical inflation of the cochlear canal, and sulci or canals for the lagenar nerve, are variably present among mammaliaforms; however, based on the wider distribution of the lagena among amniotes, a functional lagenar macula almost definitely existed in synapsid taxa lacking such osteological correlates, both preceding the early mammaliaforms, and succeeding them within the mammalian crown group. Therefore, absence of evidence for a functional lagenar endorgan is not evidence of its absence, and it is likely that early stem therians such as *Priacodon* that lack obvious osteological correlates of a lagenar macula retained it nonetheless. This is supported by the presence of lagenar correlates in multituberculates, which branch near *Priacodon* in stem therian phylogeny (Fig 2; [68,163]); and the lack of bony features indicating a switch to the modern therian-style of cochlear physiology predicated on the lack of the lagenar macula [19,46,82]. The reference to the loss of the lagena as “the Cretaceous Cochlear Revolution” [19] may therefore be misleading in that it is currently unclear as to whether the morphological expression of the lagena was lost in an evolutionarily punctuated event or a gradual interval of decreasing usefulness. Based on the stratigraphic distribution of fossil therians, and most phylogenetic hypotheses of their relationships (i.e [36,164] inter alios), the loss of the lagena also certainly occurred within the Jurassic if not earlier, and likely several times.

The internal bony anatomy visible in the high-resolution scans of the Höövör petrosals provide the earliest indications of advanced features seen today only in therian mammals. As outlined above, several of the osteological features seen in these specimens suggest that the cochlear duct achieved at least some adhesion to the abneural margin of the cochlear canal (Fig 15c–15e) and show increased (if not exclusive) reliance on the functioning of the stria vascularis as an endolymph producing organ [83]. These characteristics are unique to therian mammals among extant vertebrates, and (combined with the presence of a straight and distally tapering cochlear canal in the Höövör cochlear endocasts; Figs 10a–10d, 13e and 13f) strongly suggest that these stem therians have greatly reduced or lost the lagenar macula altogether. These forms could therefore have attained other soft tissue characteristics seen in modern therians such as a terminal helicotrema [18].

The consistent presence of dorsoventral coiling to accommodate the lengthened cochlear canal seen in later cladotherian mammals may have then been enabled by the lagenar macula reorienting to a position where its sensory input was mostly or exclusively responsive to vertical linear acceleration [17], and therefore completely redundant to stimulus from the saccule and utricle [19]. Another hypothesis based on the physical modeling presented in [143] suggests that cochlear coiling represents an adaptation for the conduction and concentration of low-frequency vibrations of the basilar membrane along the abneural margins of the apical cochlear canal, thereby increasing sensitivity to these frequencies relative to an uncoiled cochlear canal. Under this hypothesis, the extremely straight cochlear morphology as seen in H1 and H2 (compared to the condition in several stem mammaliaforms [13,31,90]) may actually be seen as a modification de-emphasizing the sensitive detection of lower-frequency sounds. Whatever the original selective pressure for dorsoventral coiling, the complete loss of the remnant hypocochlear sinus in therians may also be an effect of dorsoventral expansion of the cochlea within the pars cochlearis, or a combination of factors.

The high likelihood that the Höövör petrosals described here were beneficiaries of the “cochlear revolution” provides a useful perspective on the selective regime responsible for the loss of the lagena and the development of the unique therian cochlear physiology. Particularly, because of criteria outlined in the discussion of macromechanical tuning, there was almost no capacity for high-frequency (above ~ 20 kHz) hearing in these early stem therians. Therefore, whatever factors led to lagenar loss must not be related to the development of ultrasonic hearing capacities. This is complementary to hypotheses that stem therians relied to a substantial extent on substrate based (i.e. non-tympanic) sound conduction [68,165]; more importantly, this evidence is contradictory to the hypothesis that these unique features of the therian inner ear are related to the unique capacity of extant therians to detect and localize ultrasonic sound sources [13,77]. As with the innovation of the three ossicle middle ear found in mammaliamorphs, the unique inner ear mechanisms developed in ancestral stem therians (advanced theriimorphans or early trechnotheres) developed in service of acoustic performance within an ancestral frequency range, and later proved capable of being extended into ever higher frequencies in their descendant therian taxa. The late Cretaceous meridiolestidan *Coloniatherium* [6], with a fully coiled cochlea and a centrally located modiolus, optimizes in most phylogenies as an independent acquisition of these features. A highly derived inner ear morphology (with complete coiling, and without a lagenar inflation) is also present in more the plesiomorphic taxon *Cronopio* [166], and terminal taxa such as *Peligrotherium* [118] and *Necrolestes* [9,117]. The detailed similarities between meridiolestidan and therian inner ears, with their potential auditory convergences, have yet to be fully explored.

#### Auditory localization in stem therians

Aside from the difficulties related to the sustenance of greater mass-specific caloric requirements, within topographically more heterogenous habitats, small mammals are faced with novel challenges for the segregation and localization of sound sources. This “small mammal problem” has been a central focus in the traditional explanation for the advent of ultrasonic hearing in therian mammals, because of the requisite use of high frequencies for sound localization at small body sizes (e.g. [109]). Indeed, the abilities of even very “primitive” small mammalian insectivores to utilize broadband and high-frequency cues for auditory localization, even to the point of echolocation in many cases, is well recorded (e.g. [147]). Therefore, it is probable that ultrasonic capacities evolved in the earliest crown therians in response to selection for greater localization capabilities; however, the evidence reported here of the likely very low upper detectable frequency limits in stem therians, and the manifest capacity of small-bodied sauropsids to locate sound sources with frequencies below 5 kHz, suggest that traditional narratives of the evolution of ultrasonic hearing require qualification [3,33,77,167,168].

The one-dimension waveform presented to each ear carries very limited, and difficult to extract, information on the spatial location of its source. These difficulties in localization are both physical and computational in nature. For instance, the physical coupling of sound frequencies in air ranging from 20 Hz– 20 kHz to corresponding wavelengths ranging from 17 m– 17 mm (respectively), require sensitivity to frequencies with corresponding wavelengths larger than the head size (interaural distance) of many low-frequency limited terrestrial vertebrates. The many sauropsid groups to have developed a tympanic middle ear have convergently solved this physical challenge by the coupling of both right and left tympanic membranes through the medial air mass comprising their interaural canal (i.e the cavum tympani and other contiguous cavities). This has the effect of increasing interaural delay times and allows the detection of interaural phase differences at the level of the auditory transducers themselves, alleviating the neurological requirement to develop a complex internal representation of binaural differences within the central nervous system [169,170]. This “pressure-gradient receiver” form of auditory localization is the only mechanism for the perception of low-frequency limited sound sources known in terrestrial vertebrates; and it possibly also evolved in early eucynodonts and later synapsid taxa with angular tympanic membranes [171,172].

Conversely, modern therian mammals have developed a predominantly computational strategy for the localization of sound sources, based on the isolated functioning of both ears as simultaneous and independent “pressure receivers”. The adaptations providing this capacity are categorized into monaural and binaural mechanisms, each making specific minimal demands for broadband and high-frequency (near-ultrasonic) hearing. They are also commonly specialized for vertical and azimuthal localization, respectively. All of these mechanisms also make substantial demands on the central nervous system, such as the required detection of binaural coincidence on submillisecond timescales, and novel processing in the lower auditory brainstem [168,173]. Because of the conflicting demands that the pressure-gradient receiver versus pressure receiver forms of auditory localization place on the specific structure of the external, middle, and inner ear, the synapsid lineage must have reduced and ultimately lost its pressure gradient receiver capacities before the advent of the modern form of therian sound localization (if pressure gradient receivers existed in early synapsid taxa at all). The sequential development of the MME, PDME, DME, and development of discrete bullae in the therian crown group, may therefore represent the progressive reduction and eventual loss of the ancestral mechanisms for sound localization. Likewise, the presence of a broadly open interaural canal in some extant therians [174] could plausibly be interpreted as atavisms to a pre-mammalian condition. The platypus *Ornithorhynchus* [62] also shows a patent interaural communication which in fact narrows substantially before merging with the proximal segment of the pharynx. Depending on how the primitive condition for the last common ancestor of amniotes is reconstructed the condition of *Ornithorhynchus* could be primitive, or that of the echidnas with a recognizable eustachian tube would be symplesiomorphic for Mammalia. At present we consider this question unresolved and the primitive mammalian condition equivocal [172].

**Binaural sound localization.** The stem therian taxa described here most likely attained a PDME state of the middle ear [68], and therefore would have attenuated or lost the tentatively ancestral pressure-gradient capacity for sound localization if indeed it had previously existed. However, regardless of whether or not the pressure-gradient receiver mechanism operated in earlier members of the synapsid lineage, the functional limitations imposed by the short length and macromechanical adaptations seen in the stem therian cochlear canals described above would also have precluded the useful functioning of the modern sound localization strategies seen in extant therians. The two strategies seen in extant therians most useful for azimuthal localization are based on the comparison of stereo binaural input, and are termed the Interaural Time Difference (ITD) and Interaural Level Difference (ILD) mechanisms [3]. These two mechanisms are based on the capacity to contrast the time of arrival of distinctive spectral features (ITD) or the instantaneous amplitude of the stimulus (ILD), respectively. However, the ability to precisely contrast arrival times of spectral features is contingent on a sufficiently large binaural time delay, which itself is a function of the Functional Head Size (FHS a metric of interaural distance measured in microseconds; [33]) of an animal. Therefore, ITD based localization is emphasized to the exclusion of ILD in modern therians with large FHS values (e.g. domesticated ungulates). Conversely, modern small mammals (with smaller than 200 microseconds FHS) emphasize ILD, and small mammals using only one binaural cue use ILD. Many of the known modern small therians that use both ITD and ILD are low-frequency specialist rodents [33,127,175]. As such, even with a possibly convoluted interaural canal extending the binaural time delays somewhat [120], the estimated FHS of very approximately 50 microseconds or less in the crown mammalian common ancestor [3, 176, 177] would place the earliest mammals within the predominantly ILD size range. This inference is also supported by the purportedly more plesiomorphic construction of the ILD circuitry within the lateral surperior olive in the therian Superior Olivary Complex [173].

However, while it is very unlikely that the first stem therians were able to use ITD as a localizing mechanism, the low estimated upper frequency limit of the stem therian cochlear endocasts presented here also suggests that the ILD mechanism would also have been inoperative or inefficient in these animals as well. While the exact frequency requirements for ILD functioning are somewhat variable across extant therian species, because the attenuation of sound amplitude is produced by cranial “shadowing” of the stimulus as it propagates across the tissues of the head, ILD requires frequencies with corresponding wavelengths shorter than the interaural distance of that particular taxon [178]. In the case of the small stem therians presented here, and with a very generous estimate of interaural distance of ~1 inch, the corresponding minimal ILD frequencies of ~13 kHz would likely be just marginally within or above the upper frequency limits of these small taxa. However, while it is likely that the lowest frequencies required for ILD were perceptible by the stem therians described here, the proper functioning of the ILD mechanism (and the other localization mechanisms) in extant small therians relies on the availability of a wide band of frequencies beginning with the lowest usable frequency. In the case of stem therians, the ILD mechanism would therefore have been poorly functional relative to its performance in modern therians if it had been present at all [168]. This is also complementary with what has been suggested as the most likely evolutionary trajectory for the assembly of the modern neural circuitry supporting ILD, where the hypothesized incipient stages of binaural processing was likely only sufficient for the segregation of discrete simultaneous sound sources, and possibly their relative localization [173].

**Monaural Sound Localization.** Therefore, while the capacity for sound localization based on the physical interconnection of both ears, or the simultaneous comparison of the electronically encoded input from both ears, would be inoperative or poorly functional at best in stem therians, the final method of auditory localization known in extant tetrapods does actually have some empirical support from the fossil record of Mesozoic mammals. This final form of auditory localization is based on the spectral alteration of monaural input (termed Head-Related Transfer Functions; [173]) by the presence of a specialized external pinna. In extant therians this pinna-based form of auditory localization is most important in front-back discrimination of sound sources, and in specialized species allows the vertical localization of sound sources, such as along a mid-sagittal plane. Where tested in llamas, frequencies ~ 3 kHz and higher allow for consistent resolution of the front versus back location of a sound source [33]. In cats tested with frequencies greater than ~ 10 kHz, the vertical location of sounds is resolvable, as is the precise location of a sound source within each ear’s “cone of confusion” [179]. As summarized above, the anatomical evidence of an involuted pinna in *Tachyglossus* [106] and the excellent soft tissue preservation in the gobiconodontid *Spinolestes*, suggest that an elaboration of the external ear may have been present in crown mammals (and was likely present in early theriimorphs; [107]). The mammalian pinna may have initially appeared as an inefficient but sufficient method to monaurally localize sound sources, possibly only for front-back discrimination (there is also a significant amplification effect provided by the pinna as well; [21]). The more sophisticated capacities for monaural vertical localization, predicated on minimal frequencies higher than approximately 10 kHz, likely only developed in crown therians and their close relatives as near-ultrasonic frequencies became detectable within the therian lineage. While the observation, provided in [2], that the dimensions of the soft tissue impression of the external pinna in *Spinolestes* would most efficiently provide localizing information (such as spectral cues) at frequencies above 20 kHz, for the reasons outlined above is appears unlikely that these types of advanced localization mechanisms were present in early theriimorphs such as *Spinolestes* and the Höövör specimens.

This hypothesis regarding the acoustic capacities in Jurassic and Early Cretaceous stem therians, synthesized from both paleontological and physiological evidence, presents an unimpressive picture of the ancestors of modern therian mammals as poorly equipped, possibly nocturnal, insectivores compared to modern standards. While many extant small mammals show sophisticated sound localization capacities, even with frequencies below 20 kHz, it seems that early stem therians would not have attained a sufficient bandwidth of frequencies, and/or a high enough upper frequency limit to have been able to usefully localize short duration sounds faster than visual localization alone [33]. This may reflect the less competitive nature of the small insectivore niche within Mesozoic terrestrial ecosystems, but could equally support the presence of non-tympanic forms of conduction such as the hypothesized direct conduction of sound through Meckel’s element as suggested by [68]. The combination of poor auditory localization abilities with adaptations suggesting increased sensitivity and selectivity at low frequencies in stem therians may therefore be a compromise between the low-frequency requirements for seismic sound conduction and the increasingly specialized capabilities for airborne sound localization.

It is currently ambiguous what behavioral and autecological implications the transitional morphology of these stem therian petrosals have for the reconstruction of Mesozoic mammals. However, it is important to reiterate that the inference of poor sound localization capabilities within the early stem therians is not based on any single ancestral reconstruction of the cochlear tuning mechanism or auditory physiology within the crown mammalian ancestor or first stem therians. All phylogenetically common cochlear tuning mechanisms typical of modern amniotes, working individually or in combination, would be equally incapable of extending the upper frequency limit in the fossil taxa described here to frequencies above ~ 16 kHz. If, as seems most likely, macromechanical tuning was present within the early theriimorphans and trechnotheres, the relatively large SC values associated with this type of tuning commonly seen in extant therians would prevent the short cochleae in these fossil forms from extending into frequency ranges much higher than 16 kHz.

Even if a more ancient form of intrinsic tuning (electrical or micromechanical) was present in the earliest stem therian mammals, the low upper frequency limits associated with these forms of tuning in modern tetrapods would also cut-off the maximal detectable frequencies in stem therians to under ~ 10 kHz (as described above exceptions to this frequency limit are seen only in very specialized and phylogenetically restricted taxa among sauropsids and amphibians, e.g. [155,156]).

## Conclusions

The descriptions and discussion provided here highlight the phylogenetically heterogeneous nature of stem therian petrosal evolution throughout the Mesozoic. The minimal age for the the successive nodes formed by *Priacodon*, the Höövör petrosals, and therians is currently dated as Early and Middle Jurassic, respectively (determined by the eutriconodontan *Argentoconodon* [180] and the therian *Juramaia* [181]); despite major differences in morphology, the internodal age difference is only on the order of 10–15 MY. The approximately 50 million year duration separating the stratigraphic provenance of the Upper Jurassic Priacodon and Lower Cretaceous Höövör specimens focused on here is simply the result of taxon sampling, illustrating the first known acquisitions of several derived internal labyrinthine features along the backbone of therian evolution. Even before the advent of the dorsoventral cochlear coiling characteristic of modern crown therians and their cladotherian relatives, both *Priacodon* and the Höövör petrosals show morphologies suggestive of greater acoustic performance (in terms of selectivity and sensitivity) unique to this lineage within Mammalia.

Osteologically, these specimens demonstrate that several of the internal labyrinthine features appearing in the earliest mammaliaforms (e.g. lateral curvature of the cochlear endocast and lagenar inflation) were lost before most of the advanced cladotherian morphological features related to cochlear coiling and the bony support of the cochlear nerve appeared (Fig 10). Interestingly, this evolutionary loss of lateral curvature of the cochlear canal before the advent of its dorsoventral coiling is not matched by the developmental trajectory of the membranous cochlear duct; as seen in rodent models [128,182,183], lateral curvature and subsequent dorsoventral coiling form two discrete stages in the development of the membranous labyrinth [184]. Additionally, the ontogenetic initiation of hearing in humans and a variety of model organisms seems to parallel the hypothesized evolutionary transformation of the cochlea, with the hair cells responding to low-frequencies changing from basal to apical regions during normal development [185]. Dorsoventral coiling may then ultimately be evolutionarily associated with a rudimentary adaptation of the mammalian cochlea for the preservation of frequency sensitivity in the ancestral (low-frequency) range [143].

For reasons outlined above, it also seems likely that the perceptual capacity for sound source localization was undeveloped or rudimentary in early mammaliaforms. The hypothetical pressure gradient receiver form of auditory localization was either reduced or absent before the capacity to use advanced ILD and ITD forms of localization developed in the immediate ancestors of crown therians. The localization mechanism most likely to be present in the forms described here would have been based on monaural pinna-based signals, for which the preservation of an external pinna in one exceptionally preserved theriimorph specimen can be considered evidence [107]. However, even using these pinna-based cues, this form of localization in the stem therians described here would mostly be competent for front-back localization of sound sources.

This is not to suggest that early stem therians displayed poor hearing capacities generally, and the presence of a salient secondary bony lamina within the cochlear endocasts described here (“sl” in Fig 10a, 10c and 10e) suggests some amount of adhesion between the cochlear duct and spiral ligament with the abneural cochlear canal (Fig 15c and 15d). This is an advanced level of structural organization beyond the state seen in even modern monotremes, and is likely associated with a greater commitment to macromechanical tuning than that seen in extant monotremes. The attachment of the basilar membrane to the newly evolved secondary lamina before the advent of the primary bony lamina also suggests that the basilar membrane in these forms was less tense and stabilized than is typical of modern therians. However, it is also likely that the low-frequency limitations of the stem therians described here would not have precluded these forms from relying on an insect-based diet, which has been predicted as the mainstay of most generalized Mesozoic lineages including those represented by the fossils described here [186–190]. One study [191] estimates that at least one Middle Jurassic katydid species produced frequencies (~6.4 kHz) which would very plausibly be detectable by these stem therians.

This report details the bony features pertinent for the phylogenetic and soft tissue reconstruction of *Priacodon*, Höövör petrosal 1 and especially the newly described Höövör petrosal 2 (Figs 1–10). However, perhaps the most significant aspect of cochlear morphology presented by these specimens is what they entail regarding the rate of high-frequency adaptation near the therian crown group. If the uniquely derived and phylogenetically unstable clades Gondwanatheria and Multituberculata are excluded, the theriimorph specimens used here provide a phylogenetic bracket around the advanced clade Cladotheria. To date the majority of previously described Mesozoic mammalian petrosal specimens belong to Cladotheria, and several convergent derivations of the fully coiled cochlear canal, tractus foraminosus (convergent with monotremes; Fig 15b and 15e), and primary bony lamina are likely within this group. The petrosals described here corroborate the slow rate of upper frequency limit increase in the synapsid lineage up to the advent of Cladotheria; and that cladotheres may therefore be thought of as an evolutionary radiation into a high-frequency world [192]. Within the Cretaceous, both crown therians and South American dryolestoids both achieve a structurally “modern” fully-coiled form of the cochlear canal [6,85]. This may have been a response to selection for sound-source localization, particularly the capacity to locate brief environmental cues faster than visual inspection alone [3,33]. However, our hypothesis that this capacity was lacking, or poorly developed, in the immediate stem therian ancestors of the cladotheres suggests that the central or peripheral processing of sound in these early forms was either incapable of modern therian performance parameters, or an appropriate selective pressure for high frequency hearing was absent in earlier Mesozoic environments. The lack of clear selective advantage may in turn be attributable to evolutionary compromises between high-frequency requirements for sound localization and possible behavioral requirements for low-frequency perception (such as non-tympanic sound conduction); or the uncompetitive nature of the small insectivore niche in the Mesozoic. Whatever the original impetus for the development of ultrasonic frequency sensitivity, the segregation of terrestrial vertebrate faunas into a high-frequency therian component and low-frequency sauropsid component has persisted from the Jurassic to the present day.

## Supporting information

S1 File Evolution of the synapsid ear. Phylogenetic and anatomical background: the premammalian ear region.
(DOCX)

## Abbreviations

?ptv: possible location of post-trigeminal vein
ac: aqueductus cochleae (for perilymphatic duct)
acf: aperture of cochlear fossula (external to fenestra cochleae)
adm: arteria diploëtica magna
al: anterior lamina
amp-a: anterior semicircular canal ampulla
amp-h: horizontal semicircular canal ampulla
amp-p: posterior semicircular canal ampulla
av: aqueductus vestibuli (for endolymphatic duct)
bpsal: border of periosteal surface of anterior lamina
bs: basisphenoid sutural surface
cas: cavum supracochleare
cc-p: primary common crus
cc-s: secondary common crus
cdh: centripetal diverticulum of horizontal semicircular canal
ce: cavum epiptericum
ci: crista interfenestralis
coc: cochlear canal
cVIII: cochlear branch of vestibulocochlear nerve
dag: dorsal ascending groove
eapc: endocranial aperture of prootic canal
ect: ectotympanic bone
eps-p: posterior epicochlear sinus (“trans-cochlear sinus p” in [31])
fac: facial canal (aqueductus Fallopii in [32])
fc: fenestra cochleae (for secondary tympanic membrane)
fcn: foramen for cochlear nerve
FHS: functional head size (in microseconds)
fs: fenestra semilunaris
fv: fenestra vestibuli (fenestra ovalis)
gpn: greater petrosal nerve
H1: Höövör petrosal specimen 1 (PSS-MAE-104)
H2: Höövör petrosal specimen 2 (PSS-MAE-119)
hF: hiatus Fallopii (for greater petrosal nerve)
hfn: hyomandibular branch of facial nerve
hps: hypocochlear sinus
hs: half-pipe shaped sulcus
iam: internal acoustic meatus
IHC: inner hair cell
ijv: internal jugular vein
ILD: interaural level difference
ips: inferior petrosal sinus
ir: inferior ramus of stapedial artery
ITD: interaural time difference
jn: jugular notch
lf: lateral flange
lhv: lateral head vein
li: lagenar inflation
mm: manubrium of malleus
ntr-a: notch for temporal ramus a
ntr-b: notch for temporal ramus b
OHC: outer hair cell
pcs: sinus around prootic canal
pfc: prefacial commissure (suprafacial commisure)
pf: perilymphatic foramen
pop: paroccipital process
pos: paroccipital sinus
pov: prootic vein
pptc: petrosal contribution to post-temporal canal
re: recessus ellipticus (for utricle)
rpm: rostral (anterior) process of malleus (= goniale/ prearticular)
rso: ramus supraorbitalis
rs: recessus sphericus (for saccule)
saf: subarcuate fossa
sas: subarcuate venous sinus
sa: stapedial artery
SC: cochlear space constant (in octaves/ mm)
sff: secondary facial foramen
sf: stapedius fossa
sl: secondary (abneural) bony lamina of cochlear canal
sm: sulcus medial to promontorium
sr: superior ramus of stapedial artery
ssc-a: anterior semicircular canal
tapc: tympanic aperture of prootic canal
tf: tractus foraminosus
tph: tympanohyal
tr-a: anterior temporal ramus
tr-b: posterior temporal ramus
uaf: foramen for utriculoampullar branch of vestibular nerve
vag: ventral ascending groove
vcaq: bony canal accommodating vein of the cochlear aqueduct (Canal of Cotugno)
VII: facial nerve
vVIII: vestibular branch of vestibulocochlear nerve

## References

[1] Rowe TB. Definition, diagnosis, and the origin of Mammalia. J Vertebr Paleontol. 1988;8: 241–264.

[2] Köppl C, Manley GA. A functional perspective on the evolution of the cochlea. Cold Spring Harb Perspect Med. 2018; 10.1101/cshperspect.a033241 30181353 PMC6546037

[3] Grothe B, Pecka M, McAlpine D. Mechanisms of sound localization in mammals. Physiol Rev. 2010;90: 983–1012. 10.1152/physrev.00026.2009 20664077

[4] Wible JR, Rougier GW, Novacek MJ, McKenna MC, Dashzeveg D. A mammalian petrosal from the Early Cretaceous of Mongolia: Implications for the evolution of the ear region and mamaliamorph interrelationships. Am Mus Novit. 1995;3149: 1–19.

[5] Rougier GW, Wible JR, Hopson JA. Basicranial anatomy of *Priacodon fruitaensis* (Triconodontidae, Mammalia) from the Late Jurassic of Colorado, and a reappraisal of mammaliaform interrelationships. Am Mus Novit. 1996;3183: 1–38.

[6] Rougier GW, Forasiepe AM, Hill RV, Novacek M. New mammalian remains from the Late Cretaceous La Colonia Formation, Patagonia, Argentina. Acta Paleontol Pol. 2009;54: 195–212

[7] Ruf I, Luo ZX, Wible JR, Martin T. Petrosal anatomy and inner ear structures of the Late Jurassic *Henkelotherium* (Mammalia, Cladotheria, Dryolestoidea): insight into the early evolution of the ear region in cladotherian mammals. J Anat. 2009;214: 679–693. 10.1111/j.1469-7580.2009.01059.x 19438763 PMC2707092

[8] Luo ZX, Ruf I, Martin T. The petrosal and inner ear of the Late Jurassic cladotherian mammal *Dryolestes leiriensis* and implications for ear evolution in therian mammals. Zool J Linn Soc. 2012;166: 433–463.

[9] Wible JR, Rougier GW. Craniomandibular anatomy of the subterranean meridiolestidan *Necrolestes patagonensis* Ameghino, 1891 (Mammalia, Cladotheria) from the early Miocene of Patagonia. Ann Carnegie Mus. 2017;84: 183–252.

[10] Archer M. The basicranial region of marsupicarnivores (Marsupialia), interrelationships of carnivorous marsupials, and affinities of the insectivorous marsupial peramelids. Zool J Linn Soc. 1976; 59: 217–322.

[11] Asher RJ, Novacek MJ, Geisler JH. Relationships of endemic African mammals and their fossil relatives based on morphological and molecular evidence. J Mammal Evol. 2003;10: 131–194.

[12] MacPhee RD. Auditory reigions of primates and eutherian insectivores. Basel: S. Karger; 1981.

[13] Rosowski JJ, Graybeal A. What did *Morganucodon* hear?. Zool J Linn Soc. 1991;101: 131–168.

[14] Manley GA. Cochlear mechanisms from a phylogenetic viewpoint. Proc Natl Acad Sci. 2000;97: 11736–11734. 10.1073/pnas.97.22.11736 11050203 PMC34343

[15] Manley GA. The foundations of high-frequency hearing in early mammals. J Mammal Evol. 2016;25: 155–163.

[16] Manley GA. Comparative auditory neuroscience: Understanding the evolution and function of ears. J Assoc Res Otolaryngol. 2017;18: 1–24. 10.1007/s10162-016-0579-3 27539715 PMC5243258

[17] Ashwell KW. Auditory and vestibular systems In: Ashwell K, editor. Neurobiology of monotremes. Collingwood: CSIRO Publishing; 2013 pp. 219–233.

[18] Schultz JA, Zeller U, Luo ZX. Inner ear labyrinth anatomy of monotremes and implications for mammalian inner ear evolution. J Morphol. 2017;278: 236–263. 10.1002/jmor.20632 27889918

[19] Manley GA. The mammalian Cretaceous cochlear revolution. Hearing Res. 2017;352: 23–29. 10.1016/j.heares.2016.12.007 28007525

[20] DeBeer GR. The development of the vertebrate skull. Oxford: Clarendon Press; 1937.

[21] Moore WJ. The mammalian skull. Cambridge: Cambridge University Press; 1981.

[22] Wible JR. Petrosal anatomy of the nine-banded armadillo, *Dasypus novemcinctus* Linnaeus, 1758 (Mammalia, Xenarthra, Dasypodidae). Ann Carnegie Mus. 2010;79: 1–28.

[23] Meng J, Fox RC. Osseus inner ear structures and hearing in early marsupials and placentals. Zool J Linn Soc. 1995;115: 47–71.

[24] Ekdale EG. Comparative anatomy of the bony labyrinth (inner ear) of placental mammals. PLOS One. 2013;8: 1–100. 10.1371/journal.pone.0066624 PMC3689836 23805251

[25] Wever EG. The reptile ear: Its structure and function. Princeton: Princeton University Press; 1978.

[26] Wible JR. Transformations in the extracranial course of the internal carotid artery in mammalian phylogeny. J Vertebr Paleontol. 1986;6: 313–325.

[27] Rougier GW, Wible JR, Hopson JA. Reconstruction of the cranial vessels in the Early Cretaceous mammal *Vincelestes neuquenianus*: Implications for the evolution of the mammalian cranial vascular system. J Vertebr Paleontol. 1992;12: 188–216.

[28] Wible JR, Hopson JA. Homologies of the prootic canal in mammals and non-mammalian cynodonts. J Vertebr Paleontol. 1995;15: 331–356.

[29] Janfaza P, Nadol JB Jr, Galla R, Fabian RL, Montgomery WW. Surgical anatomy of the head and neck. Cambridge: Harvard University Press; 2011.

[30] Nomura Y. Morphological aspects of inner ear disease. Tokyo: Springer Japan; 2014.

[31] Panciroli E, Schultz JA, Luo Z. Morphology of the petrosal and stapes of Borealestes (Mammaliaformes, Docodonta) from the Middle Jurassic of Skye, Scotland. Papers in Paleontol. 2018;

[32] Kermack KA, Musset F, Rigney HW. The skull of *Morganucodon*. Zool J Linn Soc. 1981;71: 1–158.

[33] Heffner HE, Heffner RS. The evolution of mammalian sound localization. Acoustics Today. 2016;12: 20–27.

[34] Belyaeva EI, Trofimov BA, Reshetov VY. General stages in the evolution of late Mesozoic and early Tertiary mammalian faunas in central Asia. Trudy Sovmestnoi Sovetsko Mongol’skoi Paleontologicheskoi Ekspeditsii. 1974;1: 19–45.

[35] Benton MJ. Conventions in Russian and Mongolian palaeontological literature In: Benton MJ, Shishkin MA, Unwin DM, Kurochkin EN, editors. The age of dinosaurs in Russia and Mongolia. Cambridge: Cambridge University Press: 2000 pp. xvi–xxxix.

[36] Kielan-Jaworowska Z, Cifelli RL, Luo ZX. Mammals from the age of dinosaurs: Origins, evolution, and structure. New York: University of Colombia Press; 2004.

[37] Wible JR, Rougier GW, Novacek MJ, McKenna MC. Earliest eutherian ear region: a petrosal referred to *Prokennalestes* from the Early Cretaceous of Mongolia. Am Mus Novit. 2001;3322: 1–44.

[38] Dashzeveg D. *Kielantherium gobiensis*, a primitive therian from the Early Cretaceous of Mongolia. Nat. 1975;227: 402–403.

[39] Dashzeveg D. *Arguimus khosbajari* gen. n., sp. n., (Peramuridae, Eupantotheria) from the Lower Cretaceous of Mongolia. Acta Palaeontol Pol. 1979;24: 199–204.

[40] Lopatin AV, Averianov AO. *Kielantherium*, a basal tribosphenic mammal from the Early Cretaceous of Mongolia, with new data on the aegialodontian dentition. Acta Palaeontol Pol. 2007;52: 441–446.

[41] Lopatin AV, Averianov AO. The stem placental mammal *Prokennalestes* from the Early Cretaceous of Mongolia. Paleontol J. 2017;51: 1293–1374.

[42] Lopatin AV, Averianov AO. A new stem placental mammal from the Early Cretaceous on Mongolia. Doklady Biol Sci. 2018;478: 8–11. 10.1134/S0012496618010027 29536398

[43] Rasmussen TE, Callison G. A new species of triconodont mammal from the Upper Jurassic of Colorado. J Paleontol. 1981;55: 628–634.

[44] Callison G. Fruita: A place for wee fossils In: Averett W editor. Paleontology and geology of the dinosaur triangle. Grand Junction: Grand Junction Geological Society: 1987 pp. 91–96.

[45] Engelmann GF, Callison G. Mammalian faunas of the Morrison Formation. Mod Geol. 1998;23: 343–379.

[46] Kermack KA. The cranial structure of the triconodonts. Phil Trans R Soc Lond B. 1963;246: 83–103.

[47] Hu Y, Wang Y, Luo Z, Li C. A new symmetrodont mammal from China and its implications for mammalian evolution. Nature. 1997;390: 137–142. 10.1038/36505 9367151

[48] Allin EF, Hopson JA. Evolution of the auditory system in Synapsida (“mammal-like reptiles” and primitive mammals” as seen in the fossil record In: Webster DB, Fay RR, Popper AN, editors. The evolutionary biology of hearing. Heidelberg: Springer-Verlag: 1992 pp. 587–614.

[49] Luo Z, Ruf I, Schultz JA, Martin T. Fossil evidence on evolution of inner ear cochlea in Jurassic mammals. Proc R Soc Lond B. 2011;278: 28–34. 10.1098/rspb.2010.1148 PMC2992725 20667879

[50] Han G, Mao F, Bi S, Wang Y, Meng J. A Jurassic gliding euharamiyidan mammal with an ear of five auditory bones. Nature. 2017;551: 451–546. 10.1038/nature24483 29132143

[51] Flower WH. An introduction to the osteology of Mammalia, 3^rd^ ed London: Macmillan; 1885.

[52] MacIntyre GT. The trisulcate petrosal pattern of mammals In: Dobshansky T, Hecht M, Steere WC, editors. Evolutionary Biology, Vol. 6 New York: Appleton-Century-Crofts: 1972 pp. 275–303.

[53] DeBeer GR. The development of the skull of the shrew. Phil Trans. R Soc Lond B. 1929;217: 411–480.

[54] Rougier GW, Wible JR. Major changes in the ear region and basicranium of early mammals In: Carrano MT, Gaudin TJ, Blob RW, Wible JR, editors. Amniote paleobiology: Perspectives on the evolution of mammals, birds, and reptiles. Chicago: Chicago University Press; 2006 pp. 269–311.

[55] Orliac MJ, O’Leary MA. The inner ear of *Protungulatum* (pan-Euungulatum, Mammalia). J Mam Evol. 2016;23: 337–352.

[56] Bast TH. Development of the aquaeductus cochleae and its contained periotic duct and cochlear vein in human embryos. Ann Otol Rhinol Laryngol. 1946;55: 278–297. 10.1177/000348944605500204 20993452

[57] Bast TH, Anson BJ. The development of the cochlear fenestra, fossula and secondary tympanic membrane. Ann Otol Rhinol Laryngol. 1954;62: 1083–1116. 10.1177/000348945306200413 13114833

[58] Diogo R, Abdala V, Lonergan N, Wood BA. From fish to modern humans–comparative anatomy, homologies and evolution of the head and neck musculature. J Anat. 2008;213: 391–424. 10.1111/j.1469-7580.2008.00953.x 18657257 PMC2644766

[59] Kielan-Jaworowska Z, Presley R, Poplin C. The cranial vasculature system in taeniolabidoid multituberculate mammals. Phil Trans R Soc Lond. 1986;313: 525–602.

[60] Rougier GW, Ji Q, Novacek MJ. A new symmetrodont mammal with fur impressions from the Mesozoic of China. 2003;77: 7–14.

[61] Novacek MJ. Patterns of diversity in the mammalian skull In: Hanken J, Hall BK, editors. The skull, volume 2: Patterns of structural and systematic diversity. Chicago: University of Chicago Press; 1993 pp. 438–545.

[62] Zeller U. Ontogenetic evidence for the cranial homologies in monotremes and therians, with special reference to *Ornithorhynchus* In: Szalay FS, Novacek MJ, McKenna MC, editors. Mammal phylogeny: Mesozoic differentiation, multituberculates, monotremes, early therians, and marsupials. New York: Springer; 1993 pp. 95–107.

[63] Hughes EM, Wible JR, Spaulding M, Luo ZX. Mammalian petrosal from the Upper Jurassic Morrison Formation of Fruita, Colorado. Ann Carnegie Mus. 2015;83: 1–17.

[64] Turkewitsch BG. Comparative anatomical investigation of the osseous labyrinth (vestibule) in mammals. Am J Anat. 1935;57: 503–543.

[65] MacIntyre GT. Foramen pesudovale and quasi-mammals. Evol. 1966;21: 834–841. 10.1111/j.1558-5646.1967.tb03437.x 28563083

[66] McDowell SB. The Greater Antillean insectivores. Bull Am Mus Nat Hist. 1958;115: 113–214.

[67] Wible JR. The eutherian stapedial artery: character analysis and implications for superordinal relationships. Zool J Linn Soc. 1987;91: 107–135.

[68] Luo ZX, Schultz JA, Ekdale EG. Evolution of the middle and inner ears of mammaliaformes: the approach to mammals In: Clack JA, Fay RR, Popper AN, editors. Evolution of the vertebrate ear: Evidence from the fossil record. Cham: Springer; 2016 pp. 139–174.

[69] Ekdale EG. The ear of mammals: From monotremes to humans In: Clack JA, Fay RR, Popper AN, editors. Evolution of the vertebrate ear: Evidence from the fossil record. Cham: Springer; 2016 pp. 175–206.

[70] Ruf I, Luo ZX, Martin T. Reinvestigation of the basicranium of *Haldanodon expectatus* (Mammaliaformes, Docodonta). J Vertebr Paleontol. 2013;33: 382–400.

[71] Axelsson A. Comparative anatomy of cochlear blood vessels. Am J Otolaryngol. 1988;9: 278–290. 3067591 10.1016/s0196-0709(88)80036-x

[72] Lempert J, Meltzer PE, Wever EG, Lawrence M, Rambo JHT. Structure and function of the cochlear aqueduct. AMA Archives of otolaryngol. 1952;55: 134–145. 10.1001/archotol.1952.00710010143003 14894025

[73] Perlman HB. Experimental occlusion of the inferior cochlear vein. Ann Otol Rhinol Laryngol. 1952;61: 33–44. 10.1177/000348945206100103 14915396

[74] Forasiepi AM, Rougier GW. Additional data on early Paleocene metatherians (Mammalia) from Punta Peligro (Salamanca Formation, Argentina): comments based on petrosal morphology. J Zool Syst Evol Res. 2009;47: 391–398.

[75] Nin F, Yoshida T, Sawamura S, Ogata G, Ota T, Higuchi T, et al The unique electrical properties in an extracellular fluid of the mammalian cochlea; their functional roles, homeostatic processes, and pathological significance. Pflugers Arch Eur J Physiol. 2016;468: 1637–1649. 27568193 10.1007/s00424-016-1871-0 PMC5026722

[76] Wilms V, Köppl C, Söffgen C, Hartmann AM, Nothwang HG. Molecular bases of K+ secretory cells in the inner ear: Shared and distinct features between birds and mammals. Sci Rep. 2016;6: 1–13. 27680950 10.1038/srep34203 PMC5041087

[77] Manley GA. The cochlea: What it is, where it came from, and what is special about it In: Manley GA, Grummer AW, Popper AN, Fay RR, editors. Understanding the cochlea. Cham: Springer; 2017 pp. 17–32.

[78] Kikuchi K, Hilding DA. The development of the stria vascularis in the mouse. Acta otolaryngologica. 1966;62: 277–291. 10.3109/00016486609119573 5956511

[79] Pritchard U. The cochlea of the *Ornithorhynchus platypus* compared with that of ordinary mammals and of birds. Phil Trans R Soc Lond. 1881;172: 267–282.

[80] Schmidt RS, Fernández C. Labyrinthine DC potentials in representative vertebrates. J Cell Phys. 1962;59: 311–322. 10.1002/jcp.1030590311 13908794

[81] Manley GA. A review of some current concepts of the functional evolution of the ear in terrestrial vertebrates. Evol. 1972;26: 608–621. 10.1111/j.1558-5646.1972.tb01968.x 28563349

[82] Manley GA, Haeseler C, Brix J. Innervation patterns and spontaneous activity of afferent fibres to the lagenar macula and apical basilar papilla if the chicken’s cochlea. Hear Res. 1991;56: 211–226. 1685157 10.1016/0378-5955(91)90172-6

[83] Köppl C, Wilms V, Russell IJ, Nothwang HG. Evolution of endolymph secretion and endolymph potential generation in the vertebrate inner ear. Brain Behav Evol. 2018; 10.1159/000494050 30415265

[84] Manley GA. Evolutionary paths to mammalian cochleae. J Assoc Res Otolaryngol. 2012;13: 733–743. 10.1007/s10162-012-0349-9 22983571 PMC3505590

[85] Wible JR. Petrosals of Late Cretaceous marsupials from North America, and a cladistic analysis of the petrosal in therian mammals. 1990;10: 183–205.

[86] Evans HE, De Lahunta A. Miller’s anatomy of the dog. St Louis: Elsevier health sciences; 2013.

[87] Graybeal A, Rosowski JJ, Ketten DR, Crompton AW. Inner-ear structure in *Morganucodon*, and early Jurassic mammal. Zool J Linn Soc. 1989; 96: 107–117.

[88] Luo Z, Crompton AW, Lucas SG. Evolutionary origins of the mammalian promontorium and cochlea. J Vertebr Paleontol. 1995;15: 113–121.

[89] Hossler FE, Olson KR, Musil G, McKamey MI. Ultrastructure and blood supply of the tegmentum vasculosum in the cochlea of the duckling. Hearing Res. 2002;164: 155–165. 10.1016/s0378-5955(01)00427-0 11950535

[90] Shahid R, Gil GG, Hoffmann S. Inner ear morphology of basal-most mammaliaform *Morganucodon*. The FASEB Journal, No 1 Supplement. 2018; 32: 780 Available from https://www.fasebj.org/doi/abs/10.1096/fasebj.2018.32.1_supplement.780.9

[91] Spoor F, Zonnefeld F. Morphometry of the primate bony labyrinth: A new method based on high-resolution computed tomography. J Anat. 1995;186: 271–286. 7649826 PMC1167185

[92] Rodrigues PG, Ruf I, Schultz CL. Digital reconstruction of the otic region and inner ear of the non-mammalian cynodont *Brasilitherium riograndensis* (Late Triassic, Brazil) and its relevance to the evolution of the mammalian ear. J Mammal Evol. 2013;20: 291–307.

[93] Olson EC. Origin of mammals based upon cranial morphology of the therapsid suborders. Spec Pap Geol Soc Am. 1944;55: 1–136.

[94] MacLean PD. Neurobehavioral significance of the mammal-like reptiles (theriapsids) In: MacLean PD, Roth JJ, Roth EC, editors. The ecology and biology of mammal-like reptiles. Washington: Smithsonian Institution Press: 1986 pp. 1–21.

[95] Butler AB, Hodos W. Comparative vertebrate neuroanatomy: Evolution and adaptation. 2nd ed Hoboken: John Wiley and Sons Inc; 2005.

[96] Rowe TB. The emergence of mammals In: Kaas JH, editor. Evolution of nervous systems, second edition, volume 2 Oxford: Elsevier; 2017 pp. 1–52.

[97] Rich TH, Hopson JA, Musser AM, Flannery TF, Vickers-Rich P. Independent origins of middle ear bones in monotremes and therians. Science. 2005;307: 910–914. 10.1126/science.1105717 15705848

[98] Urban DJ, Anthwal N, Luo Z, Maier JA, Sadier A, Tucker AS. A new developmental mechanism for the separation of the mammalian middle ear ossicles from the jaw. Proc R Soc B. 2017;284: 1–8. 10.1098/rspb.2016.2416 PMC5310609 28179517

[99] Vater M, Kössl M. Comparative aspects of cochlear functional organization in mammals. Hearing Res. 2011;273: 89–99. 10.1016/j.heares.2010.05.018 20630478

[100] Manley GA, Köppl C. Phylogenetic development of the cochlea and its innervation. Curr Opin Neurobiol. 1998;8: 468–474. 9751658 10.1016/s0959-4388(98)80033-0

[101] Fettiplace R, Fuchs PA. Mechanisms of hair cell tuning. Ann Rev Physiol. 1999;61: 809–834. 10099711 10.1146/annurev.physiol.61.1.809

[102] Chan DK, Hudspeth AJ. Ca 2+ current-driven nonlinear amplification by the mammalian cochlea in vitro. Nature Neurosci. 2005;8: 149–155. 10.1038/nn1385 15643426 PMC2151387

[103] Peng AW, Ricci AJ. Somatic motility and hair bundle mechanics, are both necessary for cochlear amplification. Hear Res. 2011;273: 109–122. 10.1016/j.heares.2010.03.094 20430075 PMC2943979

[104] Ladhams A, Pickles JO. Morphology of the monotreme orgen of Corti and macula lagena. J Comp Neurol. 1996;366: 335–347. 10.1002/(SICI)1096-9861(19960304)366:2<335::AID-CNE11>3.0.CO;2-O 8698891

[105] Prothero DR. The oldest mammalian petrosals from North America. J Paleontol. 1983;57: 1040–1046.

[106] Augee ML, Gooden B, Musser A. Echidna: Extraordinary egg-laying mammal. CSIRO Publishing; 2006.

[107] Martin T, Marugán-Lobón J, Vullo R, Martín-Abad H, Luo ZX, Buscalioni AD. A Cretaceous eutriconodont and integument evolution in early mammals. Nature. 2015;526: 380–384. 10.1038/nature14905 26469049

[108] Wible JR, Hopson JA. Basicranial evidence for early mammal phylogeny In: Szalay FS, Novacek MJ, McKenna MC, editors. Mammal phylogeny: Mesozoic differentiation, multituberculates, monotremes, early therians, and marsupials. New York: Springer; 1993 pp. 45–62.

[109] Vater M, Meng J, Fox RC. Hearing organ evolution and specialization: Early and later mammals In: Manley GA, Popper AN, Fay RR, editors. Evolution of the vertebrate auditory system. New York: Springer; 2004 pp. 256–288.

[110] Maier W, Ruf I. Evolution of the mammalian middle ear: A historical review. J Anat. 2016;228: 270–283. 10.1111/joa.12379 26397963 PMC4718169

[111] Gauthier J, Kluge AG, Rowe T. Amniote phylogeny and the importance of fossils. Cladistics. 1988;4: 105–209. 10.1111/j.1096-0031.1988.tb00514.x 34949076

[112] Zeller U. The morphogenesis of the fenestra rotunda in mammals In: Duncker HR, Fleischer G, editors. Functional morphology in vertebrates. Stuttgart: Fisher; 1985 pp. 153–157.

[113] Zeller U. Die ontogenese und morphologie der fenestra rotunda und des aqueductus cochleae von *Tupaia* und anderen säugern. Gegenbaurs morphol jahrb. 1985;131: 179–204. 4007452

[114] Ji Q, Luo ZX, Zhang X, Yuan CX, Xu L. Evolutionary development of the middle ear in Mesozoic therian mammals. Science. 2009;326: 278–281. 10.1126/science.1178501 19815774

[115] McKenna MC. Toward a phylogenetic classification of the Mammalia In: Luckett WP, Szalay FS, editors. Phylogeny of the primates: a multidisciplinary approach. New York: Plenum Press; 1975 pp. 21–46.

[116] Prothero DR. New Jurassic mammals from Como Bluff, Wyoming, and the interrelationships of non-tribosphenic Theria. Bull Am Mus Nat Hist. 1981;167: 281–317.

[117] Ladevèze S, Asher RJ, Sánchez-Villagra MR. Petrosal anatomy in the fossil mammal *Necrolestes*: Evidence for metatherian affinities and comparisons with the extant marsupial mole. J Anat. 2008;213: 686–697. 10.1111/j.1469-7580.2008.00985.x 19094184 PMC2666137

[118] Paez-Arango N. Dental and craniomandibular anatomy of Peligrotherium tropicalis: the evolutionary radiation of South American dryolestoid mammals. M.Sc Thesis, University of Louisville. 2008.

[119] Ravicz ME, Slama MC, Rosowski JJ. Middle-ear pressure gain and cochlear partition differential pressure in chinchilla. Hearing Res. 2010;263: 16–25. 10.1016/j.heares.2009.11.014 PMC2866808 19945521

[120] Meng J, Fox RC. Therian petrosals from the Oldman and Milk River Formations (Late Cretaceous), Alberta, Canada. J Vertebr Paleontol. 1995;15: 122–130.

[121] Allin EF. The auditory apparatus of advanced mammal-like reptiles and early mammals In: MacLean PD, Roth JJ, Roth EC, editors. The ecology and biology of mammal-like reptiles. Washington: Smithsonian Institution Press: 1986 pp. 283–294.

[122] Rougier GW, Sheth AS, Spurlin BK, Bolortsetseg M, Novacek MJ. Craniodental anatomy of a new Late Cretaceous multituberculate mammal from Udan Sayr, Mongolia. Acta Paleont Polonica. 2016;67: 197–248.

[123] Wang Y, Hu Y, Meng J, Li C. an ossified Meckel’s cartilage in two Cretaceous mammals and origin of the mammalian middle ear. Science. 2001;294: 357–361 10.1126/science.1063830 11598297

[124] Gao CL, Wilson GP, Luo ZX, Maga AM, Meng Q, Wang X. A new mammal skull from the Lower Cretaceous of China with implications for the evolution of obtuse-angled molars and ‘amphilestid’ eutriconodonts. Proc R Soc B. 2009; rspb20091014 10.1098/rspb.2009.1014 PMC2842676 19726475

[125] Trofimov BA. A new generic name *Gobiotheriodon* for a symmetrodontan mammal *Gobiodon* Trofimov, 1980. Acta Paleontol Pol. 1997;42: 496–496.

[126] Averianov AO. Early Cretaceous “symmetrodont” mammal Gobiotheriodon from Mongolia and the classification of “Symmetrodonta”. Acta Paleont Polonica. 2002;47: 705–716.

[127] Mason MJ. Structure and function of the mammalian middle ear II: inferring structure from function. J Anat. 2016;228: 300–312. 10.1111/joa.12316 26100915 PMC4718164

[128] Basch ML, Brown RM, Jen H, Groves AK. Where hearing starts: the development of the mammalian cochlea. J Anat. 2016;228: 233–254. 10.1111/joa.12314 26052920 PMC4718162

[129] Manley GA. Evidence for an active process and a cochlear amplifier in nonmammals. J Neurophys. 2001;86: 541–549. 10.1152/jn.2001.86.2.541 11495929

[130] Wangemann P. K+ cycling and the endocochlear potential. Hearing Res. 2002;165: 1–9. 10.1016/s0378-5955(02)00279-4 12031509

[131] Wangemann P. Supporting sensory transduction: cochlear fluid homeostasis and the endocochlear potential. J Phys. 2006;576: 11–21. 10.1113/jphysiol.2006.112888 PMC1995626 16857713

[132] Wantanabe Y, Nakashima T, Yanagita N. Venous communications of the cochlea after acute occlusion of the vein of the cochlear aqueduct. Arch Otorhinolaryngol. 1988;245: 340–343. 3248070 10.1007/BF00457990

[133] Shodo R, Hayatsu M, Koga D, Horii A, Ushiki T. Three-dimensional reconstruction of root cells and interdental cells in the inner ear by serial section scanning electron microscopy. Biomed Res. 2017;38: 239–248. 10.2220/biomedres.38.239 28794401

[134] Tan X, Pecka JL, Tang J, Okoruwa OE, Zhang Q, Beisel KW, et al From zebrafish to mammal: Functional evolution of prestin, the motor protein of cochlear outer hair cells. J Neurophys. 2010;105: 36–44. 10.1152/jn.00234.2010 PMC3023371 21047933

[135] Liu Z, Li GH, Huang JF, Murpohy RW, Shi P. Hearing aid for vertebrates via multiple episodic adaptive events on prestin genes. Molec Biol and Evol. 2012;29: 2187–2198. 22416033 10.1093/molbev/mss087

[136] Manley GA, Fuchs PA. Recent advances in comparative hearing. Hearing Res. 2011;273: 1–6. 10.1016/j.heares.2011.01.005 21236326

[137] Patuzzi R. Cochlear micromechanics and macromechanics In: Dallos P, Popper AN, Fay RR, editors. The Cochlea. New York: Springer; 1996 pp. 186–257.

[138] Fritzsch B, Pan N, Jahan I, Ducan JS, Kopecky BJ, Elliot KL, et al Evolution and development of the tetrapod auditory system: An organ of Corti centric perspective. Evol and Dev. 2013;15: 63–79. 10.1111/ede.12015 PMC3918746 23331918

[139] Ramanathan K, Michael TH, Jiang GJ, Hiel H, Fuchs PA. A molecular mechanism for electrical tuning of cochlear hair cells. Science. 1999;283: 215–217. 10.1126/science.283.5399.215 9880252

[140] Crompton AW, Luo ZX. Relationships of the Liassic mammals *Sinoconodon*, *Morganucodon oehleri*, and *Dinnetherium* In: Szalay FS, Novacek MJ, McKenna MC, editors. Mammal phylogeny: Mesozoic differentiation, multituberculates, monotremes, early therians, and marsupials. New York: Springer; 1993 pp.30–44.

[141] Forsman KA, Malmquist MG. Evidence for echolocation in the common shrew, *Sorex araneus*. J Zool. 1988;216: 655–662.

[142] Fettiplace R. Hair cell transduction, tuning, and synaptic transmission in the mammalian cochlea. Comp Physiol. 2017;7: 1197–1227. 10.1002/cphy.c160049 PMC5658794 28915323

[143] Manoussaki D, Chadwick RS, Ketten DR, Arruda J, Dimitriadis EK, O’Malley JT. The influence of cochlear shape on low-frequency hearing. Proc Nat Acad Sci. 2008;105: 6162–6166. 10.1073/pnas.0710037105 18413615 PMC2299218

[144] Sellick PM, Patuzzi R, Johnstone BM. Measurement of basilar membrane motion in the guinea pig using the Mössbauer technique. J Acoust Soc Am. 1982;72: 131–141. 10.1121/1.387996 7108035

[145] Ruggero MA. Responses to sound of the basilar membrane of the mammalian cochlea. Curr Opin Neurobiol. 1992;2: 449–456. 1525542 10.1016/0959-4388(92)90179-o PMC3579517

[146] Henson MM, Henson OW Jr. Tension fibroblasts and the connective tissue matrix of the spiral ligament. Hearing Res. 1988;35: 237–258. 10.1016/0378-5955(88)90121-9 3143706

[147] Thomas JA, Jalili MS. Echolocation in insectivores and rodents In: Thomas JA, Moss CF, Vater M, editors. Echolocation in bats and dolphins. Chicago: University of Chicago Press; 2004 pp. 547–564.

[148] Müller MW, Wess FP, Bruns V. Cochlear place-frequency map in the marsupial *Monodelphis domestica*. Hear Res. 1993;67: 198–202. 8340271 10.1016/0378-5955(93)90247-x

[149] Ravizza RJ, Heffner HE, Masterson B. Hearing in primitive mammals: I. Opossum (*Didelphis virginianus*). J Audit Res. 1969;9: 1–7.

[150] Heffner HE, Ravizza RJ, Masterson B. hearing in primitive mammals III. Tree shrew (*Tupaia glis*). J Audit Res. 1969;9: 12–18.

[151] Mills DM, Shepherd RK. Distortion product otoacoustic emission and auditory brainstem responses in the echidna (*Tachyglossus aculeatus*). J Ass Res Otolaryngol. 2001;2: 130–146. 11550523 10.1007/s101620010059 PMC3201180

[152] Gates RG, Saunders JC, Bock GR. Peripheral auditory function in the platypus, *Ornithorhynchus anatinus*. J Acoustic Soc Am. 1974;56: 152–156. 10.1121/1.1903246 4853586

[153] Drexl M, Faulstich M, von Stebut B, Radtke-Schuller S, Kössl M. Distortion product otoacoustic emissions and auditory evoked potentials in the hedgehog tenrec, *Echinops telfairi*. J Ass Re Otolaryngol. 2003;4: 555–564. 10.1007/s10162-002-3043-5 PMC3202739 14569428

[154] Aitkin L. The auditory neurobiology of marsupials: a review. Hear Res. 1995;82: 257–266. 7775290 10.1016/0378-5955(94)00182-p

[155] Köppl C. Phase locking to high frequencies in the auditory nerve and cochlear nucleus magnocellularis of the barn owl, *Tyto alba*. J Neurosci. 1997;17: 3312–3321. 9096164 10.1523/JNEUROSCI.17-09-03312.1997 PMC6573645

[156] Arch VS, Grafe TU, Gridi-Papp M, Narins PM. Pure ultrasonic communication in an endemic Bornean frog. PLOS One. 2009; 10.1371/journal.pone.0005413 19401782 PMC2671607

[157] McKenna MC, Kielan-Jaworowska, Meng J. Earliest eutherian mammal skull from the Late Cretaceous [Coniacian] of Uzbekistan. Acta Palaeontol Pol. 2000;45: 1–54.

[158] O’Leary MA, Bloch JI, Flynn JJ, Gaudin TJ, Giallombardo A, Giannini NP, et al The placental mammal ancestor and the post-K-Pg radiation of placentals. Sci. 2013;339: 662–667. 10.1126/science.1229237 23393258

[159] Payan P, Borelli G, Priouzeau F, De Pontual H, Boeuf G, Mayer-Gostan N. Otolith growth in trout Oncorhynchus mykiss: supply of Ca^2+^ and Sr^2+^ to the saccular endolymph. J exp Biol. 2002;202: 2687–2695. 10.1242/jeb.205.17.2687 12151374

[160] Preston RE, Johnsson LG, Hill JH, Schacht J. Incorporation of radioactive calcium into otolithic membranes and middle ear ossicles of the gerbil. Acta Otolaryngologica. 1975;80: 269–275. 10.3109/00016487509121327 1180040

[161] Salt AN, Mleichar I, Thalmann R. Mechanisms of endocochlear potential generation by stria vascularis. The Laryngoscope. 1987;97: 984–991. 3613802

[162] Salt AN, Inamura N, Thalmann R, Vora A. Calcium gradients in inner ear endolymph. Am J Otolaryngol. 1989;10: 371–375. 2596623 10.1016/0196-0709(89)90030-6

[163] Fox RC, Meng J. An x-radiographic and SEM study of the osseus inner ear of multituberculates and monotremes (Mammalia): Implications for mammalian phylogeny and evolution of hearing. Zool J Linn Soc. 1997;121: 249–291.

[164] Bi S, Zheng X, Wang X, Cignetti NE, Yang S, Wible JR. An Early Cretaceous eutherian and the placental-marsupial dichotomy. Nature. 2018;558: 390–395. 10.1038/s41586-018-0210-3 29899454

[165] Allin EF, Hopson JA. Evolution of the auditory system in Synapsida (“mammal-like reptiles” and primitive mammals) as seen in the fossil record In: Webster DB, Popper AN, Fay RR, editors. The evolutionary biology of hearing. New York: Springer: 1992 pp. 587–614.

[166] Rougier GW, Apesteguίa S, Gaetano LC. Highly specialized skulls from the Late Cretaceous of South America. Nature. 2011;479: 98–102. 10.1038/nature10591 22051679

[167] Christensen-Dalsgaard J, Manley GA. Directionality of the lizard ear. J Exp Biol. 2005;208: 1209–1217. 10.1242/jeb.01511 15767319

[168] Nothwang HG. Evolution of mammalian sound localization circuits: a developmental perspective. Prog Neurobiol. 2016;141: 1–24. 10.1016/j.pneurobio.2016.02.003 27032475

[169] Christensen-Dalsgaard J, Manley GA. Acoustical coupling of lizard eardrums. J Ass Res Otolaryngol. 2008;9: 407–416. 18648878 10.1007/s10162-008-0130-2 PMC2580811

[170] Köppl C. Evolution of sound localisation in land vertebrates. Curr Biol. 2009;19: 635–639. 10.1016/j.cub.2009.05.035 19674542

[171] Kemp TS. Non-mammalian synapsids: the beginning of the mammal line In: Clack JA, Fay RR, Popper AN, editors. Evolution of the vertebrate ear: Evidence from the fossil record. Cham: Springer; 2016 pp. 107–137.

[172] Mason MJ. Internally coupled ears in living mammals. Biol Cybern. 2016;110: 345–358. 10.1007/s00422-015-0675-1 26794500 PMC5107206

[173] Grothe B, Pecka M. The natural history of sound localization in mammals-a story of neuronal inhibition. Frontiers in neural circuits. 2014;8: 1–19. 25324726 10.3389/fncir.2014.00116 PMC4181121

[174] Segall W. Morphological parallelisms of the bulla and auditory ossicles in some insectivores and marsupials. Fieldiana Zool. 1970;51: 169–206.

[175] Mason MJ. Structure and function of the mammalian middle ear I: Large middle ears in small desert mammals. J Anat. 2016;228: 284–299. 10.1111/joa.12313 26104342 PMC4718170

[176] Christensen-Dalsgaard J. Vertebrate pressure-gradient receivers. Hearing Res. 2011;273: 37–45. 10.1016/j.heares.2010.08.007 20727396

[177] Grothe B. The evolution of temporal processing in the medial superior olive, an auditory brainstem structure. Progress in neurobiology. 2000;61: 581–610. 10775798 10.1016/s0301-0082(99)00068-4

[178] Brown CH, May BJ. Comparative mammalian sound localization In: Popper AN, Fay RR, editors. Sound source localization. New York: Springer: 2005 pp.124–178.

[179] Rice JJ, May BJ, Spirou GA, Young ED. Pinna-based spectral cues for sound localization in cat. Hearing Res. 1992;58: 132–152. 10.1016/0378-5955(92)90123-5 1568936

[180] Gaetano LC, Rougier GW. New materials of *Argentoconodon fariasorum* (Mammaliaformes, Triconodontidae) from the Jurassic of Argentina and its bearing on triconodont phylogeny. J Vertebr Paleontol. 2011;31: 829–843.

[181] Luo ZX, Yuan CX, Meng QJ, Ji Q. A Jurassic eutherian mammal and divergence of marsupials and placentals. Nature. 2011;476: 442–445. 10.1038/nature10291 21866158

[182] Cantos R, Cole LK, Acampora D, Simeone A, Wu DK. Patterning of the mammalian cochlea. Proc Natl Acad Sci. 2000;97: 707–711. 10.1073/pnas.97.22.11707 PMC34339 11050199

[183] Bok J, Chang W, Wu DK. Patterning and morphogenesis of the vertebrae inner ear. Int J Dev Biol. 2007;51: 521–533. 10.1387/ijdb.072381jb 17891714

[184] Luo Z. Developmental patterns in Mesozoic evolution of mammal ears. Annu Rev Ecol Evol Syst. 2011;42: 355–380.

[185] Polley DB, Seidl AH, Wang Y, Sanchez JT. Chapter 2—Functional circuit development in the auditory system In: Rubenstein JLR, Rakic P, editors. Neural circuit development and function in the brain. Oxford: Academic Press; 2013 pp 21–39.

[186] Crompton AW. The origin of the tribosphenic molar. Zool J Linn Soc. 1971;50: 65–81.

[187] Wilson GP. Mammals across the K/Pg boundary in northeastern Montana, USA: dental morphology and body-size pattens reveal extinction selectivity and immigrant-fueled ecospace filling. Paleobiol. 2013;39: 429–469.

[188] Schultz JA, Martin T. Function of pretribosphenic and tribosphenic mammalian molars inferred from 3D animation. Naturwissenschaften. 2014;101: 771–781. 10.1007/s00114-014-1214-y 25091547

[189] Grossnickle DM, Newham E. Therian mammals experience an ecomorphological radiation during the Late Cretaceous and selective extinction at the K-Pg boundary. Proc R Soc B. 2016;283: 1–8.

[190] Grossnickle DM. The evolutionary origin of jaw yaw in mammals. Sci Rep. 2017;7: 1–13. 28322334 10.1038/srep45094 PMC5359619

[191] Gu JJ, Montealegre-Z F, Robert D, Engel MS, Qiao GX, Ren D. Wing stridulation in a Jurassic katydid (Insecta, Orthoptera) produced low-pitched musical calls to attract females. Proc Natl Acad Sci. 2012;109: 3868–3873. 10.1073/pnas.1118372109 22315416 PMC3309752

[192] Close RA, Friedman M, Graeme TL, Benson RBJ. Evidence for a Mid-Jurassic adaptive radiation in mammals. Curr Biol. 2015;25: 2137–2142. 10.1016/j.cub.2015.06.047 26190074

