# Supplementary material for: Petrosal morphology and cochlear function in Mesozoic stem therians
Source: PLoS One. 2019 Aug 14;14(8):e0209457. doi: 10.1371/journal.pone.0209457 (PMC6693738; doi:10.1371/journal.pone.0209457)
Supplement: S1 File — Phylogenetic and anatomical background: the premammalian ear region. (DOCX) [file pone.0209457.s001.docx]

**Evolution of the Synapsid Ear**

Synapsid fossils have a unique significance for the study of the nervous system [1-3]. In particular, skeletal structures such as the mammalian petrosal and middle ear show an especially close relationship with the central and peripheral nervous tissues that utilize them. The fossil record also provides otherwise unattainable information on the sequence and timing of the evolution of these tissues. As emphasize in [3], the modern mammalian nervous system is the product of many consecutive episodes of reorganization, many of which are best studied in the cranial remains of fossil synapsids. This synopsis of the stem-based clade Pan-Mammalia (inclusive of all taxa more closely related to mammals than any other living organism), by the characterization of successively more exclusive clades based around the therian crown group therefore provides a useful focus and phylogenetic context for understanding neurosensory evolution within this lineage, and the significance of stem therian petrosal structure within it.

From the earliest stretch of stem therian evolution, several extinct but derived groups appear to have diverged [4,5]. These groups include the questionably paraphyletic eutriconodonts [6-9], the multituberculates ([10-14]; along with their probable sister taxon Gondwannatheria [15]), and the spalacotheroid symmetrodonts [16,17]. The high-resolution micro-CT images used in this report represent the first observations of the internal structure of the otic capsule in eutriconodonts and possibly “symmetrodont” mammals. Because of the highly derived apomorphies seen in even the earliest representatives of the multituberculate lineage, such as the presence of multiple “foramina ovale” (apertures for the mandibular branch of the trigeminal nerve; [11]), and even more morphologically unique features found in later members of this lineage [10], the diversity of mutituberculate petrosal morphology is considered a specialized side branch of early mammals and is not discussed further here.

Aside from contributing to the body of anatomical detail known for these obscure stem therian groups, petrosal descriptions allow for the broad reconstruction of auditory sensitivity, selectivity, and range in the members of the stem therian lineage. These reconstructions require a wide perspective on petrosal diversity, however, and input from several independent research programs; particularly 1) biomechanical analyses of earlier synapsid fossils [18,19]; 2) comparative and developmental studies of therian and cladotherian anatomy [20,21]; and 3) physiological research on modern mammals and non-mammalian amniotes [22,23]. As outlined below, the reconstruction of which of three non-mutually-exclusive forms of cochlear tuning, or which of four forms of sound localization, were likely present in the stem therians described here will critically depend on the findings of prior analyses of auditory physiology and vascular anatomy in non-mammalian amniotes and the construction and distribution of middle ears and their anatomically precedent structures [24-27]. The following discussion therefore parallels [3] in tracing a series of sequentially more recent nodes along the backbone of synapsid phylogeny, beginning with the characterization of the amniote common ancestor and ending with the mammalian crown group (which is picked up in the main text; see SI Fig 1).

**
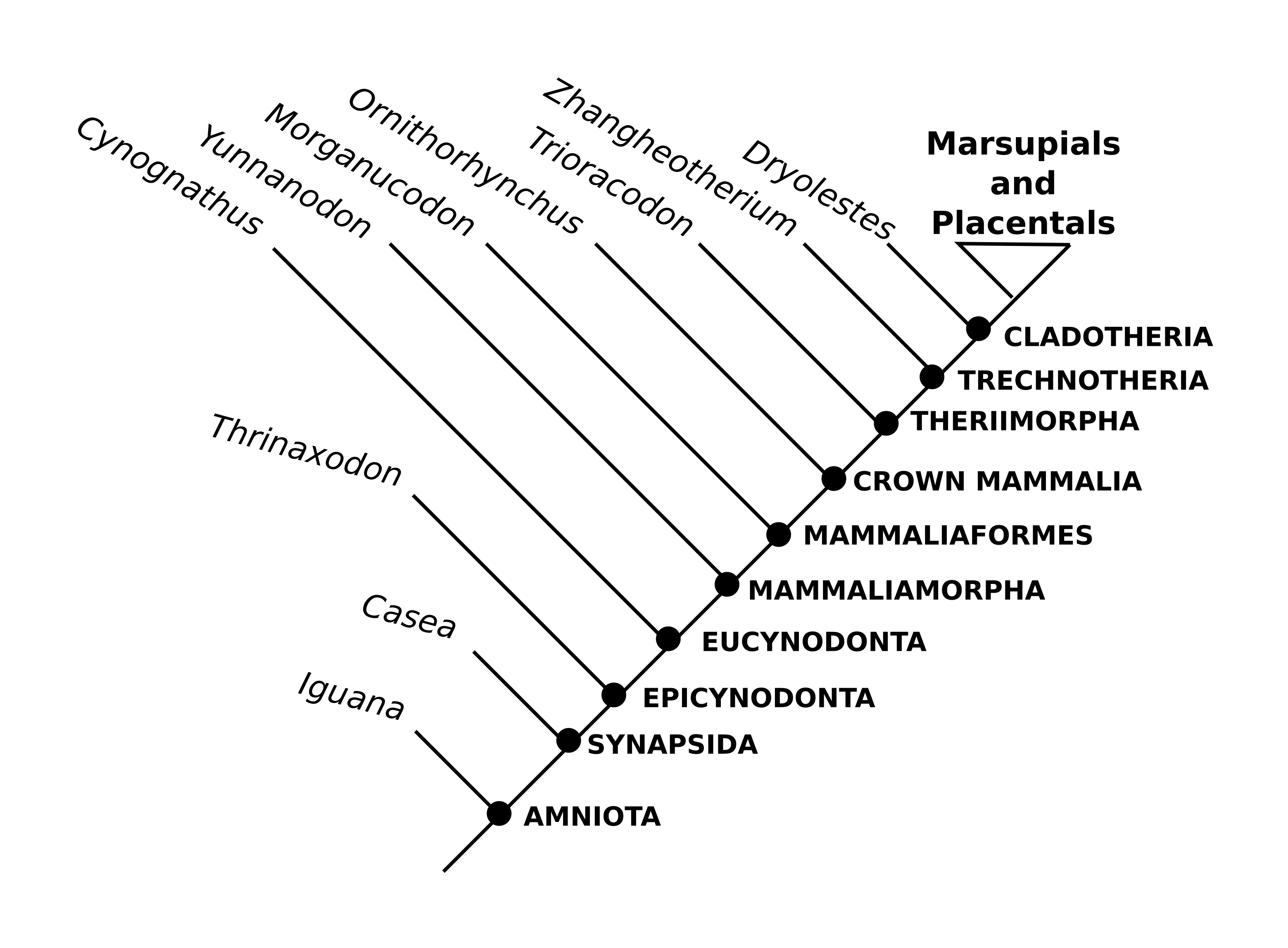
**

**Fig S1. Example cladogram showing consecutively nested clades referred to in text.**

**Amniotes**

The late Paleozoic ancestors of the modern amniote lineages (Sauropsida and Synapsida) are reconstructed as petite “reptiles” with an estimated nine-inch snout-vent length [28]. The anteroventral subdivision of the endolymphatic labyrinth in these forms is termed the pars inferior, initially a minor diverticulum accommodating three specialized sensory epithelia [29,30]. These sensory epithelia include the incipiently subdivided saccular and lagenar maculae, and the basilar papilla (an even more recently acquired extension of the saccule; [2]). While the sensory modalities and performance parameters within which these epithelia operate are considerably more complex in fishes and amphibians, for extant and fossil members of the amniote lineage it is most likely that the saccular macula took on a dedicated role in equilibrium and proprioception, the basilar papilla an increasingly specialized role in sound perception, and the lagenar macula some combination of the two roles which became progressively more redundant thought out synapsid history [2]. The sensory epithelia contained within the pars inferior were in turn accommodated by a matching concavity within the bony labyrinth termed the sacculocochlear recess (i.e. the lagenar recess [29,31], inter alios). This configuration allowed the membrane supporting the basilar papilla to oscillate in response to vibrations induced within the bony labyrinth [32]. The reconstructed lack of a coherent gradient in width, thickness, and material properties in the early amniotic basilar membrane (a condition retained in most extant sauropsids) constrained the entire basilar membrane to oscillate homogeneously in response to the frequency content of external stimuli. In early amniotes (including early synapsids) the fenestra vestibuli was also located at the lateral or ventrolateral margin of the sacculocochlear recess.

The approximately 1 mm long basilar papilla (homolog to the mammalian organ of Corti) in the amniote common ancestor is estimated to have been competent for only a 3 or 4 octave interval of detectable frequencies; corresponding to an equally diminutive acoustic Space Constant (SC, or Space Per Octave) of ~0.3 mm per octave [33-37]. This short theoretical SC, together with the reconstructed consignment of these animals to the low-frequency range, is based on physiological observations of extant sauropsids (*Sphenodon* and turtles), many of which retain the most plesiomorphic form of frequency selectivity, termed electrical tuning [38].

Electrical tuning involves the active and intrinsic calibration of each individual auditory hair cell by the modulation of the density and kinetics of voltage-gated calcium channels in the basolateral cell membrane [39,40]. Calcium is critical for allowing the release of cytosolic potassium in a depolarized hair cell, and therefore its subsequent hyperpolarization. Because of the considerable refractory period in which an individual hair cell is required to import potassium from the endolymph, depolarize, import calcium from the basolateral membrane, export potassium through the basolateral membrane and thus repolarize - the intrinsic tuning of these auditory hair cells can only correspond to a dynamic range of frequencies starting from less than 100 Hz to around 1 kHz maximally in most ectotherms [34]. At the heightened body temperatures of endotherms, theoretical considerations suggest that electrical tuning would be functional up to approximately 4 kHz [41,42].

The likely electrically tuned basilar papilla within the first amniotes was metabolically supported by both vascular and epithelial tissues running throughout the pars inferior, especially the basilar membrane (the “bottom” of the reptilian cochlear duct) and Reissner’s membrane (also termed the vestibular membrane; which forms the “top” of the cochlear duct). The tortuous capillary plexus within Reissner’s membrane in particular is homologous to the original and sole endolymph-producing organ in the ancestral amniotes, and is today retained in all extant sauropsids as well as monotremes (Fig 14a,b ;[43]). The initial configuration of blood vessels supporting the auditory apparatus therefore encircled the pars inferior with little to no integration of these vessels with the surrounding skeleton of the otic capsule. Indeed, one of the main observations made during resections of the auditory apparatus of non-mammalian amniotes is that the cochlear duct can be “scooped” cleanly with dissecting tools from its cartilaginous or bony housing in the otic capsule [44,22]. This lack of bony integration is most dramatically apparent in birds, where the hypertrophied capillary plexus in the vestibular membrane is termed the tegmentum vasculosum (e.g. in [44] fig 5 section 1; this reference also shows the source of this vasculature to be endocranial, penetrating the otic capsule in proximity with the cochlear nerve). The vascularized membrane, separating the endolymphatic labyrinth from the perilymphatic space, also supports the lagenar macula and its attached otoconial mass in extant sauropsids and monotremes [43,30]. Thus, it can be inferred that the production and composition of endolymph in modern sauropsids, with its characteristically high (~ 30 micromolar to over 1 millimolar; [45]) concentration of free calcium cations and low electrical potential, was initiated in the earliest amniote ancestors possibly as a means of facilitating the electrical tuning of the basilar papilla and stabilizing the lagena and other otoconial masses [46].

**Synapsids**

There is a consensus that forms in the early burst of synapsid evolution lacked any form of tympanic membrane or acoustically relevant cranial air-spaces [22,47]. Additionally, the co-option of the hypertrophied stapes (a second arch viscerocranial element) as a firm structural interconnection between the posterior neurocranial and dermatocranial components of the skull, acted as a mechanism for reinforcing the ancestral jaw apparatus against structural deflections during biting. This probably also limited its auditory sensitivity [48,49,22].

What acoustic receptivity these forms achieved was likely mediated through a mixture of indirect conduction (where the majority of the dermatocranium acted as a vibrational antenna, possibly in service of a semiaquatic lifestyle in many taxa) and direct conduction (where seismic/airborne vibrations are transmitted through the lower jaw [19,50]). Waves of pressure were therefore transmitted into the otic capsule through the skull’s out-of-phase motion with respect to the massive and inertially stabilized stapes [47,51]. These conduction mechanisms are most appropriate for low-frequency and high-amplitude vibrations and electrical tuning would have been more than capable of processing these signals.

Opposite to the condition of its lateral border, the endocranial wall of the otic capsule is poorly ossified in pelycosaurs and most other non-mammaliaform synapsids. This is reflected by the absence of an invaginated internal acoustic meatus and lack of ossified partitions separating the perilymphatic foramen and cranial nerves VII-XII along the medial aspect of the braincase [52]. What can be determined about the structure of the otic capsule shows that the pars cochlearis is absent in the earliest synapsids; however, the corresponding pars inferior of the endolymphatic labyrinth would have been accommodated by a shallow sacculocochlear recess that itself did not emarginate the wider contour of the bony labyrinth [32]. The lack of a pars cochlearis within the early synapsid otic capsule does not imply a more or less monolithic composition of the periotic skeleton, however; and the adult otic capsule in early synapsids is a composite structure mainly formed by the prootic and opisthotic bones, with variable minor contributions from other cranial elements [47].

By the Middle Permian a diverse group of therapsids had evolved from the carnivorous sphenacodontan pelycosaurs. These animals display a trend of progressive loosening of the quadrojugal and quadrate from the squamosal, possibly resulting in some combination of incipient streptostyly and whole-bone sound conduction. Additionally, the increased development of the reflected lamina of the angular bone (homolog of the mammalian ectotympanic) and formation of the recessus mandibularis as a mandibular resonating chamber, suggest that direct conduction of low-frequency, possibly airborne, sound was possible. The relatively voluminous recessus mandibularis in some of the larger therapsids point to its possible dual function as a resonator for both the reception and production of airborne vocal signals [49]. With the increased development of the recessus mandibularis, a trend of decreasing reliance on sound conduction via intra-bone vibrations and greater reliance on conduction through whole-bone vibration is also likely [47]. However, the low transformation ratios between the area of the reflected lamina of the angular and the fenestra vestibuli would have provided little compensation for the energy lost due to impedance mismatch between the surrounding air and fluids inside the otic capsule [53,18,49].

**Epicynodonts**

Near the Permo-Triassic boundary the epicynodonts, along with other therapsid groups, show the complete subdivision of the oronasal cavity into dedicated oral and nasal cavities by the completion of an osseous secondary palate [54,55]. The resultant caudal aperture of the nasal cavity, the internal choanae, created a new communication to the rostral pharynx (nasopharynx). The nasopharynx itself became a hub for gaseous communication to the larynx caudally, and its lateral apertures are also reconstructed as having communicated with the recessus mandibularis [56]. If this is the case, it constitutes the first well-delimited connection between the nasopharynx and the ear region.

The epicynodonts also show the subdivision of the sacculocochlear recess of the bony labyrinth into a dedicated saccular recess (recessus sphericus) and a separate bony cul-de-sac termed the cochlear recess [32]. This morphology of the bony labyrinth suggests that the corresponding pars inferior of the membranous labyrinth was also at least incipiently subdivided into a discrete saccule (confluent at one end to the endolymphatic duct and utricle, and the cochlear duct at the opposite end) and cochlear duct. In this transitional cochlear organization (as demonstrated by *Thrinaxodon*; [57]) the rostral border of the ventromedially pointing cochlear recess within the prootic bone does not extend anterior to the rostral border of the fenestra ovalis. As such, these forms lack a true pars cochlearis of the prootic bone [32].

**Eucynodonts**

Eucynodonts show a progressive trend of increasing relative size and differentiation of the jaw adductor musculature, while simultaneously reducing the size, strength, and cranial integration of the bony jaw apparatus itself. This counter-intuitive development was permitted by the reorientation of adductor leverage to minimize the reaction force experienced by the quadrate-articular joint and postdentary elements [58]. Other morphological features seen in basal forms of this taxon include the change from a plate-like reflected lamina to a rod-like reflected lamina of the angular bone, creating a laterally facing gap between the reflected lamina and main body of the angular, that was almost certainly spanned in life by an incipient tympanic membrane [54]. The remaining postdentary bones were synostosed into a gracile but rigid postdentary rod that remained attached to the angular. As the postdentary rod, the postdentary bones lost their sutural connections with the dentary bone but remained appressed to the dentary within a smoothly concave postdentary trough [59]. The rostrocaudal orientation of the postdentary rod, with its flexible posterior connection to the squamosal, and lack of strong rostral attachments to the dentary, allowed the postdentary rod to function as a first-class lever with a longitudinally oriented fulcral axis. This provided a mechanical linkage for transmitting airborne vibrations impinging on the angular tympanic membrane onto the quadrate and stapes medially [47]. The air-filled space medial to the tympanic membrane (likely a vestige of the recessus mandibularis), combined with the approximately 1/30 area ratio between the fenestra ovalis and angular tympanic membrane [19], would create a precursory condition to the middle ear seen in the later and most advanced cynodonts. However, the existence of a true cavum tympani medial to the angular tympanic membrane and the usefulness of this apparatus for frequencies above 2 kHz is doubtful [19]. The eucynodonts also show the first appearance of a true pars cochlearis. This neomorphic region is defined in [60] (also see [61,32]) as the portion of the otic capsule accommodating the saccule and cochlear duct, that were likely present as discrete structures in epicynodonts. The weight of morphological evidence suggests that while not within crown mammalian performance levels, the auditory capacities of the early eucynodonts were heightened beyond anything preceding them in synapsid history [47].

**Mammaliamorphs**

Mammaliamorphs include the true mammaliaforms and several clades showing incredible morphological convergences with them [62]. This group also includes the only synapsid taxa to survive beyond the Early Cretaceous. These forms show many significant apomorphies related to the function of their inner ear and newly acquired middle ear - including the consolidation of the prootic and opisthotic into the true petrosal bone [54,32], the division of the common jugular fossa into a perilymphatic foramen and a jugular foramen, and separation of the foramina for the cochlear and vestibular branches of the vestibulocochlear nerve [63]. However, far and away the most lauded morphological characteristics seen in this group are the articulations between the dentary and squamosal bone, and development of a true cavum tympani [51,64]. The development of a tympanic middle ear in synapsid ancestry is an important event with functional implications regarding the sensitivity and maximal frequency attained by these and later forms; however, the accurate interpretation of these features, especially the contents of the otic capsule, rely on comparative inferences derived from the study of the tympanic middle ears in fossils and extant non-mammalian amniotes. It should therefore be reiterated here that, (for anatomical and developmental criteria skillfully outlined in [65,66,27] inter alios) the best interpretation of the available evidence suggests that the acoustically significant intracranial air spaces developed in advanced synapsids are not homologous with tympanic cavities developed in many other tetrapod lineages [22]. The initial form of the synapsid cavum tympani likely displayed a broadly confluent relationship with the nasopharynx. The medial (proximal) subdivision of the cavum tympani was therefore not separated from the nasopharynx by an extended auditory tube and likely allowed bulk motion of air between the lateral portion of the tympanic cavity to the nasopharynx and onward across to the contralateral cavum tympani. Modern *Ornithorhynchus* also shows this condition [67].

An unconstricted (if tortuous) air-filled passageway connecting both ears is hypothesized as a mechanism for sound localization in advanced cynodonts and other, more plesiomorphic synapsids [68]. In the majority of extant sauropsids the sole method of sound localization in the horizontal (azimuthal) plane is by the simultaneous use of the ipsilateral and contralateral ears, and the air-mass medially connecting both, as a pressure-gradient receiver [22,69-72]. The successive medial-posterior-ventral relocation of the articular and quadrate relative to the newly formed dentary squamosal contact, likely made the contact between both ears progressively more constricted in the advanced cynodonts.

The degree of ossification of the otic capsule in Mammaliamorpha is also significantly greater than in other known cynodonts, with two discrete foramina for the vestibular and cochlear branches of the vestibulocochlear nerve, respectively. These are recognizable in the medial wall of the petrosal even in early forms [73,64], and in the most plesiomorphic condition they are of subequal diameter. These forms are also the first to show an appreciable amount of “waisting” between the components of the vestibular endocast contributed by the pars cochlearis and pars canalicularis. The development of a more or less prominent crista vestibuli also separates the areas of attachment for the utricular and saccular maculae, forming discrete vestibular recesses to accommodate these structures (the recessus ellipticus and recessus sphericus, respectively; [20,64]).

**Mammaliaformes**

Mammaliaformes includes Mammalia and their common ancestor with the Triassic-Jurassic *Morganucodon*, plus all of its descendants. Having inherited a mandibular middle-ear from advanced mammaliamorph cynodonts, the Mammaliaformes reinforce the dentary-squamosal jaw articulation by developing a true mandibular condyle on the dentary, matching an equally developed glenoid fossa on the squamosal [62,4]. However, with the progressive reduction of the crista parotica on the petrosal and redistribution of masticatory musculature onto the dentary bone, the placement and size of the ancestral jaw articulation became more directly under the influence of the auditory mechanism, while the dentary and squamosal became increasingly specialized for masticatory purposes [74,48, 58,4].

Along with the increasing specialization of the articular and quadrate for acoustic purposes, a wider reorganization of external and internal aspects of the petrosal can also be seen in early mammaliaform fossils. Externally, the enlargement of the pars cochlearis at the expense of the basisphenoid and other midline structures and loss of a thickened rim around the fenestra ovalis, and the separation of the hypoglossal and jugular foramina [74, 33] can be seen in early members of the clade. These features are possibly related to the insulation and stabilization of structures surrounding the otic capsule, or in the case of the reduced rim of the fenestra vestibuli to facilitate a greater range of motion between the stapes and petrosal (i.e. a “rocking”-type motion between the stapes and petrosal, in addition to a piston like motion; [65]). More significantly, these mammaliaforms also show the allometric enlargement and ventral inflation of the pars cochlearis of the petrosal relative to other advanced cynodonts, forming a variably flattened or convex promontorium on its newly exposed ventral surface. A promontorial structure has also been observed in juvenile non-mammaliaform probainognathians [75], and as [52] observed, at the time of its initial appearance the majority of the increased volume in the pars cochlearis is not immediately utilized by the elongated but relatively small cochlear canal. Instead, the persistence of the promontorium into adult stages in mammaliaforms and other mammalimorph taxa may solely be a product of paedomorphosis, as in these early forms most of the volume of the promontorium is taken up by a complex network of venous canals and sinuses termed the circumpromontorial sinus plexus [76-78].

In some mammaliaformes the circumpromontorial sinus plexus includes two sets of venous canals dorsal and ventral to the cochlear canal accommodating the epicochlear and hypocochlear sinuses, that communicate with the inferior petrosal sinus. The inferior petrosal sinus is also located in an intramural location between the petrosal and basisphenoid in these early taxa [79]. The ventral bulging of the pars cochlearis relative to the cranial base also variably impressed onto the stapedial artery, forming an indentation along the margins of the fenestra ovalis in *Morganucodon* and more advanced forms [63]. Endocranially, the earliest mammalaiforms still lacked an excavated internal acoustic meatus, with the small prefacial commissure showing little association with the course of the vestibulocochlear nerve [76]. As mentioned in the descriptions of the Höövör petrosals in the main text, it is also very likely that a posterior epicochlear sinus, running between the inferior petrosal sinus and prootic sinus, was present within the bone separating the primary facial foramen and the foramen for the vestibular branch of the vestibulocochlear nerve. Other significant features of the pars cochlearis are internal, such as the initiation of the abneural curvature (concave toward the side of insertion of the cochlear nerve) of the cochlear canal and, as seen in *Morganucodon* and more derived stem mammaliaforms, the relative inflation of the apical cochlear canal for the accommodation of the lagenar macula [80].

**References**

1. MacLean PD. Neurobehavioral significance of the mammal-like reptiles (theriapsids). In: MacLean PD, Roth JJ, Roth EC, editors. The ecology and biology of mammal-like reptiles. Washington: Smithsonian Institution Press: 1986. pp. 1-21.

2. Butler AB, Hodos W. Comparative vertebrate neuroanatomy: Evolution and adaptation. 2nd ed. Hoboken: John Wiley and Sons Inc.; 2005.

3. Rowe TB. The emergence of mammals. In: Kaas JH, editor. Evolution of nervous systems, second edition, volume 2. Oxford: Elsevier; 2017. pp. 1-52.

4. Kielan-Jaworowska Z, Cifelli RL, Luo ZX. Mammals from the age of dinosaurs: Origins, evolution, and structure. New York: University of Colombia Press; 2004.

5. Kielan-Jaworowska, Z. In pursuit of early mammals. Bloomington: Indiana University Press; 2013.

6. Kermack KA. The cranial structure of the triconodonts. Phil Trans R Soc Lond B. 1963;246: 83-103.

7. Meng J, Hu Y, Wang Y, Li C. The ossified Meckel’s cartilage and internal groove in Mesozoic mammaliaformes: implications to origin of the definitive mammalian middle ear. Zool J Linn Soc. 2003;138: 431-448.

8. Gaetano LC, Rougier GW. New materials of Argentoconodon fariasorum (Mammaliaformes, Triconodontidae) from the Jurassic of Argentina and its bearing on triconodont phylogeny. J Vertebr Paleontol. 2011;31: 829-843.

9. Gaetano LC, Rougier GW. First amphilestid from South America: a molariform from the Jurassic Cañadón Asfalto Formation, Patagonia, Argentina. J Mammal Evol. 2012;19: 235-248.

10. Kielan-Jaworowska Z, Presley R, Poplin C. The cranial vasculature system in taeniolabidoid multituberculate mammals. Phil Trans R Soc Lond. 1986;313: 525-602.

11. Hahn G. Die ohr-region der Paulchoffatiidae (Multituberculata, Ober-Jura). Palaeovertebrata. 1988;18: 155-185.

12. Luo Z, Ketten DR. CT scanning and computerized reconstructions of the inner ear of multituberculate mammals. J Vertebr Paleontol. 1991;11: 220-228.

13. Meng J, Wyss AR. Monotreme affinities and low-frequency hearing suggested by multituberculate ear. Nature. 1995;377: 141-144.

14. Fox RC, Meng J. An x-radiographic and SEM study of the osseus inner ear of multituberculates and monotremes (Mammalia): Implications for mammalian phylogeny and evolution of hearing. Zool J Linn Soc. 1997;121: 249-291.

15. Hoffmann S, O’Connor PM, Kirk EC, Wible JR, Krause DW. Endocranial and inner ear morphology of *Vintana sertichi* (Mammalia, Gondwanatheria) from the Late Cretaceous of Madagascar. J Vetebr Paleontol. 2014;34: 110-136.

16. Hu Y, Wang Y, Luo Z, Li C. A new symmetrodont mammal from China and its implications for mammalian evolution. Nature. 1997;390: 137-142.

17. Rougier GW, Ji Q, Novacek MJ. A new symmetrodont mammal with fur impressions from the Mesozoic of China. 2003;77: 7-14.

18. Fleischer G. Evolutionary principals of the mammalian middle ear. Adv Anat Embryol Cell Biol. 1978;55: 1-70.

19. Kemp TS. Acoustic transformer function of the postdentary bones and quadrate of a nonmammalian cynodont. J Vetebr Paleontol. 2007;27: 431-441.

20. Meng J, Fox RC. Osseus inner ear structures and hearing in early marsupials and placentals. Zool J Linn Soc. 1995;115: 47-71.

21. Basch ML, Brown RM, Jen H, Groves AK. Where hearing starts: the development of the mammalian cochlea. J Anat. 2016;228: 233-254.

22. Manley GA. An evolutionary perspective on middle ears. Hearing Res. 2010;263: 3-8.

23. Manley GA. The foundations of high-frequency hearing in early mammals. J Mammal Evol. 2016;25: 155-163.

24. Anthwal N, Joshi L, Tucker AS. Evolution of the mammalian middle ear and jaw: adaptations and novel structures. J Anat. 2013;222: 147-160.

25. Mason MJ. Of mice moles and guinea pigs: functional morphology of the middle ear in lining mammals. Hearing Res. 2013;301: 4-18.

26. Mason MJ. Structure and function of the mammalian middle ear II: inferring structure from function. J Anat. 2016;228: 300-312.

27. Maier W, Ruf I. Evolution of the mammalian middle ear: A historical review. J Anat. 2016;228: 270-283.

28. Laurin M. The evolution of body size, Cope’s rule and the origin of amniotes. Sys Biol. 2004;53: 594-622.

29. Fritzsch B, Pan N, Jahan I, Ducan JS, Kopecky BJ, Elliot KL, et al. Evolution and development of the tetrapod auditory system: An organ of Corti centric perspective. Evol and Dev. 2013;15: 63-79.

30. Schultz JA, Zeller U, Luo ZX. Inner ear labyrinth anatomy of monotremes and implications for mammalian inner ear evolution. J Morphol. 2017;278: 236-263.

31. Sigogneau D. The inner ear of *Gorgonops* (Reptilia, Therapsida, Gorgonopsia).

Ann S Afr Mus. 1974;64: 53-69.

32. Luo Z. The inner ear and its bony housing in tritylodontids and implications for evolution of the mammalian ear. Bull Mus Comp Zool Harvd. 2001;156: 81-97.

33. Clack JA. The evolution of tetrapod ears and the fossil record. Brain Behav Evol. 1997;50: 198-212.

34. Manley GA, Köppl C. Phylogenetic development of the cochlea and its innervation. Curr Opin Neurobiol. 1998;8: 468-474.

35. Manley GA. Travelling waves and tonotopicity in the inner ear: a historical and comparative perspective. J Comp Physiol A. 2018;204: 773-781.

36. Manley GA. Cochlear mechanisms from a phylogenetic viewpoint. Proc Natl Acad Sci. 2000;97: 11736-11734.

37. Manley GA. The foundations of high-frequency hearing in early mammals. J Mammal Evol. 2016;25: 155-163.

38. Ramanathan K, Michael TH, Jiang GJ, Hiel H, Fuchs PA. A molecular mechanism for electrical tuning of cochlear hair cells. Science. 1999;283: 215-217.

39. Fettiplace R, Fuchs PA. Mechanisms of hair cell tuning. Ann Rev Physiol. 1999;61: 809-834.

40. Fettiplace R. Hair cell transduction, tuning, and synaptic transmission in the mammalian cochlea. Comp Physiol. 2017;7: 1197-1227.

41. Wu YC, Art JJ, Goodman MB, Fettiplace R. A kinetic description of the calcium- activated potassium channel and its application to electrical tuning of hair cells. Prog Biophys Mol Biol. 1995;63: 131-158.

42. Manley GA. Comparative auditory neuroscience: Understanding the evolution and function of ears. J Assoc Res Otolaryngol. 2017;18: 1-24.

43. Wever EG. The reptile ear: Its structure and function. Princeton: Princeton University Press; 1978.

44. Hossler FE, Olson KR, Musil G, McKamey MI. Ultrastructure and blood supply of the tegmentum vasculosum in the cochlea of the duckling. Hearing Res. 2002;164: 155- 165.

45. Köppl C, Wilms V, Russell IJ, Nothwang HG. Evolution of endolymph secretion and endolymph potential generation in the vertebrate inner ear. Brain Behav Evol. 2018; DOI: 10.1159/000494050

46. Manley GA. The mammalian Cretaceous cochlear revolution. Hearing Res. 2017;352: 23-29.

47. Kemp TS. Non-mammalian synapsids: the beginning of the mammal line. In: Clack JA, Fay RR, Popper AN, editors. Evolution of the vertebrate ear: Evidence from the fossil record. Cham: Springer; 2016. pp. 107-137.

48. Kermack KA, Musset F, Rigney HW. The lower jaw of *Morganucodon*. Zool J Linn Soc. 1973;53: 87-175.

49. Allin EF. The auditory apparatus of advanced mammal-like reptiles and early mammals. In: MacLean PD, Roth JJ, Roth EC, editors. The ecology and biology of mammal-like reptiles. Washington: Smithsonian Institution Press: 1986. pp. 283-294.

50. Laaß M. The origins of the cochlea and impedance matching hearing in synapsids. Acta Paleontol Pol 2015;61: 267-281.

51. Luo ZX, Schultz JA, Ekdale EG. Evolution of the middle and inner ears of mammaliaformes: the approach to mammals. In: Clack JA, Fay RR, Popper AN, editors. Evolution of the vertebrate ear: Evidence from the fossil record. Cham: Springer; 2016. pp. 139-174.

52. Luo Z, Crompton AW, Lucas SG. Evolutionary origins of the mammalian promontorium and cochlea. J Vertebr Paleontol. 1995;15: 113-121.

53. Benoit J, Manger PR, Fernandez V, Rubidge BS. The bony labyrinth of late Permian Biarmosuchia: palaeobiology and diversity of non-mammalian Therapsida. Palaeont Afr. 2017;52: 58-77.

54. Olson EC. Relationships and ecology of the early therapsids and their predecessors. In: MacLean PD, Roth JJ, Roth EC, editors. The ecology and biology of mammal-like reptiles. Washington: Smithsonian Institution Press: 1986. pp. 47-60.

55. Ruta M, Botha-Brink J, Mitchell SA, Benton MJ. The radiation of cynodonts and the ground plan of mammalian morphological diversity. Proc R Soc B. 2013;280: 1-10.

56. Barghusen HR. On the evolutionary origin of the therian tensor veli palantini and tensor tympani muscles. In: MacLean PD, Roth JJ, Roth EC, editors. The ecology and biology of mammal-like reptiles. Washington: Smithsonian Institution Press: 1986. pp. 253-262.

57. Fourie S. The cranial morphology of *Thrinaxodon liorhinus* Seeley. Ann S Afr Mus. 1974;56: 337-400.

58. Crompton AW, Hylander WL. Changes in mandibular function following the acquisition of a dentary-squamosal jaw articulation. In: MacLean PD, Roth JJ, Roth EC, editors. The ecology and biology of mammal-like reptiles. Washington: Smithsonian Institution Press: 1986. pp. 175-282.

59. Allin EF, Hopson JA. Evolution of the auditory system in Synapsida (“mammal-like reptiles” and primitive mammals” as seen in the fossil record. In: Webster DB, Fay RR, Popper AN, editors. The evolutionary biology of hearing. Heidelberg: Springer-Verlag: 1992. pp. 587-614.

60. DeBeer GR. The development of the vertebrate skull. Oxford: Clarendon Press.; 1937

61. MacPhee RD. Auditory reigions of primates and eutherian insectivores. Basel: S. Karger; 1981.

62. Rowe TB. Definition, diagnosis, and the origin of Mammalia. J Vetebr Paleontol. 1988;8: 241-264.

63. Wible JR, Hopson JA. Basicranial evidence for early mammal phylogeny. In: Szalay FS, Novacek MJ, McKenna MC, editors. Mammal phylogeny: Mesozoic differentiation, multituberculates, monotremes, early therians, and marsupials. New York: Springer; 1993. pp. 45-62.

64. Rodrigues PG, Ruf I, Schultz CL. Digital reconstruction of the otic region and inner ear of the non-mammalian cynodont Brasilitherium riograndensis (Late Triassic, Brazil) and its relevance to the evolution of the mammalian ear. J Mammal Evol. 2013;20: 291-307.

65. Moore WJ. The mammalian skull. Cambridge: Cambridge University Press; 1981.

66. Presley R. Development and the phylogenetic features of the middle ear region. In: Szalay FS, Novacek MJ, McKenna MC, editors. Mammal phylogeny: Mesozoic differentiation, multituberculates, monotremes, early therians, and marsupials. New York: Springer; 1993. pp. 21-29.

67. Zeller U. Ontogenetic evidence for the cranial homologies in monotremes and therians, with special reference to *Ornithorhynchus*. In: Szalay FS, Novacek MJ, McKenna MC, editors. Mammal phylogeny: Mesozoic differentiation, multituberculates, monotremes, early therians, and marsupials. New York: Springer; 1993. pp. 95-107.

68. Christensen-Dalsgaard J. Vertebrate pressure-gradient receivers. Hearing Res. 2011;273: 37-45.

69. Christensen-Dalsgaard J, Manley GA. Acoustical coupling of lizard eardrums. J Assoc Res Otolaryngol. 2008;9: 407-416.

70. Köppl C. Evolution of sound localisation in land vertebrates. Curr Biol. 2009;19: 635- 639.

71. Heffner HE, Heffner RS. The evolution of mammalian sound localization. Acoustics Today. 2016;12: 20-27.

72. Nothwang HG. Evolution of mammalian sound localization circuits: a developmental perspective. Prog Neurobiol. 2016;141: 1-24.

73. Olson EC. Origin of mammals based upon cranial morphology of the therapsid suborders. Spec Pap Geol Soc Am. 1944;55: 1-136.

74. Luo Z, Crompton AW. Transformation of the quadrate (incus) through the transition from non-mammalian cynodonts to mammals. J Vetebr Paleontol. 1994;14: 341-374.

75. Bonaparte JF, Crompton AW. A juvenile probainognathid cynodont skull from the Ischigualasto Formation and the origin of mammals. Rev Mus Arg Cien Nat Bernardino Rivadavia Inst Nac Invest Cienc Nat. 1994;1: 1-12.

76. Kermack KA, Musset F, Rigney HW. The skull of *Morganucodon*. Zool J Linn Soc. 1981;71: 1-158.

77. Rougier GW, Wible JR, Hopson JA. Reconstruction of the cranial vessels in the Early Cretaceous mammal *Vincelestes neuquenianus*: Implications for the evolution of the mammalian cranial vascular system. J Vertebr Paleontol. 1992;12: 188-216.

78.. Forasiepi AM, Rougier GW. Additional data on early Paleocene metatherians (Mammalia) from Punta Peligro (Salamanca Formation, Argentina): comments based on petrosal morphology. J Zool Syst Evol Res. 2009;47: 391-398.

79. Rougier GW, Wible JR, Hopson JA. Basicranial anatomy of *Priacodon fruitaensis* (Triconodontidae, Mammalia) from the Late Jurassic of Colorado, and a reappraisal of mammaliaform interrelationships. Am Mus Novit. 1996;3183: 1-38.

80. Luo Z, Ruf I, Schultz JA, Martin T. Fossil evidence on evolution of inner ear cochlea in Jurassic mammals. Proc R Soc Lond B. 2011;278: 28-34.
